# Vibrational Property Tuning of MXenes Revealed by Sublattice N Reactivity in Polar and Nonpolar Solvents

**DOI:** 10.1021/jacs.4c13878

**Published:** 2025-02-04

**Authors:** Ray M. S. Yoo, Bright Ngozichukwu, David Kumar Yesudoss, Hao-En Lai, Kailash Arole, Micah J. Green, Perla B. Balbuena, Abdoulaye Djire

**Affiliations:** † Artie McFerrin Department of Chemical Engineering, Texas A&M University, College Station, Texas 77843, United States; ‡ Department of Materials Science & Engineering, Texas A&M University, College Station, Texas 77843, United States; § Department of Chemistry, Texas A&M University, College Station, Texas 77843, United States

## Abstract

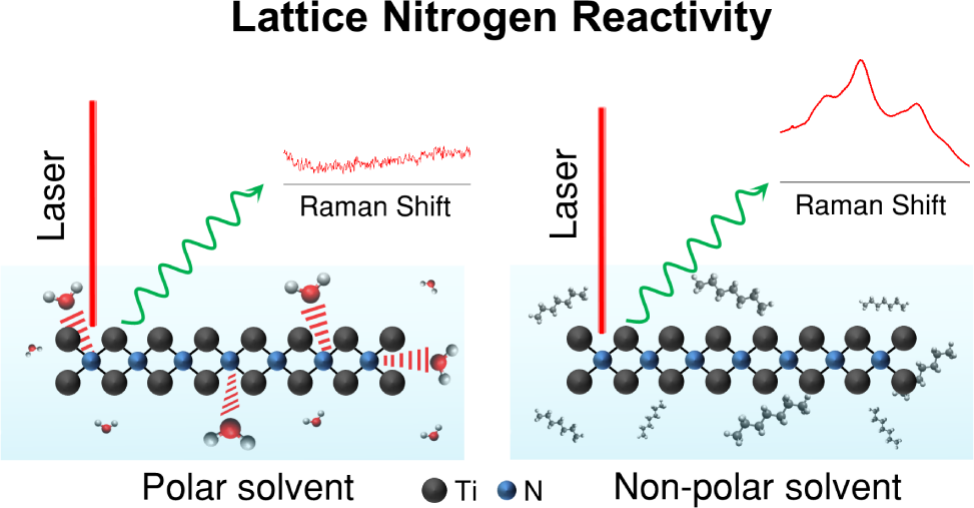

MXenes, a family of two-dimensional (2D) materials based on transition metal carbides and nitrides, have desirable properties, such as high conductivity, high surface area, and tunable surface groups, for electrocatalysis. Nitride MXenes, in particular, have shown excellent electrocatalytic performance for the nitrogen and oxygen reduction reactions, but a fundamental understanding of how their structures evolve during electrocatalysis remains unknown. Equally important and yet unknown is the effect of the reactivity of the lattice nitrogen on the vibrational behavior of nitride MXenes and the resulting implications in electrocatalysis. Here, we investigate the reactivity of lattice nitrogen and the vibrational properties of titanium nitride MXenes in relevant electrocatalytic solvents using confocal Raman spectroscopy. We found that the vibrational modes of titanium nitride MXenes are attenuated in polar solvents, which is revealed through the alteration of the Raman scattering in solvents. Contrary to polar solvents, the vibrational modes remain unchanged in nonpolar solvents like hydrocarbons due to the inactivity of the lattice nitrogen. We found that this behavior is unique to nitrides because the Raman characteristics of carbides and sulfides are unaffected by the solvent types. However, the inclusion of nitrogen into the carbide structure does exhibit Raman-solvent behavior similar to that of nitrides, suggesting that replacing carbon with nitrogen affects MXene–light interactions. We demonstrated a proof-of-concept utilizing lattice nitrogen reactivity to enhance the electrocatalytic nitrogen reduction reaction for ammonia production. In summary, we elucidate the vibrational properties of nitride MXenes in solvents and demonstrate the tunability of MXene vibrational properties via lattice atom substitution, which in turn can be exploited to advance the applications of MXenes in electrocatalysis.

## Introduction

Sustainable and renewable energy technologies are in high demand because of the increased global energy production and consumption associated with greenhouse gas emissions.^1,2^ Electrocatalysis involving aqueous media, such as the hydrogen evolution reaction (HER), oxygen reduction reaction (ORR), and nitrogen reduction reaction (NRR), has been proposed as an alternative resource for the production of green energy and chemicals due to its environmental friendliness.^3−5^ However, for electrocatalytic methods to be viable, efficient electrocatalysts are needed. The current benchmark catalysts for these electrocatalytic reactions involve scarce and cost-inefficient materials.^6−16^ MXenes, a novel family of 2D transition metal carbides, nitrides, and carbonitrides with the chemical formula M_*n*_X_*n*–1_T_*x*_ (M = early transition metal atom, X = C or N, *n* = 2, 3, or 4, and T_*x*_ = −O–, −OH, or −F),^17,18^ have gained attraction as alternative candidates due to their promising properties.^19^ MXenes possess metal-like intrinsic conductivity reaching ∼20 000 S cm^–1^,^17^ which leads to promising electrochemical properties. There have been many reported works based on MXene composite electrodes providing high performance for battery, energy storage, and electrocatalytic applications.^20−27^ Furthermore, the wide range of possible MXene combinations allows for highly tunable chemical compositions, offering significant control over their properties.^28^ This enables MXenes to be tailored for specific applications in renewable energy, making them promising alternative candidates to conventional electrocatalysts. Though hundreds of MXenes have been theoretically predicted and computationally studied to date,^17,29,30^ the experimentally synthesized MXenes are fewer. Among these, nitride MXenes have been reported to be favorable for electrochemical applications, as they are theoretically predicted to possess higher conductivity compared to the more commonly studied carbide MXenes.^31,32^ Corresponding experimental works also underscore the important role of nitride MXenes in pushing MXene electrocatalysis to a benchmark performance. Recently, Djire et al. reported a relatively low overpotential of 300 mV at a benchmark current density of −10 mA cm^–2^ using Ti_4_N_3_T_*x*_ for the HER in acidic electrolytes,^33^ significantly outperforming the Ti_3_C_2_T_*x*_ carbide counterpart.^34,35^ Another experimental work by Johnson et al. reports the Ti_2_NT_*x*_ having a Faradaic efficiency (FE) of 19.85% for the NRR, significantly outperforming the Ti_3_C_2_T_*x*_ carbide MXene (FE = 0.58%).^36^ Moreover, Pranada et al. demonstrated better ORR activity and stability of the Ti_2_NT_*x*_ MXene than that of the Ti_3_C_2_T_*x*_ carbide counterpart, with a lower overpotential and higher kinetic current density.^37^ Nevertheless, the optimization of nitride MXenes remains crucial for further advancing the field of electrocatalysis.

Proper characterization of the chemical structure and vibrational properties, along with the determination of optimal conditions, is essential, although often overlooked, for optimizing materials. Through a scattering process that directly depends on the vibrational modes of chemical bonds involved within, Raman spectroscopy has emerged as a widely used characterization technique that can measure, quantify, and elucidate both structural and vibrational properties of a material in real-time. More importantly, identifying the fundamental structural properties of an electrocatalyst through vibrational excitation provides a basis for understanding its electrocatalytic behavior and opens up routes for further optimization. For a material’s vibrational modes to appear in a Raman spectrum, they must be Raman-active, which requires the associated chemical bonds to experience changes in molecular polarizability during vibration.^38^ Specifically, for 2D materials, it has been reported that the Raman scattering intensity of vibrational modes can be dependent upon certain conditions. For instance, in the case of MoS_2_, the scattering intensity of certain vibrational modes is dependent on the laser wavelength.^39−43^ Leveraging this change provides deeper insight into the structural properties that influence HER electrocatalysis, thereby unlocking new opportunities for optimization. Similarly, by activating the resonance properties of the Ti_3_C_2_T_*x*_ MXene, certain vibrational modes can be further quantified, such as the A_1g_(C) mode that is directly involved with HER electrocatalysis.^18^

Currently, numerous works have reported the characterization of Ti-based nitride MXenes, which have been utilized for electrocatalytic studies.^33,36,37,44−49^ Raman spectroscopy has revealed fundamental vibrational modes and structural properties in these materials, with Ti-based nitride MXenes exhibiting high Raman activity. However, significant gaps remain in understanding the fundamental vibrational behavior of nitride MXenes, particularly under conditions relevant to electrocatalysis. In particular, the vibrational behavior of nitride MXenes in electrocatalytically relevant solvents remains poorly characterized. Understanding the vibrational behavior in solvents provides fundamental insights into the material–solvent interactions directly tied to the electrocatalytic phenomena, which are important for optimizing the corresponding electrocatalytic processes. Previous studies have indicated that, although the reactivity of the lattice nitrogen is inherently low, it can be modulated by varying the elemental composition of the nitride material in question.^50^ This should be taken into serious consideration, as the reactivity can lead to the formation of various bonds with the surrounding solvent, thereby changing the resulting system involved in the corresponding electrocatalytic process. To date, no previous studies have considered this potential impact when studying nitride MXene systems in solvents. Changes in the chemical bonds involved in the vibration process can alter Raman scattering, affecting the visualization of chemical changes under real-time electrolytic conditions. This complicates the conditions required for accurately quantifying certain vibrational properties of nitride MXenes. Unlike other previously studied electrocatalyst materials, which have not encountered such challenges,^39,43,51−56^ this issue is unique to transition metal nitride (TMN)-based materials, as we will demonstrate later in this work. These gaps must be properly addressed for further optimization of nitride MXenes.

In this study, we utilize Raman spectroscopy as a technique to reveal the modified vibrational behavior of nitride MXenes in the presence of solvents, originating from the changes in the Raman scattering process. We provide a comprehensive characterization of Ti-based nitride MXenes in different polar and nonpolar solvents, demonstrating significant differences in Raman scattering. These modifications are shown to be independent of the system parameters, such as laser wavelength, laser power, and acquisition time, and are specific to nitride MXenes and TMN-based materials in general. Our findings provide an indication of the reactivity of the nitrogen atom within the material structure, leading to the formation of weak Raman scattering bonds. Furthermore, we show that the vibrational properties of MXene electrocatalysts in solvents can also be tuned by altering the X atom. This is supported by comparisons between Ti_3_C_2_T_*x*_, N-doped Ti_3_C_2_T_*x*_, and Ti_3_CNT_*x*_ carbonitride MXene, offering insights into structure–property relationships. To further validate our findings, we explore the impact of lattice nitrogen reactivity in hydrocarbon systems, demonstrating that delamination methods and atomic layers of nitride MXenes introduce additional external complexities. Ultimately, we reveal the reactivity of the sublattice nitrogen atom through the characterization of nitride materials in polar and nonpolar solvents using Raman spectroscopy.

## Experimental Methods

### Synthesis of Ti_2_AlN MAX

Ti was mixed and ground with AlN using a mortar and pestle in a 2:1 molar ratio. This mixture was transferred to a crucible and placed into a furnace (CM Furnaces, Inc. 1730–20 HT). After undergoing Ar (Airgas, Ultra High Purity) flow at a flow rate of 400 mL/min for 30 min, the furnace was heated up to 1600 °C at a ramp rate of 10°C/min. Once 1600 °C was reached, the temperature was held for 1 h. The furnace was then cooled stepwise: 1300 °C for 20 min, then to 1100 °C for 20 min, and finally to 900 °C for 20 min. Afterward, the furnace was allowed to cool down to room temperature, and the Ar flow was stopped after reaching 200 °C. The resulting material was then stored in a vial under an inert atmosphere for future analysis and for the subsequent synthesis of Ti_2_NT_*x*_ MXene.

### Synthesis of Ti_2_NT_*x*_ MXene

The parent Ti_2_AlN MAX phase was added to a 59:29:12 wt % molten salt fluoride (MSF) mixture of KF (Alfa Aesar, 99%), NaF (Alfa Aesar, 99%), and LiF (Alfa Aesar, 325 mesh, 98.5%), with the resulting mixture in a 1:1 weight ratio with respect to the MAX and the MSF mixture. After the mixture of MAX + MSF was ground with a mortar and pestle, it was then transferred to a crucible and then placed into the furnace (CM Furnaces Inc. 1730–20 HT). The furnace was heated up to 550 °C at a ramp rate of 10°C/min and under Ar (Airgas, Ultra High Purity) flow at a flow rate of 400 mL/min. After reaching 550 °C, the furnace was held at this temperature for 5 h to initiate Al etching from the MAX through the formation of Al-based fluoride compounds. During the halfway point, the Ar flow was stopped, allowing oxygen to enter the furnace and react with the Al-based fluoride compounds to further etch the Al from the MAX structure. Oxygen reacts with Al to form Al oxides, which are then removed by the reaction with fluoride salts. After the end of 5 h, the furnace was cooled to 450 °C over 12 min and held at this temperature for another 40 min. Finally, the furnace was cooled to room temperature. Afterward, the weigh boat was extracted, and the molten salt-treated Ti_2_AlN MAX material (Ti_2_AlN-MST) was collected and transferred to a vial. The Ti_2_AlN-MST underwent an acid wash with formic acid (Sigma-Aldrich, 95%) for 1 h. After this, the resulting mixture underwent vacuum filtration onto a Celgard 3501 membrane with ultrapure water until the material achieved neutral pH. The synthesized multilayered Ti_2_NT_*x*_ MXene was then mixed with ultrapure water and sonicated for 30 min to delaminate the material. After sonication, the mixture was left to sit for 1 to 2 h, with the supernatant then extracted to undergo a final vacuum filtration. The filtered material (few-layered Ti_2_NT_*x*_) was then collected and dried at 50 °C under vacuum overnight. This material was then stored in a vial under an inert atmosphere for future analysis. Alternatively, following vacuum filtration, the synthesized multilayered Ti_2_NT_*x*_ MXene was mixed with 10 mL of dimethyl sulfoxide (DMSO) (Sigma-Aldrich, ACS Reagent). The mixture was stirred for 18 h and then was transferred to a centrifuge vial to be sonicated for 30 min. The solution was then filled to 50 mL with water and centrifuged at 3500 rpm (1962 RCF) for 30 min followed by decanting. This same process was repeated until a neutral pH was reached. The vial was then filled with water to the 10 mL mark, shaken, and left to sit for 1 to 2 h, with the supernatant then extracted to undergo a final vacuum filtration. This filtered few-layered Ti_2_NT_*x*_ was then collected and dried at 50 °C under vacuum overnight. This material was then stored in a vial under an inert atmosphere for future analysis.

### Synthesis of Ti_4_AlN_3_ MAX

The synthesis of the Ti_4_AlN_3_ MAX phase involved the mixing of Ti (Sigma-Aldrich, 99.7%, 100 mesh), Al (Sigma-Aldrich, 99%, 30 μm), and TiN (Sigma-Aldrich, 99%, 3 μm) powders in a molar ratio of 1:1.2:2.05. This mixture was ground in an agate mortar for a duration of 10 min. Subsequently, the resulting mixture was subjected to sintering in a tube furnace (CM Furnace Inc. 1730–20 HT) at a temperature of 1500 °C for a duration of 12 h, with a gradual ramp rate of 10 °C/min, while maintaining a constant flow of argon (Ar) gas (Airgas, Ultra High Purity). The resultant Ti_4_AlN_3_ pellet was then ground in an agate mortar in preparation for etching.

### Synthesis of Ti_4_N_3_T_*x*_ MXene

The molten salt treated Ti_4_AlN_3_ (Ti_4_AlN_3_-MST) material was first produced by selectively etching Al from the Ti_4_AlN_3_ MAX phase through an oxygen-assisted molten salt fluoride etching treatment. The MSF mixture containing KF (99%, Alfa Aesar), LiF (99%, Alfa Aesar, 325 mesh, 98.5%), and NaF (99%, Alfa Aesar) in a 59:29:12 mass ratio was mixed with Ti_4_AlN_3_ powder in a 1:1 mass ratio. The mixture of MAX + MSF was ground with a mortar and pestle and then transferred to a crucible and placed into the furnace (CM Furnaces Inc. 1730–20 HT). The furnace was heated to 550 °C at a ramp rate of 10 °C/min and held for 5 h under argon flow. After 5 h, the argon flow was discontinued, and the 3/16’’ tubing of the furnace outlet was exposed to the air for a duration of 1 h. The furnace was then sealed from the atmosphere and allowed to continue etching for another 2 h. Finally, the furnace was turned off and left to cool to room temperature, after which the Ti_4_AlN_3_-MST material was collected and ground for a uniform particle size distribution. Approximately 0.5 g of the etched Ti_4_AlN_3_-MST sample was acid-washed by combining it with 20 mL of 4 M formic acid (Sigma-Aldrich, 95%) in a beaker. The solution was stirred at 500 rpm for 1 h. The resulting solution was then filtered through a 0.20 μm polycarbonate membrane (Whatman Nucleopore) and rinsed continuously using deionized water (18.2 MΩ·cm, Milli-Q) until a pH of 6 was achieved. After the wash cycle, the Ti_4_N_3_T_*x*_ sample was collected, dried in a vacuum oven at 50 °C overnight, transferred to a vial, and stored in a glovebox. To form delaminated Ti_4_N_3_T_*x*_ MXene, 10 mL of tetramethylammonium hydroxide (TMAOH) was mixed with the ML Ti_4_N_3_T_*x*_ sample and stirred for 4 h. The resulting solution was then sonicated for 30 min, then diluted with water to reach a volume of 50 mL. Centrifugation at 3500 rpm for 30 min was performed, followed by decanting the supernatant. This process was repeated until a neutral pH was achieved. Subsequently, the solution was left for 1 h after filling the vial with water up to 10 mL, to allow any remaining MAX to precipitate. The resulting supernatant was filtered onto a 0.20 μm Celgard 3501 membrane (Whatman Nucleopore) and dried at 50 °C under vacuum overnight to obtain a delaminated MXene powder (FL Ti_4_N_3_T_*x*_).^57^

### Synthesis of Ti_3_AlC_2_ MAX

The Ti_3_AlC_2_ MAX phase was synthesized using Ti (average particle size of 44 μm, 99.5% purity), Al (average particle size of 44 μm, 99.5% purity), and TiC powders (average particle size of 2–3 μm, 99.5% purity). All of these chemicals were procured from Alfa Aesar, MA, USA. Ti, Al, and TiC powders were first weighed to obtain a ratio of Ti:Al:C = 3.0:1.2:1.8 and mixed for 24 h at 300 rpm in a glass jar using ball milling with zirconia beads. The high-purity Ti_3_AlC_2_ samples were sintered using a pulsed electric current system (PECS) at a temperature of 1510 °C with a loading of 50 MPa for 15 min. These Ti_3_AlC_2_ samples were then drill-milled and sieved to obtain a powder with particle sizes below 45 μm.

### Synthesis of Ti_3_C_2_T_*x*_ MXene and N-Doped Ti_3_C_2_T_*x*_ MXene

Initially, lithium fluoride (0.8 g) was slowly added to 6 M hydrochloric acid (10 mL) under constant magnetic stirring at 40 °C. The Ti_3_AlC_2_ powder (1 g) was gradually added to a mixture of LiF and HCl at 40 °C for 40 h under constant magnetic stirring. The Ti_3_AlC_2_ powder was added very slowly. After 40 h, the mixture was transferred to centrifuge vials and centrifuged at 9000 rpm for 15 min to remove the HF. The sediment was then redispersed in water for washing, and the water washing was performed 3–4 times to obtain neutral pH. Later, the sediment was again dispersed in DMSO for intercalation and stirred for 20 h. After that, the mixture was washed with water 2–3 times, and finally, the sediment was dispersed in water for bath sonication (1 h). The delaminated Ti_3_C_2_T_*x*_ was collected in the supernatant after the final centrifugation at 3500 rpm for 55 min and then freeze-dried for about 24 h to obtain Ti_3_C_2_T_*x*_ nanosheets.

To produce the N-doped Ti_3_C_2_T_*x*_ MXene materials, the synthesized Ti_3_C_2_T_*x*_ MXene was exposed to 100 mL min^–1^ of ammonia gas for 2 h at 700 °C.

### Synthesis Procedure for Ti_3_CNT_*x*_ MXene

The Ti_3_CNT_*x*_ MXene was prepared through a mild acid etching method using LiF and H_2_SO_4_. Initially, 0.5 g of the Ti_3_AlCN MAX phase was slowly dispersed in a solution comprising 10 mL of 9 M H_2_SO_4_ and 0.8 g of LiF, with stirring at 400 rpm. After stirring for 24 h, the mixture was washed through centrifugation with DI water at 4000 rpm until a neutral pH was achieved. The separated solid particles containing Ti_3_CNT_*x*_ MXene and unetched MAX phases were redispersed in DI water and sonicated for 1 h, followed by a 1 h settling period to allow the heavier MAX phases to settle. The supernatant was then carefully collected and filtered through a 0.10 μm porous membrane using vacuum-assisted membrane filtration. The resulting film was dried in a vacuum oven at 50 °C for 24 h, followed by grinding to produce Ti_3_CNT_*x*_ MXene powders.

### Characterization

The crystalline structure of the material was analyzed by using X-ray diffraction (XRD, Rigaku Miniflex 6G). All XRD measurements were run from 3 to 70° at 2°/min. Surface characterization was performed using X-ray photoelectron spectroscopy (XPS, Omicron XPS system with Argus detector courtesy of TAMU Materials Characterization Facility) (DAR400, RRID: SCR_022202) and Raman spectroscopy (Renishaw inVia Qontor). For survey scans, XPS analysis was done with the CAE at 100 eV and the dwell time at 0.05 s. For high-resolution scans, XPS analysis was done with the CAE at 40 eV and the dwell time at 0.05 s, with three spectra collected to be averaged out for the overall scan. For the X-ray 558 control, the emission current was set to 15 mA and the anode current was set to 15 kV, making the X-ray power of 225. For the CN10 neutralizer settings, the emission current was set at 10 μA and the beam energy at 2 eV. The aperture was set at 3 or 5, making the aperture coefficients *a* and *b* of 304.3 and 0.91, and 39.2 and 0.43, respectively. All Raman characterizations were performed with a 532 nm laser, 1800 lines per mm grating, and a 50× L objective lens, except when stated. All Raman characterizations of MXenes were performed with few-layered materials. Raman characterizations of fresh and dried electrocatalyst materials were collected on a flattened material on a microscope slide. Studies involving immersion in solvents were done by adding a droplet of the solvent of interest in the same system. Characterizations of the few-layered Ti_2_NT_*x*_ MXene were primarily done with those synthesized from the multilayered Ti_2_NT_*x*_ MXene being delaminated with water, unless otherwise stated.

## Computational Methods

### Ab Initio Molecular Dynamics Simulation Computation Details

All Ab Initio Molecular Dynamics Simulations were performed using first-principles methods implemented in the Vienna Ab initio Simulation Package (VASP). The electronic structure calculations employed density functional theory (DFT) with the generalized gradient approximation (GGA) using the Perdew–Burke–Ernzerhof (PBE) functional for electron correlation, while electron–ion interactions were treated using projector augmented wave (PAW) pseudopotentials. AIMD simulations were conducted to investigate dynamic behavior and bond length changes in different environments of different facets of Ti_2_NT_*x*_ and AlN. Brillouin zone sampling was conducted using the Monkhorst–Pack method with a 2 × 2 × 1 *k*-point grid for all the slab systems. The calculations employed a plane-wave cutoff energy of 450 eV and 10^–4^ eV for the electronic energy convergence criterion, using a Gaussian smearing width of 0.1 eV. Van der Waals interactions were considered by empirical DFT-D3 correction. Spin polarization was incorporated to accurately represent the electronic structure of Ti_2_NT_*x*_. All AIMD simulations were performed in the NVT ensemble at 298 K. Temperature control was maintained using a Nosé thermostat with a mass parameter of 0.5, and trajectories were propagated using 1 fs time steps for a total simulation time of 1 ps. The simulation cell was cleaved by expanding the unit cell from our previous work.

### Ti_2_NT_*x*_ and AlN Slab Model Construction

The slab simulation model was relaxed using the supercell of our previous work’s unit cell. Three surface termination configurations of Ti_2_NT_*x*_ were investigated: Ti_2_NO_2_ axial surfaces with 25% −OH terminations and Ti_2_NO_2_ axial surfaces with 75% −OH terminations (consistent with experimental Raman spectroscopy). As for the AlN surface, two facet surface configurations of AlN were investigated, including a [001] facet (0001 in Miller-Bravais notation) and a [110] facet (0110 in Miller-Bravais notation). These two facets show the best match between our experimental Raman data and previous single-crystal Raman peaks in previous work on different AlN facets. All slab facets were computed with a 5-layer slab with 2 fixed bottom layers.

### Solvent Environment Modeling

A 15 Å vacuum layer was introduced following surface cleavage to prevent periodic image interactions. Various solvent environments were simulated by using pure water to replicate experimental conditions. The number of solvent molecules was determined based on density values at 298 K: water (0.9970 g cm^–3^), hexane (0.6606 g cm–3), and acetone (0.7845 g cm^–3^) (CRC Handbook of Chemistry and Physics). Solvent molecules were incorporated using the amorphous packing tool in Materials Studio Software. The solvent structure relaxation protocol involved initial dynamics with 1 fs time steps, with a total of 2–3 ps for the Ti_2_NT_*x*_ axial surface and around 2–4 ps for the AlN surface to obtain initial relaxation and reaction of solvent on the surface.

### Hydrogen Bonding Analysis

Quantitative analysis of hydrogen bonding interactions was implemented through a multicriteria computational approach, with careful exclusion of bottom facets to focus on relevant surface phenomena for the Ti_2_NT_*x*_ axial surface. The characterization protocol encompassed three distinct metrics for hydrogen bond identification and quantification. First, pairwise distance calculations were performed between oxygen atoms to quantify solvent oxygen atoms proximate to Ti-coordinated oxygen species, utilizing a 3.1 Å distance criterion. Second, the spatial correlation between hydrogen atoms from Ti–OH terminal groups and solvent oxygen atoms was evaluated using a 2.2 Å distance threshold for hydrogen bond formation. Third, oxygen atoms from Ti–O terminal groups were assessed for their interaction with hydrogen atoms from H–O moieties in the solvent phase, employing a 2.2 Å cutoff for hydrogen bond identification. This systematic approach enables precise quantification of the hydrogen bonding network architecture and its evolution across different surface configurations and solvent environments.

## Results and Discussion

**Figure 1:**
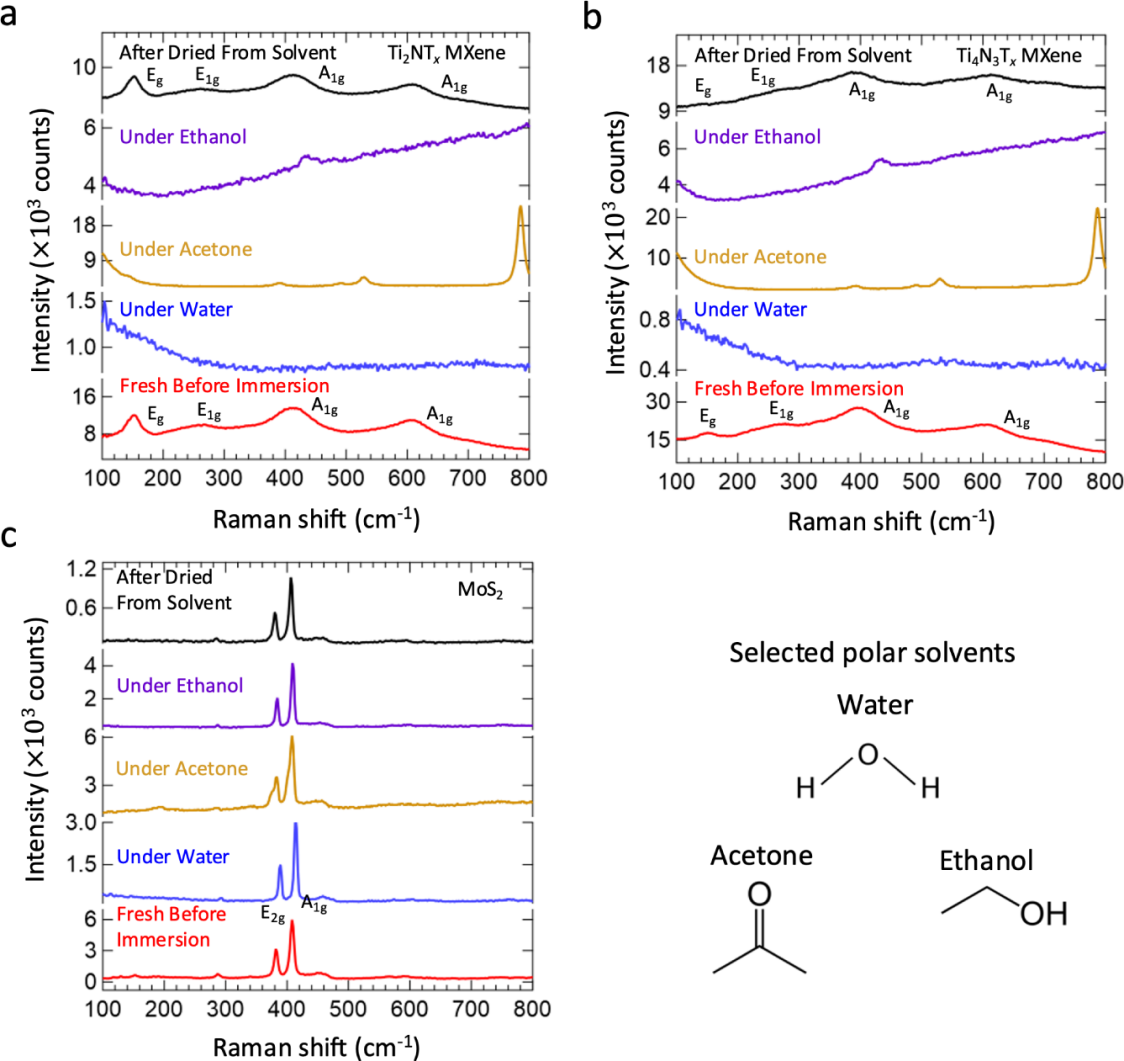
Raman spectra of a. Ti_2_NT_*x*_, b. Ti_4_N_3_T_*x*_ MXenes, and c. MoS_2_ electrocatalyst fresh before immersion (red), in water (blue), in acetone (gold), in ethanol (violet), and after drying from solvent (black). The spectra for the Ti_2_NT_*x*_ and Ti_4_N_3_T_*x*_ MXenes were obtained at 100% laser power to access the MXene subsurface structure and for equal comparisons of the nitride MXene materials when dry and when immersed in solvents. The spectra for MoS_2_ were obtained with 10% laser power. All spectra were collected using the 532 nm laser, 1800 lines/mm grating, and a 50× objective lens.

We provide a fundamental characterization of few-layered Ti-based nitride MXenes (Ti_2_NT_*x*_ and Ti_4_N_3_T_*x*_) using Raman spectroscopy. Few-layered materials were selected for their enhanced surface area as they are optimal for electrocatalytic studies. XRD data (Figure S1a,b) confirm the successful synthesis of the few-layered Ti-based nitride MXenes from the starting MAX phases, as evidenced by the red shift of the (002) XRD peak. The few-layered morphology and high crystallinity of the Ti_2_NT_*x*_ MXene were further supported by transmission electron microscopy (TEM) images (Figure S2a,b). Figure 1a,b (red trace) shows the obtained Raman spectra of the Ti_2_NT_*x*_ and Ti_4_N_3_T_*x*_ MXenes, in which the observed vibrational modes are consistent with previously reported studies.^33,36,44−48^ The nitride MXenes are highly Raman active, as evidenced by the strong Raman scattering and high-intensity counts in the spectra. We then focus on the characterization of these materials in water (Figure 1a,b, blue trace), a baseline solvent for electrocatalytic applications. The scattering intensity of the vibrational modes for nitride MXenes becomes nearly nonexistent, with the background spectrum of water dominating (Figures 1a,b and S3, blue trace). This shift in Raman activity indicates a modification in the vibrational behavior of the material in the solvent. We confirm that the material is being probed correctly, as indicated by the depth acquisition experiment (Figure S4a), and increasing the acquisition time does not recover the vibrational modes of nitride MXenes (Figure S4b). We also ensure that the material is focused correctly on the spectrometer. To further validate that these vibrational properties are indeed modified, we performed Raman characterization on MoS_2_, a widely studied electrocatalyst, in the same solvent system (Figure 1c, blue trace). The Raman activity and vibrational behavior of MoS_2_ remain unchanged, as evidenced by the retention of its spectrum, demonstrating that the experimental setup is not responsible for the observed phenomenon that is specific to nitride MXenes. Given that a change in the Raman scattering process involved could possibly indicate a change in the nature of the chemical bonding tied to the vibrational process, it is essential to further investigate this phenomenon. Thus, we extend the Raman characterization to Ti-based nitride MXenes in other common polar solvents, such as acetone and ethanol (Figures 1a,b and S3, gold and purple traces). In these solvents, the Raman spectrum corresponds to the solvent, with the vibrational modes of the nitride MXenes absent. This suggests that polar solvents can induce changes in the vibrational behavior of nitride MXenes through some form of interaction. Notably, this phenomenon occurs only in the presence of solvents, as the vibrational modes of nitride MXenes are fully recovered once the materials are dried (Figure 1a,b, black trace). This finding provides key initial insights into the modified vibrational behavior of nitride MXenes in solvents, suggesting a reversible interaction between the nitride MXenes and the solvent. In other words, the vibrational properties of nitride MXenes are temporarily modified during immersion but return to their original state once the solvent interaction ceases.

To further verify this, we characterize a thin Ti_2_NT_*x*_ MXene layer on a silicon (Si) wafer, which exhibits a characteristic Raman peak at 520 cm^–1^ (Figure S5). We hypothesize that in the absence of Raman scattering in solvents and if the MXene layer is thin enough, the resulting spectrum should reflect the Raman scattering of the underlying Si substrate, along with that of the solvent. We first confirm the characterization of a dry, thin Ti_2_NT_*x*_ MXene layer on a Si wafer, as shown by its inherently high Raman activity (Figure S5a). When this thin layer of Ti_2_NT_*x*_ MXene on a Si wafer is immersed in acetone, only the Raman spectrum corresponding to acetone and the Si wafer is obtained (Figure S5b). The resulting spectrum is identical to that of the bare Si wafer in acetone, confirming that the Raman scattering from the nitride MXenes is absent due to the alteration caused by the solvent. Additionally, we observe that variations in laser power affect the relative intensity ratio of the Si Raman peak to the acetone spectrum (Figure S5b). Specifically, the intensity ratio increases with laser power as a deeper section of the Si wafer is probed. For the results presented in Figures 1a,b and S5c, only the solvent spectrum is accounted for, as the nitride MXene layer is thickened by a few hundred micrometers, preventing the laser from probing the wafer. The system involving a thin layer of nitride MXene on a Si wafer directly highlights the phenomenon of the absence of Raman scattering for nitride MXenes in solvents, further corroborating previous findings. Further corroboration is provided by characterizing the Ti_2_NT_*x*_ MXene material in DMSO, a polar organic solvent with a significantly higher boiling point. This allows us to slow the drying process, enabling the material to reach a state where some spots are dried while others remain undried. Based on the findings from Figure 1, the type of spot can be identified by Raman spectroscopy, depending on the spectrum obtained. From the dried spots, we clearly observe that only a very short acquisition time is required to obtain the Ti_2_NT_*x*_ MXene Raman spectrum (Figure S6a), consistent with the inherent Raman scattering process of the Ti_2_NT_*x*_ MXene that results in high Raman activity over a short period of acquisition time. Notably, increasing the acquisition time for the undried spots does not recover the Ti_2_NT_*x*_ MXene spectrum but instead enhances the Raman scattering from DMSO (Figure S6b). The result for the undried spots is expected, as the absence of a Raman scattering process from the Ti_2_NT_*x*_ MXene means that increasing the acquisition time will not alter the spectrum. These results are consistent with the Raman scattering behavior observed for nitride MXenes when dried or immersed in water, acetone, and ethanol. In particular, the long acquisition time for undried spots contrasts sharply with the very short acquisition time needed for the characterization of the dried spots. This Ti_2_NT_*x*_ MXene-DMSO system thus directly demonstrates the change in vibrational behavior of nitride MXenes when transitioning from a dry state to immersion in solvents, providing further corroboration of previous results.

Altogether, a reversible interaction between the nitride MXenes and the surrounding solvent molecules seems to induce modifications in the vibrational behavior. To investigate further, we conducted a comparative analysis of the Raman characterization of related bulk nitride materials such as TiN and AlN in water. As expected, TiN shows modified vibrational behavior, indicated by the shift in Raman activity, while surprisingly, the vibrational properties of AlN are retained (Figure S7a,b). This suggests that while the modification of vibrational behavior in solvents is specific to nitride-based materials, it is also dependent on the material’s composition. We then performed a comparative analysis of TiN, AlN, and Ti_2_AlN MAX phases in water (Figure S7a,b). The change in the coordination environment from AlN to Ti_2_AlN MAX induces the modification of the vibrational behavior in solvents. To further understand this behavior, AIMD computational analyses were used, which reveal water splitting at the (001) facet of the AlN in water. Through water splitting, surface aluminum sites predominantly undergo hydroxylation. For N-terminated (001) surfaces, some of the exposed nitrogen sites undergo hydrogenation to form stable N–H terminations, while other exposed nitrogen surfaces repel polar solvents (Figure S8). A complex hydrogen-bonding network between Al–OH and water molecules is formed, while the N–H terminals and exposed nitrogen sites undergo limited hydrogen bonding and repel the water clusters (Figure S8). As hydrogen bonding with water is the main source of interaction that induces the strong polar bonds for alteration in the Raman scattering, AlN provides an opportunity in regard to withholding this related reactivity of lattice nitrogen. Here, although the reactivity of the lattice nitrogen is still observed as usual to form N–H terminals, they are considered to be a weaker source of polar bonds; moreover, the reactivity that induces strong polar bond formation is not observed. Therefore, the absence of the relevant reactivity and interaction of lattice nitrogen in AlN emphasizes that the retention of Raman scattering and vibrational properties is directly connected. The results suggest that nitrogen in the nitride structure is reactive with the solvent molecules, forming strong polar bonds; however, this reactivity is influenced by the composition of the nitride material. From these results, it is evident that the observed modification in vibrational behavior is tied to the reactivity of the lattice nitrogen with the solvent. In other words, the change in the Raman scattering process is due to this reactivity, prompting the modification of the chemical bonds involved in the vibration. Moreover, the reactivity with the solvent can lead to the formation of strong polar bonds, which exhibit minimal polarizability changes during vibration, consistent with the results seen for MXenes in solvents.

A key experimental observation is that even minimal exposure to solvent induces the modification of vibrational properties for nitride MXenes (Figure S9). We also confirm that changes in other parameters, such as laser wavelength and applied laser power, do not influence the observed absence of Raman scattering of nitride MXenes in solvent conditions (Figures S10–S27). When we turn to the 633 and 785 nm laser results, we note that the majority of results for the nitride MXenes immersed in solvents exhibit modified vibrational properties, as indicated by the background solvent spectrum as usual, or exhibit fluorescence, but no retention of the Raman vibrational modes of the nitride MXenes. All these results further emphasize the modified vibrational behavior of nitride MXenes when immersed in solvents. Our findings also suggest that the 532 nm laser is typically the best to characterize the nitride MXenes, as it can selectively characterize the surface and subsurface of nitride MXenes via modulation of the laser power. The penetration depth of the 633 and 785 nm lasers is lower compared to that of the 532 nm laser, as expected due to the difference in wavelength, making them less ideal for decoupling surfaces and subsurfaces. Additionally, we note that, besides the absence of Raman activity, fluorescence typically dominates when the Ti_2_NT_*x*_ MXene is immersed in ethanol. Further investigation of the interaction between Ti_2_NT_*x*_ MXene and ethanol could lead to new applications for MXenes that currently do not exist.

**Figure 2:**
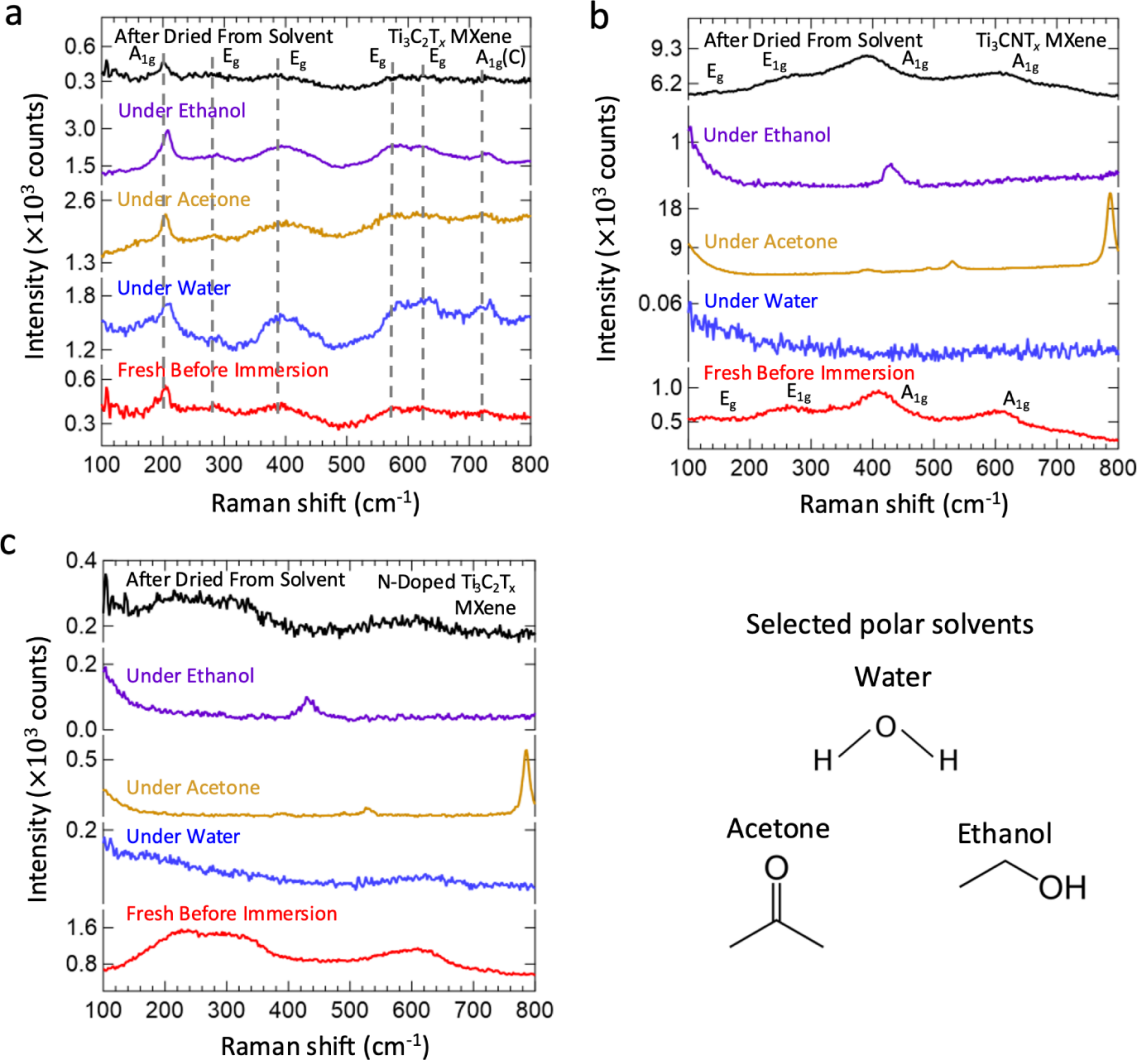
Raman spectra of a. Ti_3_C_2_T_*x*_, b. Ti_3_CNT_*x*_, and c. N-doped Ti_3_C_2_T_*x*_ MXenes fresh before immersion (red), in water (blue), in acetone (gold), in ethanol (violet), and after drying from solvent (black). All spectra were collected using the 532 nm laser, 1800 lines/mm grating, and a 50× objective lens. The spectra for the Ti_3_C_2_T_*x*_ and N-doped Ti_3_C_2_T_*x*_ MXenes were obtained at a 10% laser power. The spectra for Ti_3_CNT_*x*_ were obtained at 100% laser power to access the subsurface MXene structure and for a fair comparison with the nitride MXenes. The effect of nitrogen in the MXene material structure on the modified vibrational properties in solvents is illustrated.

We then turn to the Raman characterization of the benchmark Ti_3_C_2_T_*x*_ MXene in solvents (Figure 2a). We observe that the scattering intensity of the Raman vibrational modes for the Ti_3_C_2_T_*x*_ MXene remains unmodified, in stark contrast to the counterpart Ti-based nitride MXenes. This aligns with previous studies reporting on the Raman characterization of the Ti_3_C_2_T_*x*_ MXene dispersion in organic solvents and previous in situ Raman studies of Ti_3_C_2_T_*x*_ which all show unmodified Raman activity of the carbide MXene.^51,58−62^ From these findings and past work, it can be interpreted that the vibrational behavior of carbide MXenes remains largely unaffected by solvent exposure.^63^ It appears that for Ti-based MXene materials, the carbon atoms are unreactive to solvent molecules, whereas nitrogen atoms exhibit significant reactivity. With this in mind, we explored the potential for modifying the vibrational properties of Ti-based carbide MXenes in solvents by introducing nitrogen into their structure. Thus, it is of interest to observe the effect of the presence of both types of X atoms in the MXene structure, turning to the hybrid Ti_3_CNT_*x*_ carbonitride MXene as a potential transition point. Upon Raman characterization of the Ti_3_CNT_*x*_ MXene in solvents (Figure 2b), we observed a loss of Raman scattering intensity of the vibrational modes, accompanied by the corresponding background solvent spectrum, similar to the behavior seen in nitride MXenes. Likewise, varying the laser power or wavelength does not affect the Raman activity of Ti_3_CNT_*x*_ in solvents (Figures S28–S36). This represents a direct transition from the Ti_3_C_2_T_*x*_ MXene, indicating that while Ti-based carbide MXenes themselves are not reactive with solvent molecules, the introduction of lattice nitrogen enhances reactivity, modifying the Raman scattering and vibrational properties of Ti-based MXenes in solvents. The Ti_3_CNT_*x*_ MXene thus represents a key transition point, illustrating how the alteration of X atoms in the MXene structure can modify the vibrational properties due to the reactivity of lattice nitrogen. Further corroboration of this phenomenon is provided through the nitridation of the starting transition metal oxides TiO_2_ anatase and V_2_O_5_ into the TMNs TiN and VN, respectively (Figure S37a,b). In water, the Raman vibrational modes of TiO_2_ anatase and V_2_O_5_ are retained, while those of TiN and VN are significantly altered, highlighting the effect of the incorporation of the nitrogen atom through nitridation (Figure S37b,c).

There have been previously reported studies related to heteroatom doping, typically for tuning the electronic structure of electrode materials.^64−68^ For MXenes, heteroatom doping can provide a way to identify related effects and implications of associated structural changes, playing an essential role in optimization. For example, a work by Wen etal. reports on doping the Ti_3_C_2_T_*x*_ MXene with nitrogen via ammonization to improve the pseudocapacitive behavior under optimal electrochemical measurement conditions.^69^ For our studies, we expect that the direct application of this method can provide solid corroboration regarding the aforementioned effect of the lattice nitrogen tuning of the vibrational behavior of MXene electrocatalysts in solvents. Hence, we ammonize different Ti_3_C_2_T_*x*_ MXene batches at 700 °C to successfully incorporate nitrogen atoms into the MXene and synthesize two types of N-doped Ti_3_C_2_T_*x*_ MXenes with differing material structures. The structural characterization for the synthesized materials using XPS data (Figure S38a–d) reveals the successful doping of the nitrogen in the Ti_3_C_2_T_*x*_ MXene via the formation of the Ti–N bond. According to the XPS spectra, for one type of N-doped Ti_3_C_2_T_*x*_ MXene (Figure S38a–d), its Ti 2p XPS shows resemblance to that of Ti_3_C_2_T_*x*_ MXene in previous works (Figure S38a), along with the Ti–C bond in the C 1s spectrum (Figure S38c). Nevertheless, nitrogen is still doped into the structure, as evidenced by the Ti–N bond in the N 1s spectrum (Figure S38d). For the other N-doped Ti_3_C_2_T_*x*_ MXene, the structure resembles more passivated nitride MXenes, as evidenced by the Ti 2p spectrum (Figure S38a) and the absence of the Ti–C bond in the C 1s spectrum (Figure S38c). Figures 2c and S39 show the Raman spectra of the N-doped Ti_3_C_2_T_*x*_ MXene materials, providing further insights into the role of nitrogen in the lattice.^67,68^ Notably, the Raman spectrum of the second N-doped Ti_3_C_2_T_*x*_ MXene sample has more resemblance to the Ti-based nitride MXenes. However, some subtle differences such as the modified E_1g_ vibrational mode at around 200 to 300 cm^–1^ and the broad peak spanning from 600 to 750 cm^–1^ can be observed. The vibrational region between 600 to 750 cm^–1^ for Ti_3_C_2_T_*x*_ MXene is dependent upon the C atom, and modifications via nitrogen displacement are expected to result in changes accordingly.^18^ Exact identification of the vibrational modes and further elucidation of the effects of nitrogen doping will be investigated in future works.

**Figure 3:**
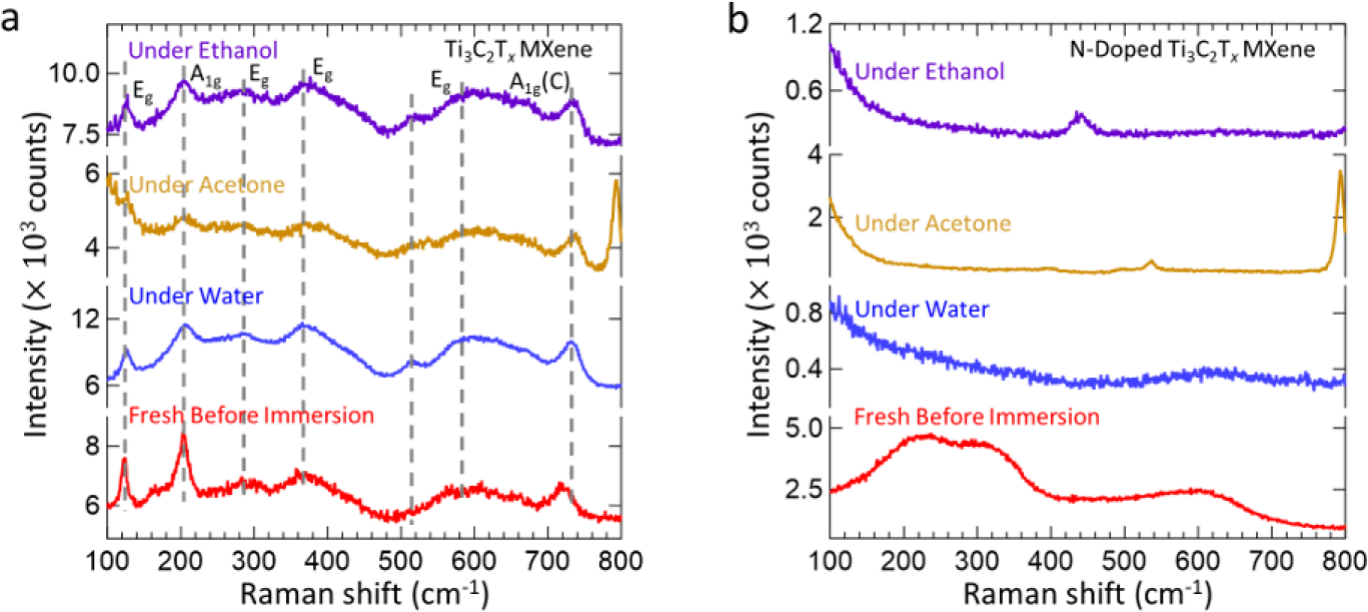
Raman spectra of a. FL Ti_3_C_2_T_*x*_ and b. N-doped FL Ti_3_C_2_T_*x*_ fresh before immersion (red), in water (blue), acetone (gold), and ethanol (violet) with the 785 nm laser. All spectra were collected using the 785 nm laser, 1800 lines/mm grating, and a 50× objective lens. The spectra for the Ti_3_C_2_T_*x*_ and N-doped Ti_3_C_2_T_*x*_ MXenes were obtained at 5% laser power. The inability to activate the resonance conditions for Ti_3_C_2_T_*x*_ to N-doped Ti_3_C_2_T_*x*_ is illustrated.

**Figure 4:**
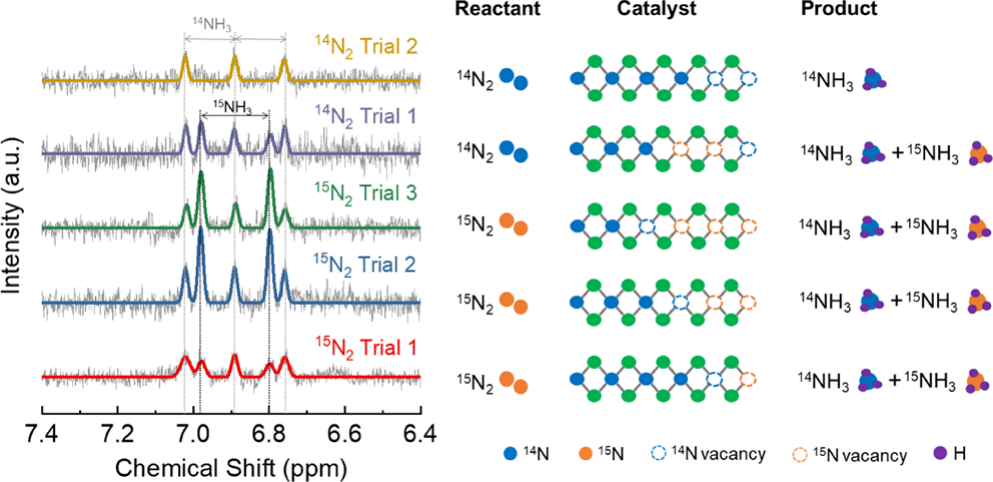
NMR data of NRR electrocatalysis for Ti_2_NT_*x*_ MXene in 0.1 M HCl under different types of N_2_ gas flow. The results indicate the formation of both ^14^NH_3_ and ^15^NH_3_ when only ^15^N_2_ was used as the reactant. Since the only source of nitrogen is from the nitride material and the process is catalytically stable, this further demonstrates how nitrogen reactivity can be exploited in electrocatalysis.

The Raman characterization of the first type of N-doped Ti_3_C_2_T_*x*_ MXene material in solvents, since structurally it resembles starting Ti_3_C_2_T_*x*_ MXene from the XPS data. However, due to the incorporation of nitrogen as verified by Ti–N bonding in XPS, the background solvent spectrum similar to Ti_3_CNT_*x*_ MXene is observed (Figure 2c). Notwithstanding the resemblance between the two material phases, it is clear that the starting Ti_3_C_2_T_*x*_ MXene has its Raman scattering process and related vibrational properties modified in solvents when nitrogen is doped into the structure. Furthermore, application of resonance conditions with the 785 nm laser for the Ti_3_C_2_T_*x*_ MXene provides additional corroboration by being unable to recover the MXene Raman spectrum (Figure 3a,b) with this N-doped Ti_3_C_2_T_*x*_ MXene. Under resonance conditions, Raman scattering enhancement is possible only if the vibrational modes of the material of interest are present and active in the system. This is the case for the Ti_3_C_2_T_*x*_ MXene in solvents, where the scattering intensity of vibrational modes is retained (Figure 3a). The vibrational behavior of Ti_3_C_2_T_*x*_ remains intact, allowing for the expected Raman scattering activity when resonance conditions are applied. However, for the N-doped Ti_3_C_2_T_*x*_ MXene, the vibrational behavior in solvents is completely modified due to the alteration in the Raman scattering process. When the same resonance condition is applied to the N-doped Ti_3_C_2_T_*x*_, no Raman active vibrational modes related to the material are observed. Instead, the spectrum corresponds entirely to that of the solvent (Figure 3b), indicating the absence of any Raman scattering intensity from the material. This observation aligns with the behavior seen for nitride materials, where the reactivity of the lattice nitrogen alters the Raman scattering process. These findings further corroborate the idea that the modification of Raman scattering and vibrational properties is induced by the lattice nitrogen atom. Also, this opens a platform to explore the role of the X atom in tuning the vibrational properties of MXenes in solvents. Understanding these changes in vibrational behavior is crucial for optimizing MXene electrocatalysis and advancing the design of MXene-based materials for various applications. Additionally, understanding the reactivity of lattice nitrogen in electrolytic systems offers valuable insights for enhancing the electrocatalytic performance in several areas. As an illustrative example, we investigate NRR electrocatalysis utilizing the Ti_2_NT_*x*_ MXene for ammonia (NH_3_) synthesis via the Mars-van Krevelen (MvK) mechanism. Isotope-labeled ^15^N_2_ was employed as the reactant in the NRR to probe the role of lattice nitrogen in MXenes. If ^15^N_2_ reduction occurs exclusively at the MXene surface, then the resulting nuclear magnetic resonance (NMR) spectrum should display a doublet corresponding to ^15^NH_3_. However, NMR results revealed the presence of both ^15^NH_3_ and ^14^NH_3_ (Figure 4) when using the ^15^N_2_ feed. Furthermore, the ratio of ^15^NH_3_ to ^14^NH_3_ increased progressively over consecutive NRR cycles with ^15^N_2_ as the reactant. When the same electrode was subsequently exposed to ^14^N_2_, the ratio decreased, yielding predominantly ^14^NH_3_, which eventually became the only product. These isotope experimental results indicate that the NRR on Ti_2_NT_*x*_ MXene proceeds via the MvK mechanism during which the lattice nitrogen first gets protonated to form ^14^NH_3_ and the resulting nitrogen vacancies are then replenished by the ^15^N_2_ reactant, forming ^15^NH_3_ in a subsequent cycle. This demonstrates that NH_3_ production through NRR electrocatalysis involving nitride MXenes originates from lattice nitrogen protonation and subsequent vacancy refilling. In simple terms, an alternative mechanism for NRR electrocatalysis leveraging the lattice nitrogen reactivity of nitride MXene is provided. This approach circumvents the sluggish and energy-intensive dissociation of N_2_ on the catalytic surface. Such knowledge is beneficial since it can be used to greatly facilitate and optimize NRR electrocatalysis involving nitride MXenes.

**Figure 5:**
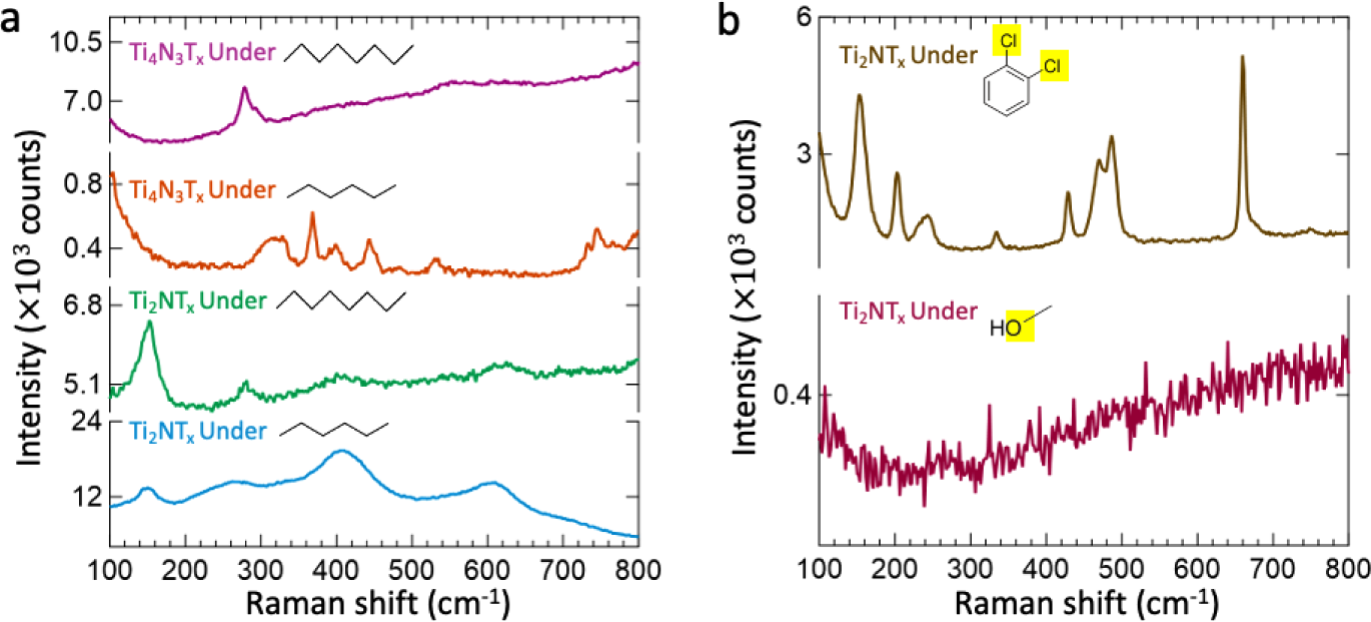
Raman spectra of the a. Ti_2_NT_*x*_ MXene in hydrocarbon solvents, including hexane (sky blue) and in octane (green), Ti_4_N_3_T_*x*_ MXene in hydrocarbon solvents, including hexane (orange) and octane (magenta), and b. Ti_2_NT_*x*_ MXene in solvents in which the hydrocarbon structure is modified, including methanol (magenta) and 1,2-dichlorobenzene (brown). All spectra were collected using the 532 nm laser, 1800 lines/mm grating, and a 50× objective lens. The spectra for the nitride MXenes in hydrocarbon solvents were obtained at 50% laser power. The spectra for Ti_2_NT_*x*_ MXene in methanol and 1,2-dichlorobenzene were obtained at 10% laser power. All of the figures highlight the specificity of the nature of the interaction between the Ti_2_NT_*x*_ MXene and the hydrocarbon solvents to enable the retention of Raman activity. The electronegative atoms of the solvent that can potentially induce the reactivity of the lattice nitrogen of Ti_2_NT_*x*_ MXene to modify the vibrational properties are highlighted in yellow.

We suspect that the presence of species such as oxygen from polar solvents promotes the reactivity of lattice nitrogen, which induces the formation of strong polar bonds. To test this hypothesis, we characterized the nitride MXenes in nonpolar solvents such as hydrocarbons that are absent of such species. The vibrational properties of nitride MXenes are expected to be retained in such environments. Indeed, our results confirm that the vibrational behavior of the Ti_2_NT_*x*_ MXene material remains unmodified through the retention of the Raman scattering process (Figure 5a, sky blue trace and green trace, and Figure S40a). The laser penetrates less deeply through the material in octane compared to in hexane, and the reason for this difference in light refraction is to be determined. Nevertheless, the vibrational behavior induced by hydrocarbon solvents is different from that observed for polar solvents. After this was confirmed, we proceeded to investigate the effect of modifying the hydrocarbon structure of the solvent. We turned to alcohols such as methanol and a nonpolar solvent incorporating electronegative atoms such as 1,2-dichlorobenzene (Figures 5b and S40b). In these solvents, the vibrational behavior of the Ti_2_NT_*x*_ MXene is modified once more, as there are additional species apart from the hydrocarbon structure that can prompt the reactivity of the lattice nitrogen of the Ti_2_NT_*x*_ MXene. There is a clear transition from hydrocarbon solvents, pinpointing the reactivity of the lattice nitrogen in more polar or electronegative solvent environments, resulting in alteration of the vibrational behavior of the N-containing materials.

**Figure 6:**
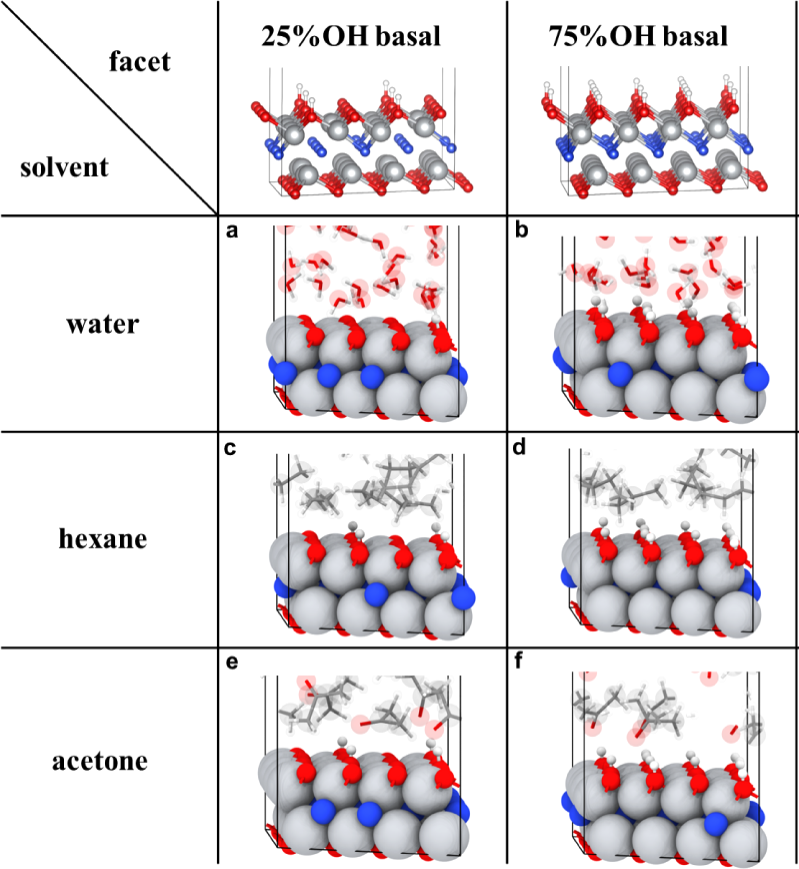
Final configurations of Ti_2_NT_*x*_ surfaces from AIMD simulations in various solvents without hydrogen bonds shown: a. Ti_2_NT_*x*_ basal surface (25% −OH) without water adsorption, some of hydrogen atoms on the −OH terminal group are pulled farther than 1.2 Å (display cutoff between O–H is 1.2 Å), which could be as showed clearer in Figure S44; b. Ti_2_NT_*x*_ basal surface (75% −OH) without water adsorption; c. Ti_2_NT_*x*_ basal surface (25% −OH) showing no solvent adsorption in hexane; d. Ti_2_NT_*x*_ basal surface (75% −OH) showing no solvent adsorption in hexane; e. Ti_2_NT_*x*_ basal surface (25% −OH) showing no acetone adsorption; f. Ti_2_NT_*x*_ basal surface (75% −OH) showing no acetone adsorption. Color code: red, blue, white, gray, and charcoal gray represent O, N, H, Ti, and C atoms.

**Figure 7:**
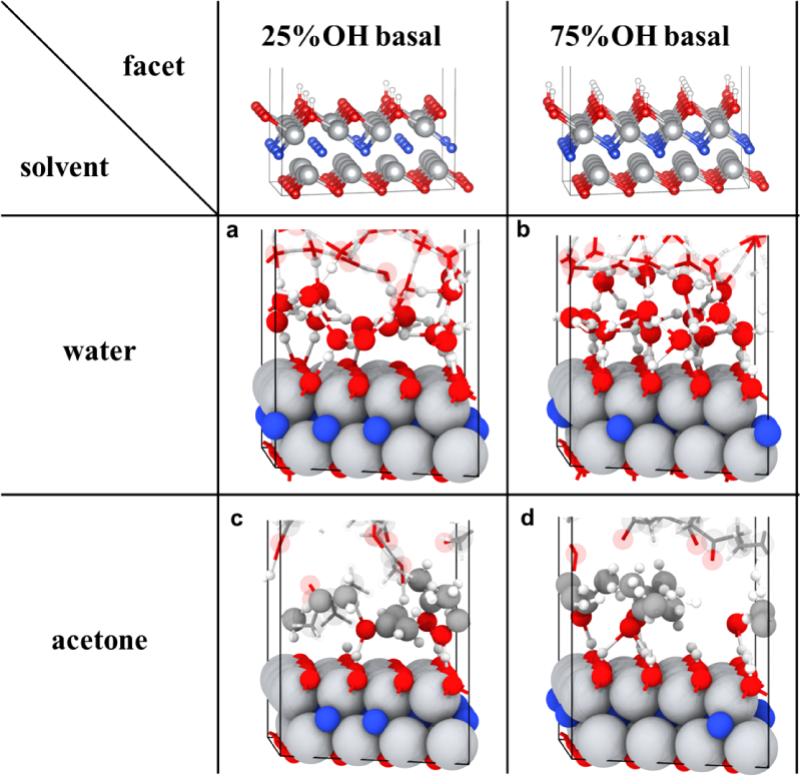
Final configurations of Ti_2_NT_*x*_ surfaces from AIMD simulations in various solvents including hydrogen bonds with a 2.2 Å cutoff: a. Ti_2_NT_*x*_ basal surface (25% −OH) displaying the first-layer hydrogen bonding network; b. Ti_2_NT_*x*_ basal surface (75% −OH) exhibiting distinct and extensive first-layer hydrogen bonding morphology than the 25% −OH Ti_2_NT_*x*_ basal surface; c. Ti_2_NT_*x*_ basal surface (25% −OH) exhibiting limited hydrogen bonding with acetone; d. Ti_2_NT_*x*_ basal surface (75% −OH) displaying extensive hydrogen bonding with acetone. Color code: red, blue, white, gray, charcoal gray, and brown represent O, N, H, Ti, C, and Al atoms.

**Figure 8:**
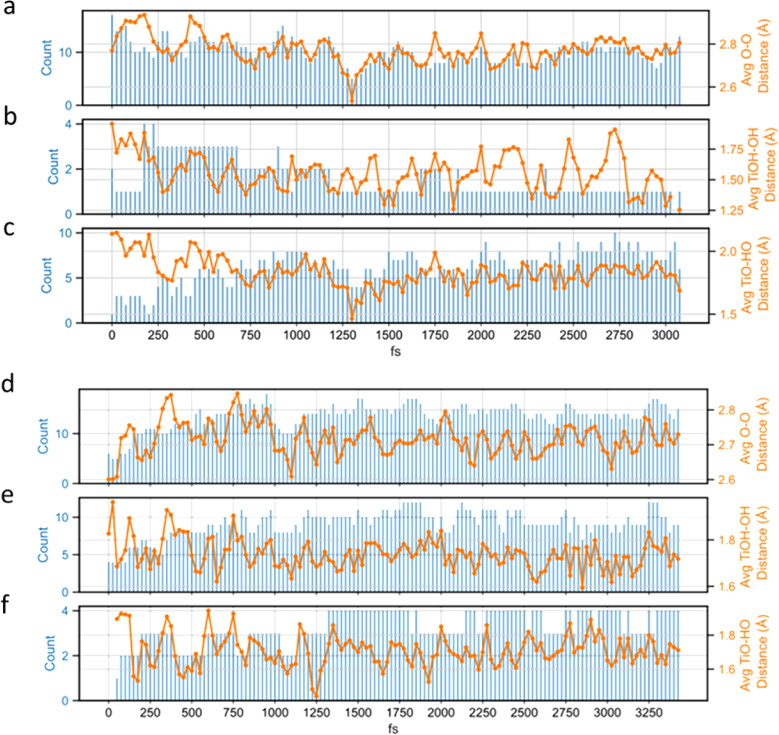
Hydrogen bonding determination of the a–c. Ti_2_NT_*x*_ basal surface (25% −OH coverage) and d–f. Ti_2_NT_*x*_ basal surface (75% −OH coverage) in water via the analysis of the number of hydrogen bonds and average distance of hydrogen bonds between surface and solvent molecules. a and d. O–O pairwise distances between −O– surface terminations of Ti_2_NT_*x*_ and solvent oxygen atoms (cutoff: 3.1 Å). b and e. H–O pairwise distances between hydrogen atoms of −OH surface terminations of Ti_2_NT_*x*_ and oxygen atoms with H-bonds (H–O bond length of <1.2 Å, HB cutoff: 2.2 Å). c and f. O–H pairwise distances between −O– surface terminations of Ti_2_NT_*x*_ and hydrogen atoms bound to oxygen (H–O bond length of <1.2 Å, HB cutoff: 2.2 Å) excluding oxygens that bonded to Ti (Ti–O bond length of <2.6 Å). Noted that bottom fixed surfaces and their terminations were excluded from the analysis.

**Figure 9:**
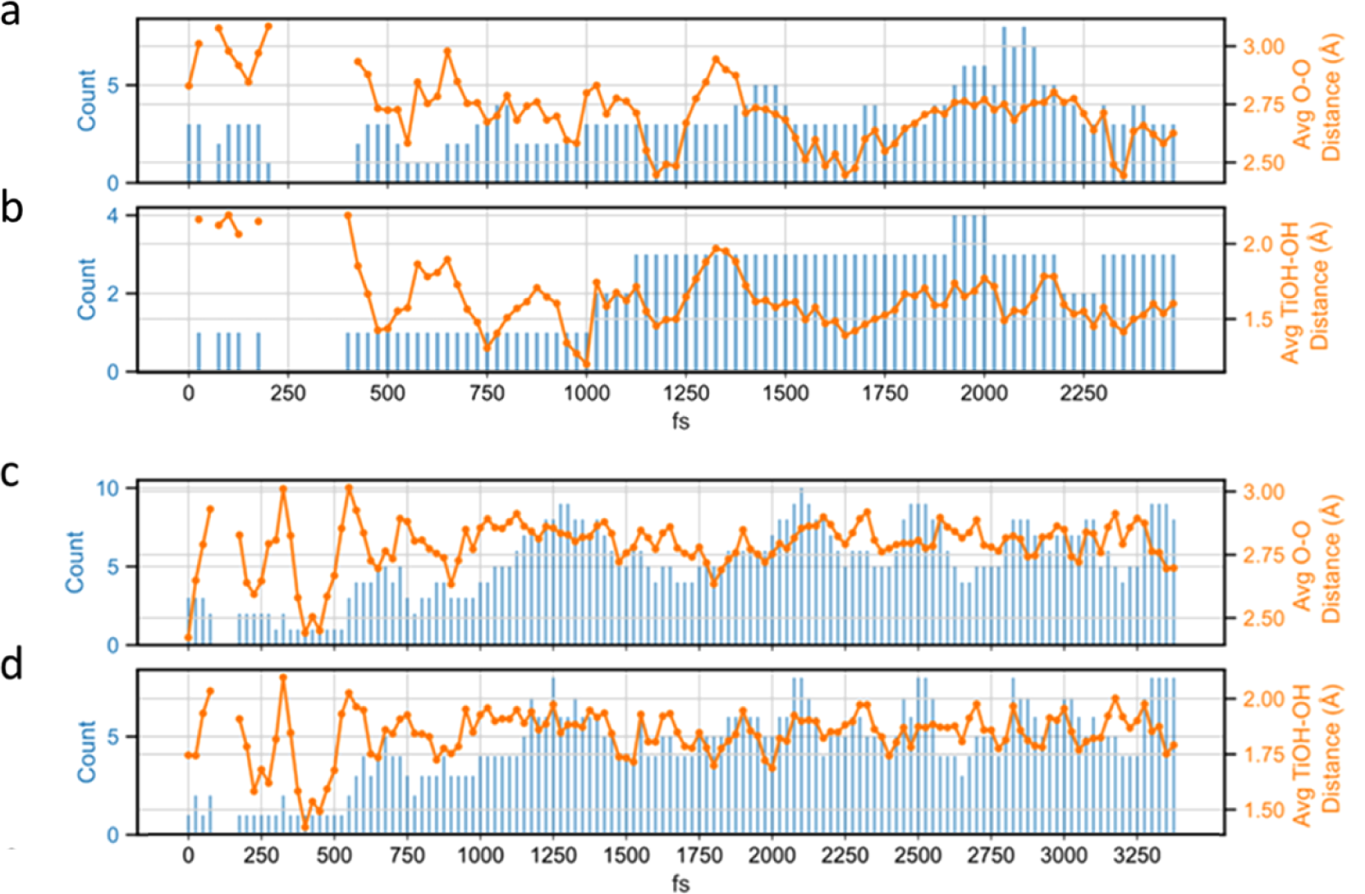
Hydrogen bonding determination of a and b. Ti_2_NT_*x*_ basal surface (25% −OH coverage) and c and d. Ti_2_NT_*x*_ basal surface (75% −OH coverage) in acetone via the analysis of the number of hydrogen bonds and average distance of hydrogen bonding between surface and solvent molecules. a and c. O–O pairwise distances between −O– surface terminations of Ti_2_NT_*x*_ and solvent oxygen atoms (cutoff: 3.1 Å). b and d. H–O pairwise distances between hydrogen atoms of −OH surface terminations of Ti_2_NT_*x*_ and oxygen atoms with H-bonds (H–O bond length of <1.2 Å, HB cutoff: 2.2 Å). Note that bottom fixed surfaces and their terminations were excluded from the analysis.

Having highlighted the reactivity of lattice nitrogen observed in polar solvents, we turned to possible external factors that may also affect the reactivity of nitride MXenes through computational analyses of Ti_2_NT_*x*_ in solvents (Figure 6a–f). Specifically, we focus on those involving hydrogen bonding of a diverse range of termination groups of Ti_2_NT_*x*_ when immersed in polar solvents (Figure 7a–d). When there is a transition from 25% to 75% −OH surface terminations in water, a significant enhancement of hydrogen bonding density involving water and the surface was observed (Figure 8a–f). Also, an accompanying increase in the average bond distance between −OH and oxygen from water (Figure 8b,e) and a slight decrease in the average bond distance between −O– and hydrogen from water can be observed (Figure 8c,f). Compared to the more stable hydrogen bonding nature provided by water, acetone provides a dynamic yet weaker hydrogen bonding network with the axial termination groups due to the absence of a hydrogen bonding acceptor from the oxygen atom of acetone molecules. When there is a transition from 25% to 75% −OH surface terminations in acetone, this increased presence of −OH provides a source for more O atoms of acetone molecules to become involved in hydrogen bonding (Figure 9a–d), with an accompanying increase in the average bond distance between −OH and oxygen from acetone (Figure 9b,d). Assuming that the Ti_2_NT_*x*_ MXene of our work is a mixture of 25% to 75% −OH coverage from previous computational work,^70^ it can be reliably assumed that Ti_2_NT_*x*_ MXene, in water, will exhibit a stable hydrogen bonding network and, in acetone, will exhibit a more dynamic, albeit overall weaker, hydrogen bonding network through its axial termination groups. Such changes or conformation of the hydrogen bonding network can result in implications for the reactivity of Ti_2_NT_*x*_ such as HER applications,^71^ especially when previously reported to directly involve termination groups.^34,51^ However, even with the change in the hydrogen bonding network in different polar solvents or with the role thereof in polar solvents, the alteration of Raman scattering of the Ti_2_NT_*x*_ MXene still remains. Therefore, although there is no direct connection, it appears less likely that the hydrogen bonding in which the termination groups are involved has an influence on the change in the Raman scattering of vibrational properties from the reactivity of the lattice nitrogen with polar solvents. Finally, in all solvents for both the 25% to 75% −OH axial configurations, statistical analysis of Ti–O bond lengths reveals minimal solvent-dependent variations in mean values, with a slightly longer Ti–O bond observed in hexane (Figure S41a,b). Again, assuming a mixture of −OH coverage, the Ti–O bond lengths of Ti_2_NT_*x*_ MXene are expected to be constant in these solvents.

**Figure 10:**
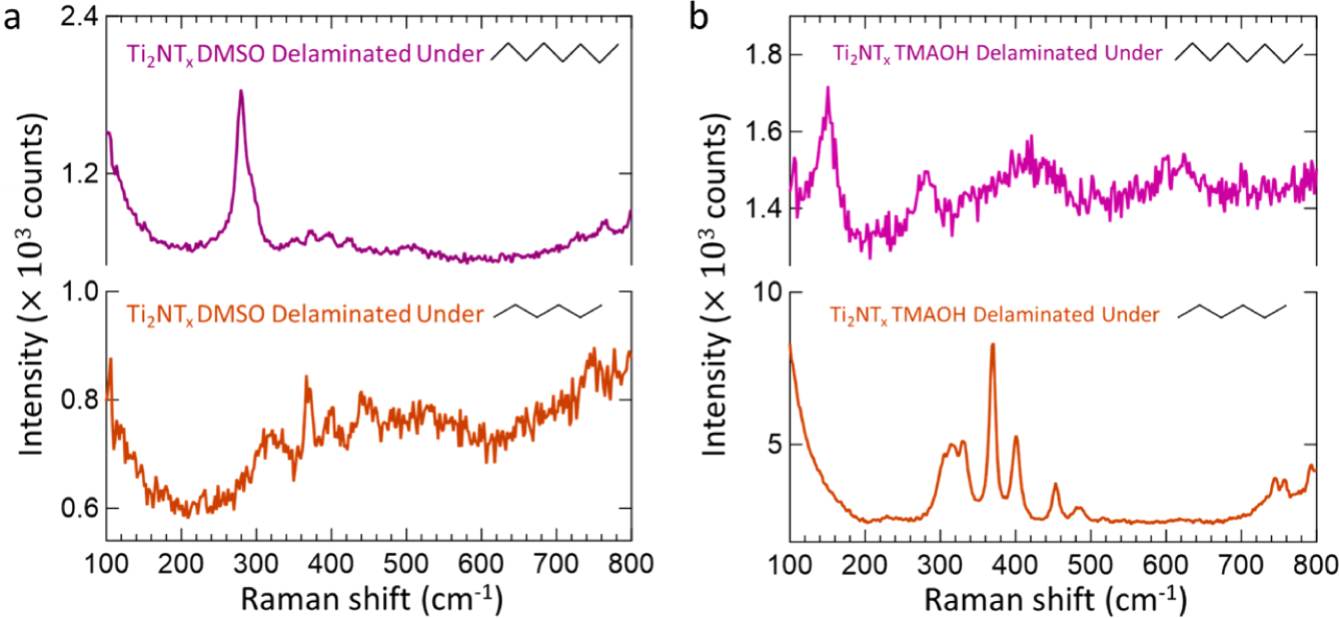
Raman spectra of a. Ti_2_NT_*x*_ MXene delaminated by DMSO and b. Ti_2_NT_*x*_ MXene delaminated by TMAOH in hexane (orange) and octane (magenta). All spectra were collected using the 532 nm laser, 1800 lines/mm grating, and a 50× objective lens. The spectra were obtained at 50% laser power. All of the figures highlight the specificity of the nature of the interaction between the Ti_2_NT_*x*_ MXene and the hydrocarbon solvents to enable retention of Raman activity.

However, additional outside layers of complexity related to the modification of the vibrational behavior of the nitride MXenes in solvents are introduced. Up until this point, we have primarily studied Ti_2_NT_*x*_ MXenes delaminated using water, a foundational system that shows the transition of the reactivity from polar, electronegative solvents to hydrocarbon solvents. Given the chemistry of hydrocarbon solvents, we expect that the vibrational behavior of nitride MXenes will remain unmodified in hydrocarbon solvents. Unexpectedly yet interestingly, when the Ti_2_NT_*x*_ MXene is delaminated using DMSO, its vibrational behavior becomes altered in hydrocarbon solvents, as indicated by the disappearance of the MXene vibrational modes (Figures 10a and S40a). While preliminary, this suggests that external factors may also influence the vibrational behavior in solvents, such as specific structural intricacies of Ti_2_NT_*x*_ MXene induced by a different delaminating agent. Further, we observe that when the Ti_2_NT_*x*_ MXene is delaminated using TMAOH, the vibrational properties are altered in hexane while they are retained in octane (Figure 10b). By introducing a transition point between the different delamination methods of the Ti_2_NT_*x*_ MXene, this further elaborates on how the resulting structural intricacies of the Ti_2_NT_*x*_ MXene influence the vibrational properties in hydrocarbon solvents, which is of interest for further investigation in future works. Unlike the Ti_2_NT_*x*_ MXene, the Ti_4_N_3_T_*x*_ MXene exhibits modified vibrational behavior in the same hydrocarbon solvents (Figure 10a, orange and magenta traces, and Figure S40a). This introduces another layer of complexity as the number of atomic layers of nitride MXenes also influences the modification of vibrational behavior in solvents. While the reactivity of lattice nitrogen provides important insights, additional questions about the specific structural and compositional factors contributing to the modification are raised. They must be further investigated in order to gain an accurate understanding of the MXenes under electrocatalytic conditions to enable proper optimizations.

## Conclusion

While nitride MXenes are promising electrocatalysts for sustainable energy applications, a fundamental understanding of their vibrational behavior in electrolytic solvents remains lacking. This understanding is crucial for effective optimization, especially considering the reactivity of lattice nitrogen with solvents that can impact the overall electrocatalytic performance of the MXenes. Our work addresses this gap by providing an extensive Raman characterization of MXene materials immersed in polar and nonpolar solvents, revealing the underlying modified vibrational behavior of nitride MXenes from the alteration of the Raman scattering process. We successfully establish the presence of reactivity of the lattice nitrogen atom in solvents through a comparative Raman analysis of TiN, AlN, and Ti_2_AlN MAX in water, showing the effect of the material coordination environment on the Raman scattering. This conclusion is also supported by an additional comparison between carbide, carbonitride, and nitride MXenes in solvents. Further corroboration is provided through the study of the Ti_2_NT_*x*_ MXene in hydrocarbon solvents, in which the vibrational behavior remains unmodified. Altogether, these findings provide an opportunity to tune certain vibrational properties for improved electrocatalytic performance through modification of the X atom.

## Supporting Information Available

XRD of Ti_2_AlN MAX, Ti_2_NT_*x*_ MXene, Ti_4_AlN_3_ MAX, and Ti_4_N_3_T_*x*_ MXene; TEM images of Ti_2_NT_*x*_ MXene; Raman spectra of pure-phase polar solvents; depth acquisition and acquisition time experiment of Ti_2_NT_*x*_ underneath a water droplet; Raman spectra of thin layer and powder Ti_2_NT_*x*_ on a Si wafer when fresh and when immersed in acetone; Raman spectra of different spots of the Ti_2_NT_*x*_ material almost completely dried from immersion in DMSO; Raman spectra of TiN, Ti_2_AlN MAX, and AlN fresh and in water; AIMD simulations of the AlN (001) surface in water; Raman spectra of the Ti_2_NT_*x*_ material exposed with minimal water layers; Raman spectra of Ti_2_NT_*x*_, Ti_4_N_3_T_*x*_, and Ti_3_CNT_*x*_ when fresh, when immersed in polar solvents, and when dried using different laser powers and laser wavelengths; Raman spectra of bulk transition metal nitrides and corresponding oxides in water; XPS analysis of N-doped Ti_3_C_2_T_*x*_ MXene materials; Raman spectra of N-doped Ti_3_C_2_T_*x*_ MXene materials; Raman spectra of pure-phase nonpolar hydrocarbon solvents, methanol, and 1,2-dichlorobenzene; bond distance analyses of Ti–N and Ti–O for Ti_2_NT_*x*_ surfaces in various solvents (PDF)
