# Supplementary material for: Vibrational Property Tuning of MXenes Revealed by Sublattice N Reactivity in Polar and Nonpolar Solvents
Source: J Am Chem Soc. 2025 Feb 4;147(12):10104–17. doi: 10.1021/jacs.4c13878 (PMC11951077; doi:10.1021/jacs.4c13878)
Supplement: Supplementary file 1 — ja4c13878_si_001.pdf [file ja4c13878_si_001.pdf]

Supporting Information

Vibrational Property Tuning of MXenes Revealed by Sublattice N Reactivity in Polar and Non-Polar Solvents

Ray M. S. Yoo[1], Bright Ngozichukwu[1], David Kumar Yesudoss[1], Hao-En Lai[1], Kailash Arole[1,2], Micah J. Green[1,2], Perla B. Balbuena[1,2,3], Abdoulaye Djire[1,2]*

[1]Artie McFerrin Department of Chemical Engineering, Texas A&M University, College Station, TX 77843, USA

[2]Department of Materials Science & Engineering, Texas A&M University, College Station, TX 77843, USA

[3]Department of Chemistry, Texas A&M University, College Station, TX 77843, USA

*Corresponding Author: adjire@tamu.edu

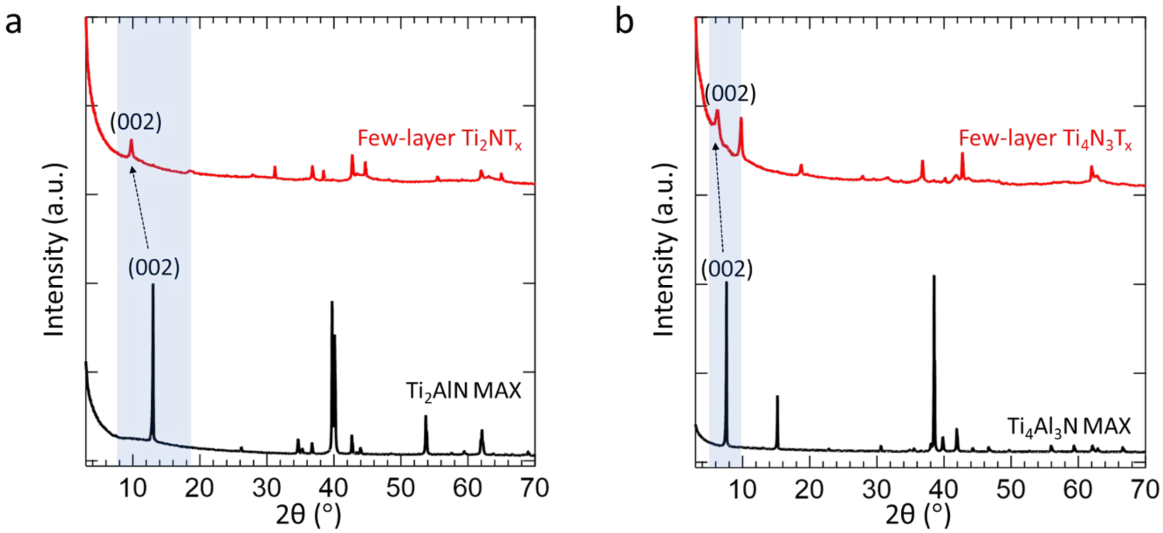

Figure S1. XRD characterization of **a.** Ti_2AlN starting MAX phase and few-layer Ti_2NT_x MXene and **b.** Ti_4Al_3N starting MAX phase and few-layer $Ti_4N_3T_x$ MXene. The XRD data suggest the

successful synthesis of the Ti-based nitride MXenes using the oxygen-assisted molten salt fluoride method.

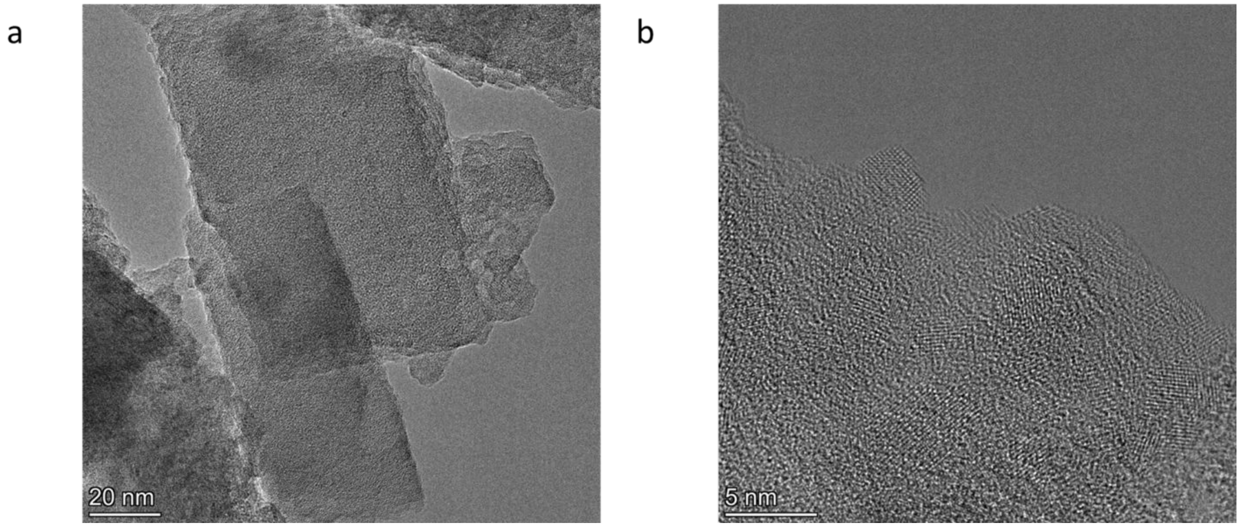

Figure S2. a. TEM (20 nm scale) and **b.** High resolution TEM (5 nm scale) characterization of few-layer Ti_2NT_x MXene.

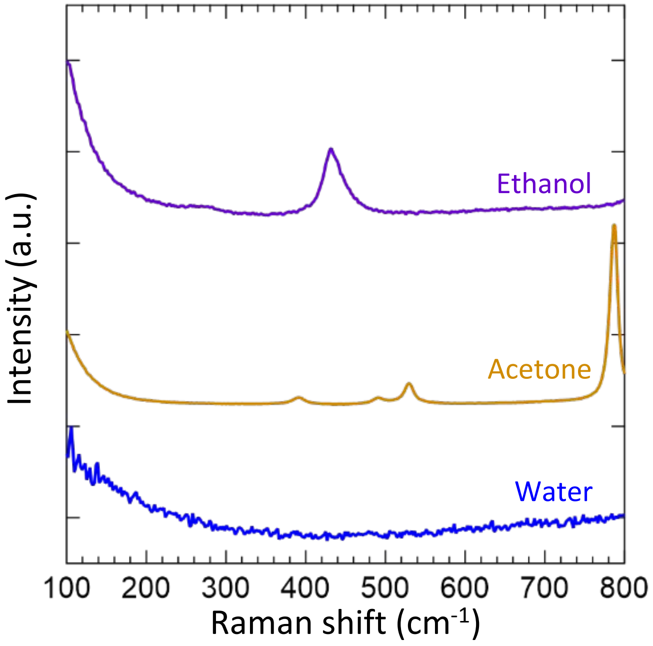

Figure S3. Raman spectra of the following polar solvents used in the study: water (blue), acetone (gold), and ethanol (purple). All spectra were obtained at 100% laser power. Spectra were collected using a 532 nm laser, 1800 lines/mm grating, and 50x objective lens.

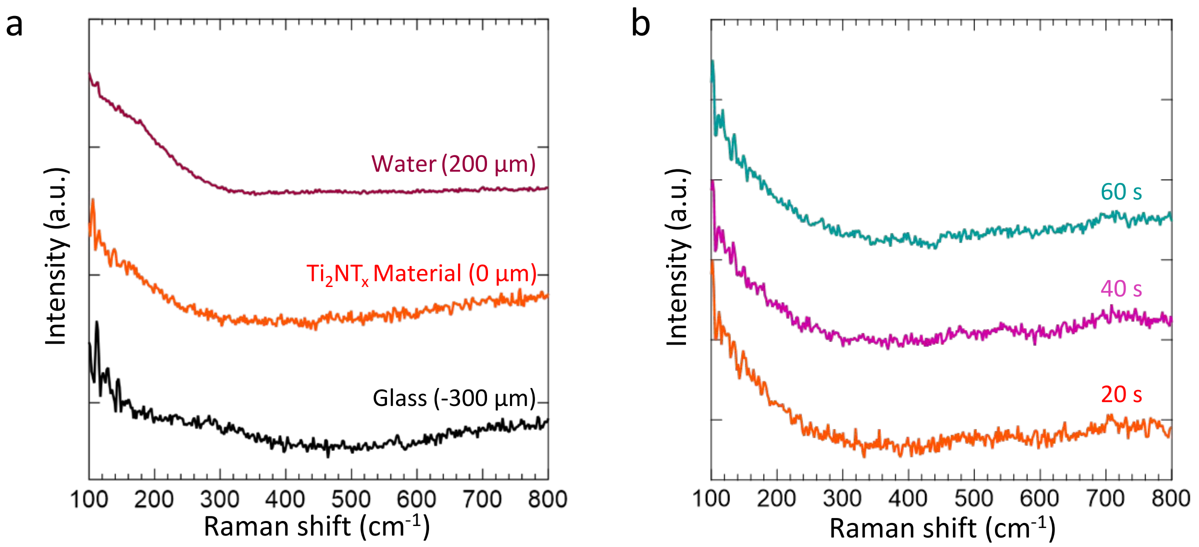

Figure S4. a. Depth acquisition experiment and **b.** acquisition time experiment of the Ti_2NT_x MXene with water droplet on top. For the depth acquisition experiment, water and glass spectra are provided as reference collected from a depth acquisition experiment spanning z-axis locations -300 µm to 200 µm, where Ti_2NT_x MXene surface was set to z-axis location of 0 µm. All the spectra for the acquisition time experiment were collected on the Ti_2NT_x MXene surface. These data show that the material is clearly probed and that the instrumental parameters and experimental setup are irrelevant to the observed shift in Raman activity of the Ti_2NT_x MXene. All spectra were obtained at 50% or 100% laser power to access the MXene subsurface structure. Spectra were collected using a 532 nm laser, 1800 lines/mm grating, and 50x objective lens.

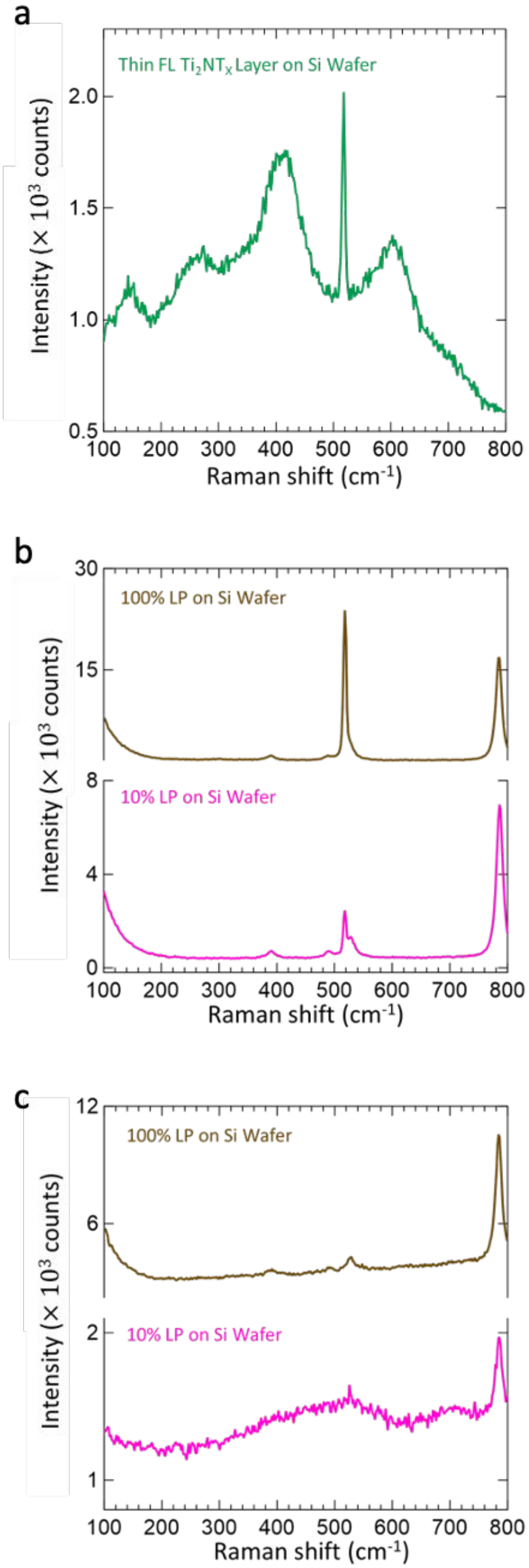
a
Thin FL Ti2NTX Layer on Si Wafer
Intensity (× 10^3 counts)
Raman shift (cm^-1)
b
100% LP on Si Wafer
10% LP on Si Wafer
Intensity (× 10^3 counts)
Raman shift (cm^-1)
c
100% LP on Si Wafer
10% LP on Si Wafer
Intensity (× 10^3 counts)
Raman shift (cm^-1)

Figure S5. a. Raman spectra of thin layer of dry Ti_2NT_x MXene on Si wafer (green), demonstrating it is Raman active at this thickness. **b.** Raman spectra of background acetone spectrum and thin layer of Ti_2NT_x MXene on Si wafer under acetone at 10% laser power (LP) (pink) and 100% laser power (LP) (brown). **c.** Raman spectra of background acetone spectrum and Ti_2NT_x MXene powder on Si wafer under acetone at 10% LP (pink) and 100% LP (brown), showing the absence of the Si wafer characteristic peak due to increased thickness of sample. These data serve to show the shift in Raman activity of Ti_2NT_x MXene under solvents that leads to a different spectrum which accounts for the Raman activity of whatever else in question. Spectra were collected using a 532 nm laser, 1800 lines/mm grating, and 50x objective lens.

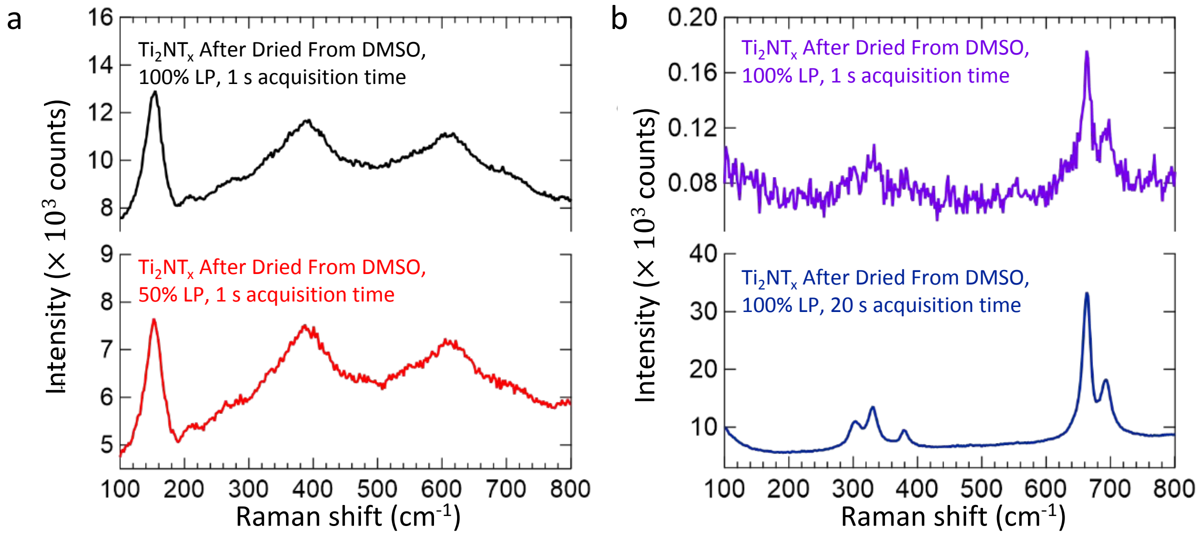

Figure S6. Raman characterization of different spots of Ti_2NT_x MXene material almost completely dried from immersion under DMSO obtained, with spectra of **a.** dried spots and **b.** undried spots. All spectra were obtained at 100% laser power (LP). All spectra were collected using the 532 nm laser, 1800 lines/mm grating, and 50x objective lens. All of the figures corroborate the alteration in the Raman scattering process when the Ti_2NT_x MXene is immersed under polar solvents.

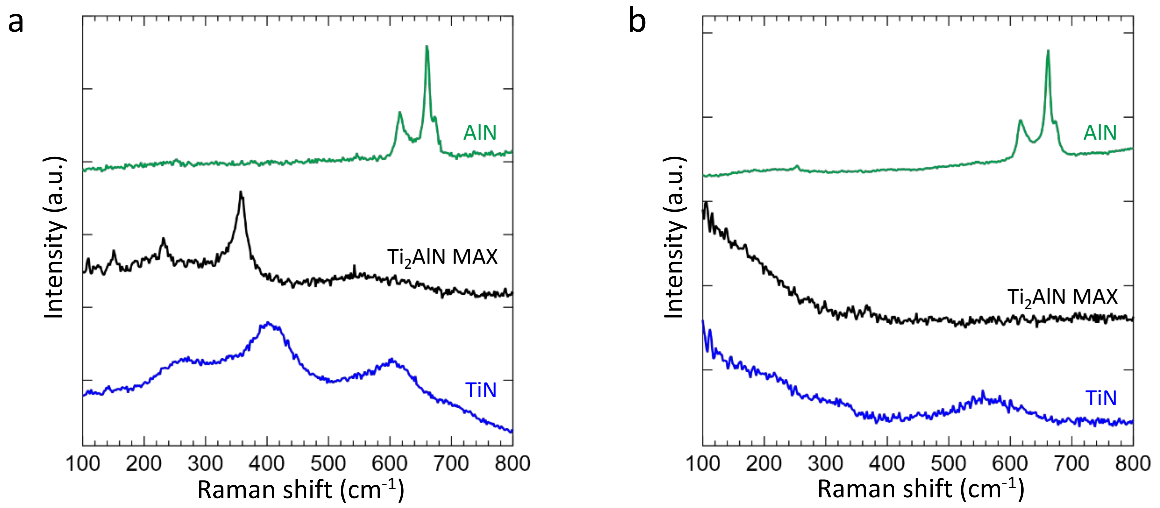

Figure S7. Raman spectra of **a.** bulk nitride materials TiN (blue), Ti_2AlN MAX (black), and AlN (green) and **b.** same nitride materials under water, where only the AlN retains the Raman scattering properties. Spectra were collected at 10% laser power for comparison. Spectra were collected using a 532 nm laser, 1800 lines/mm grating, and 50x objective lens. The data aims to show initial indication of the reactivity of the lattice nitrogen of nitride materials towards solvents, which is affected by the coordination environment of the metal nitride.

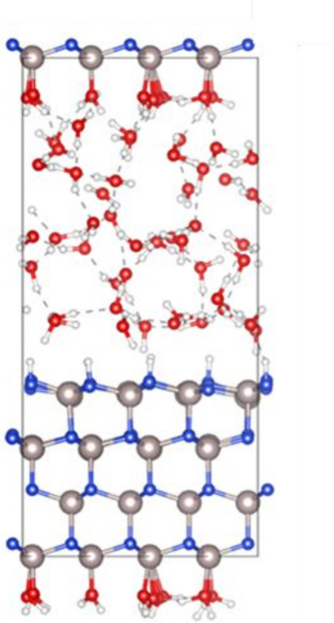

Figure S8. Computational configuration of AlN (001) surface in water from AIMD simulations. Extensive Al-OH and N-H bond formation was observed following water splitting on AlN (001) surface. On AlN (001), Al-OH bonds formed exclusively on the alumina-exposed surface of fixed upper facets, while N-H bonds formed on the opposite side through proton transfer. Color code: red, blue, white, charcoal grey and brown represent O, N, H, C and Al atoms.

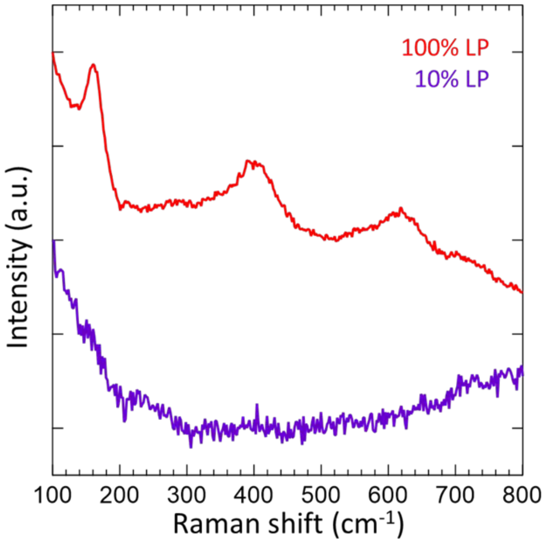

Figure S9. Raman spectra of Ti_2NT_x MXene material exposed with minimal water layer at the surface at 10% LP (purple) and 100% LP (red). The Ti_2NT_x MXene material is exposed to a droplet of water and then this droplet is extracted until minimal water is left, which the MXene material is left to dry for a little time. All spectra were collected using the 532 nm laser, 1800 lines/mm grating, and 50x objective lens. The spectra obtained at 10% LP probes the surface of the MXene that has contact and exposure to the water while that obtained at 100% LP probes the subsurface that has its dryness retained.

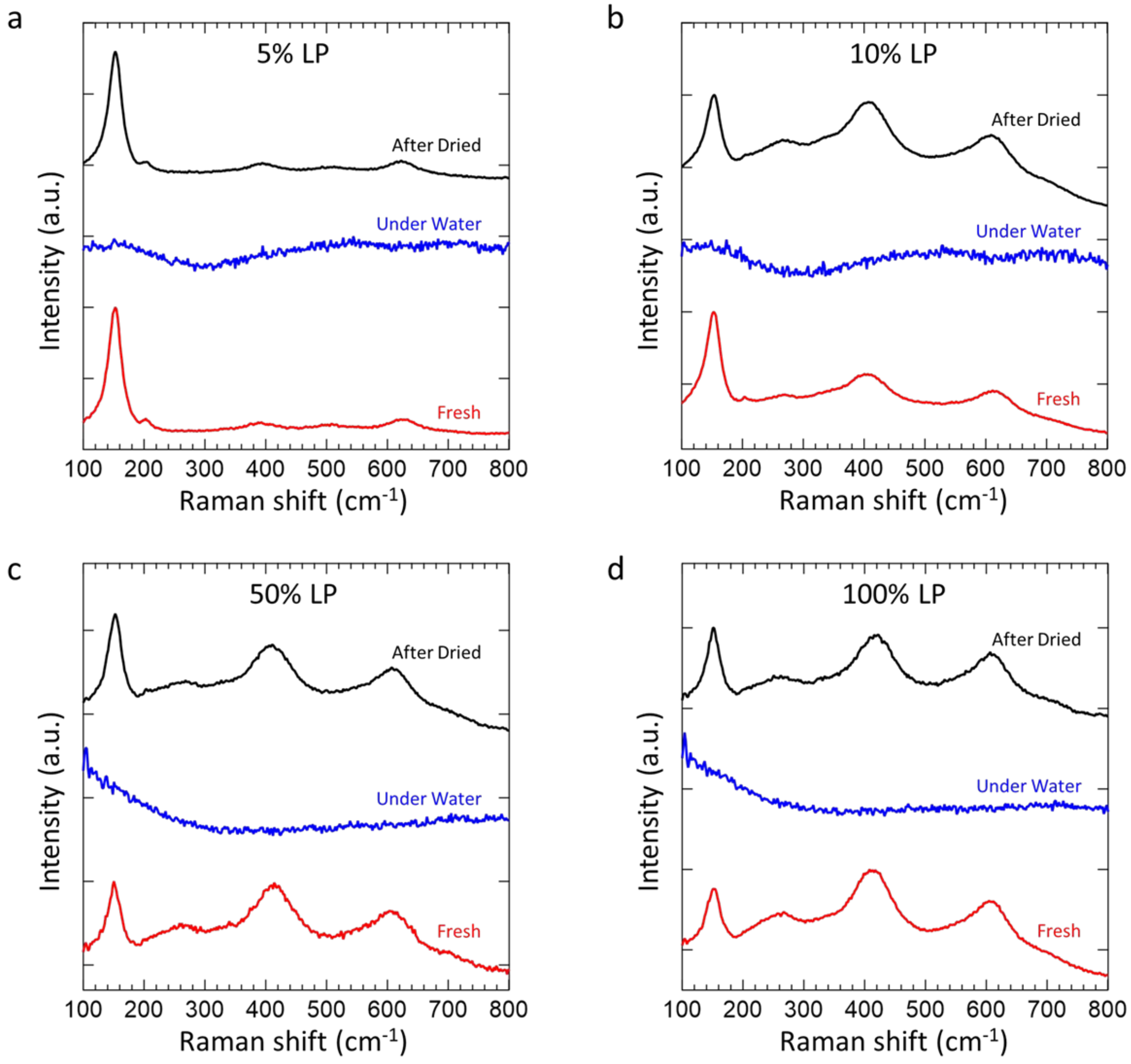

Figure S10. Raman spectra of Ti_2NT_x MXene that is fresh (red), under water (blue), and after dried (black) obtained with 532 nm laser at **a.** 5% LP, **b.** 10% LP, **c.** 50% LP, and **d.** 100% LP. All spectra were collected using the 532 nm laser, 1800 lines/mm grating, and 50x objective lens.

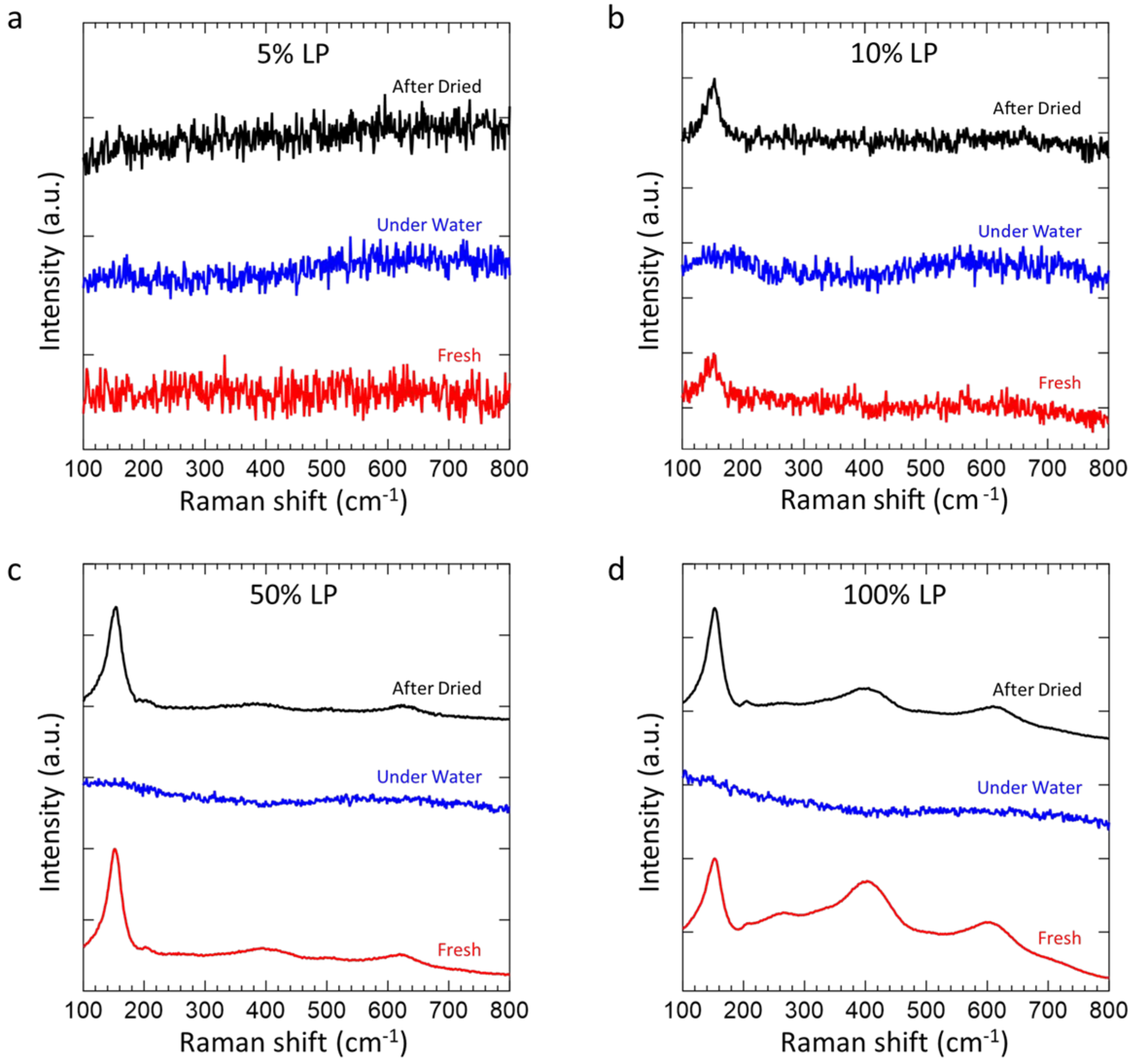

Figure S11. Raman spectra of Ti_2NT_x MXene that is fresh (red), under water (blue), and after dried (black) obtained with 633 nm laser at **a.** 5% LP, **b.** 10% LP, **c.** 50% LP, and **d.** 100% LP. All spectra were collected using the 633 nm laser, 1800 lines/mm grating, and 50x objective lens.

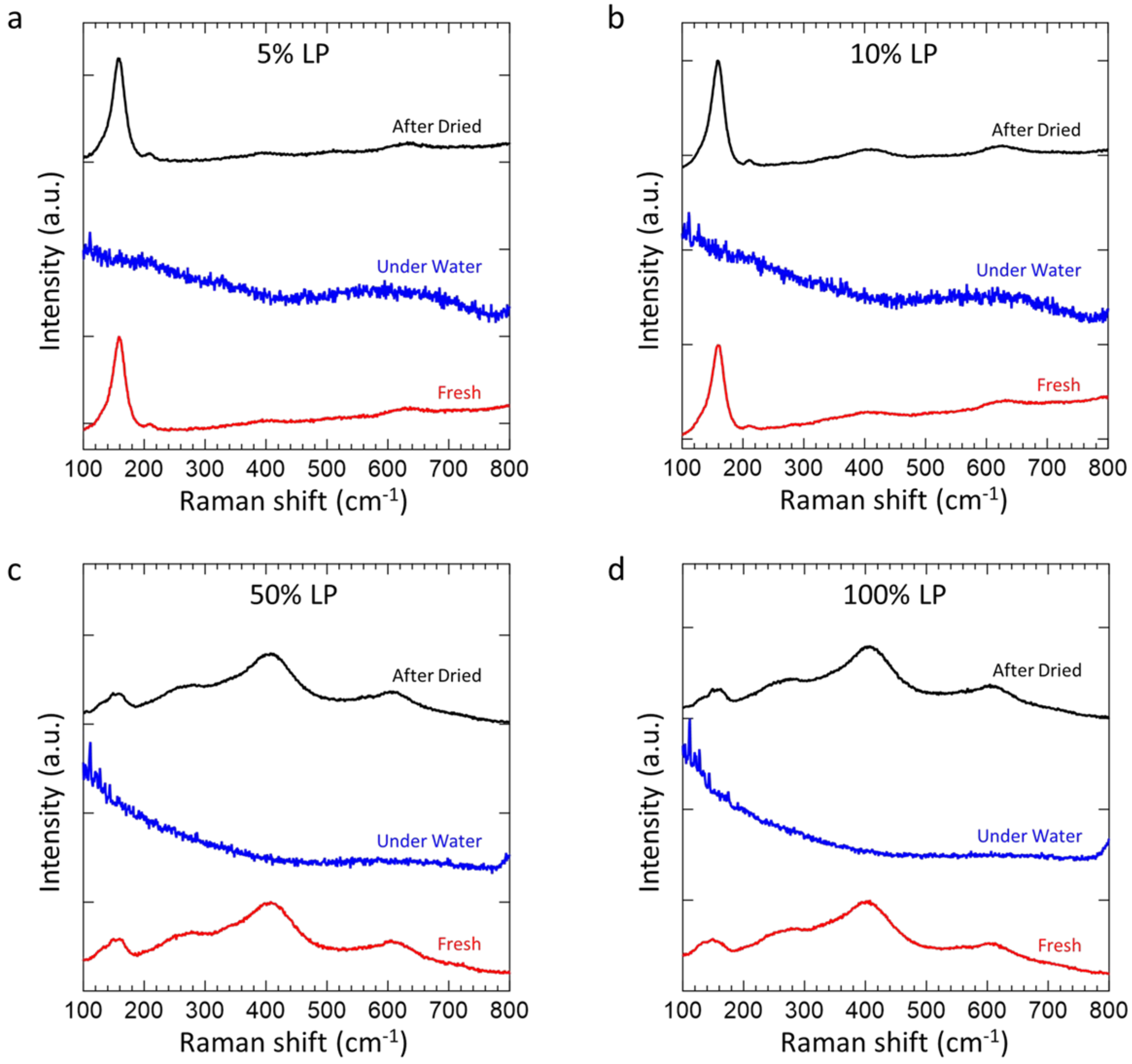

Figure S12. Raman spectra of Ti_2NT_x MXene that is fresh (red), under water (blue), and after dried (black) obtained with 785 nm laser at **a.** 5% LP, **b.** 10% LP, **c.** 50% LP, and **d.** 100% LP. All spectra were collected using the 785 nm laser, 1800 lines/mm grating, and 50x objective lens.

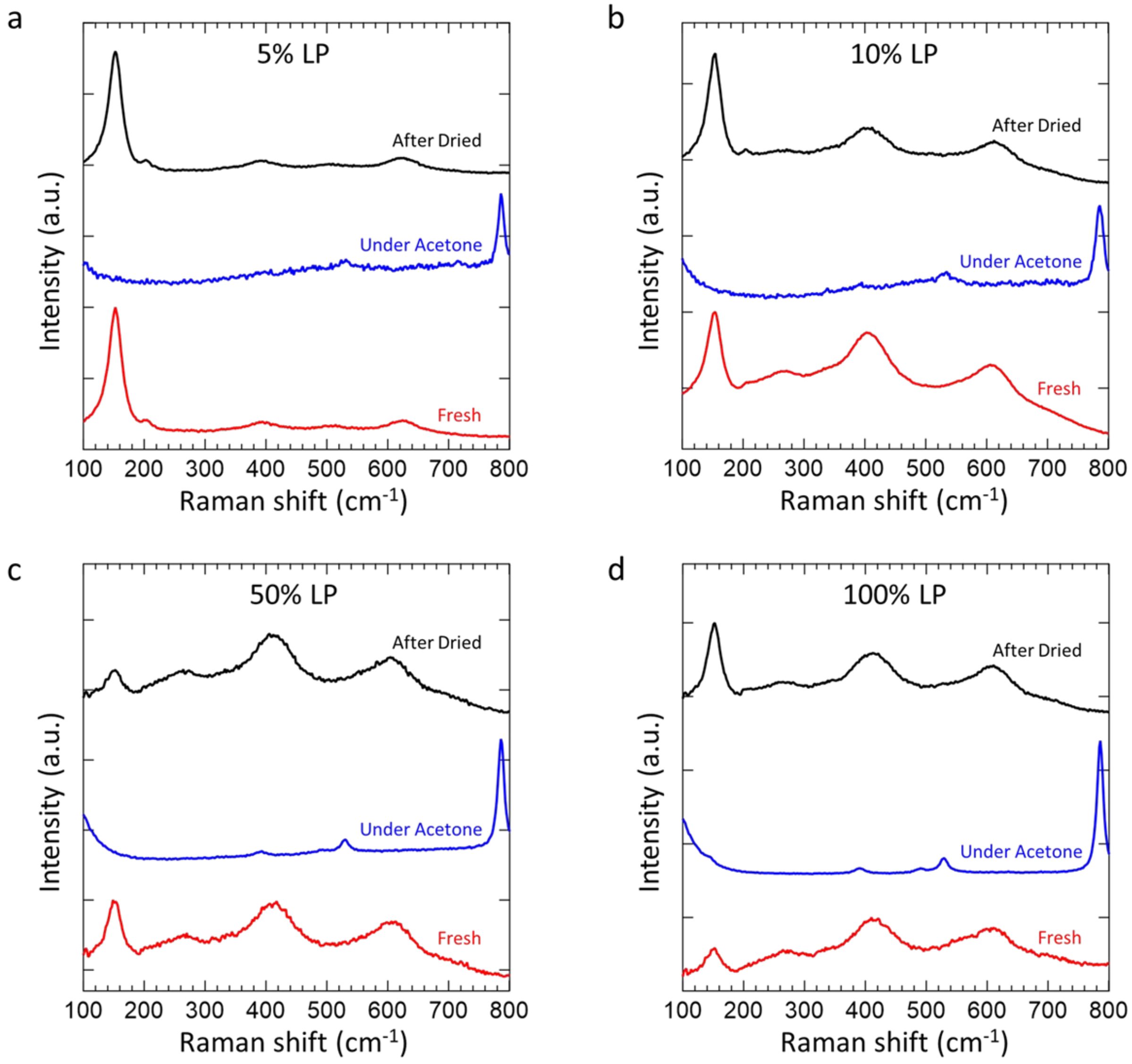

Figure S13. Raman spectra of Ti_2NT_x MXene that is fresh (red), under acetone (blue), and after dried (black) obtained with 532 nm laser at **a.** 5% LP, **b.** 10% LP, **c.** 50% LP, and **d.** 100% LP. All spectra were collected using the 532 nm laser, 1800 lines/mm grating, and 50x objective lens.

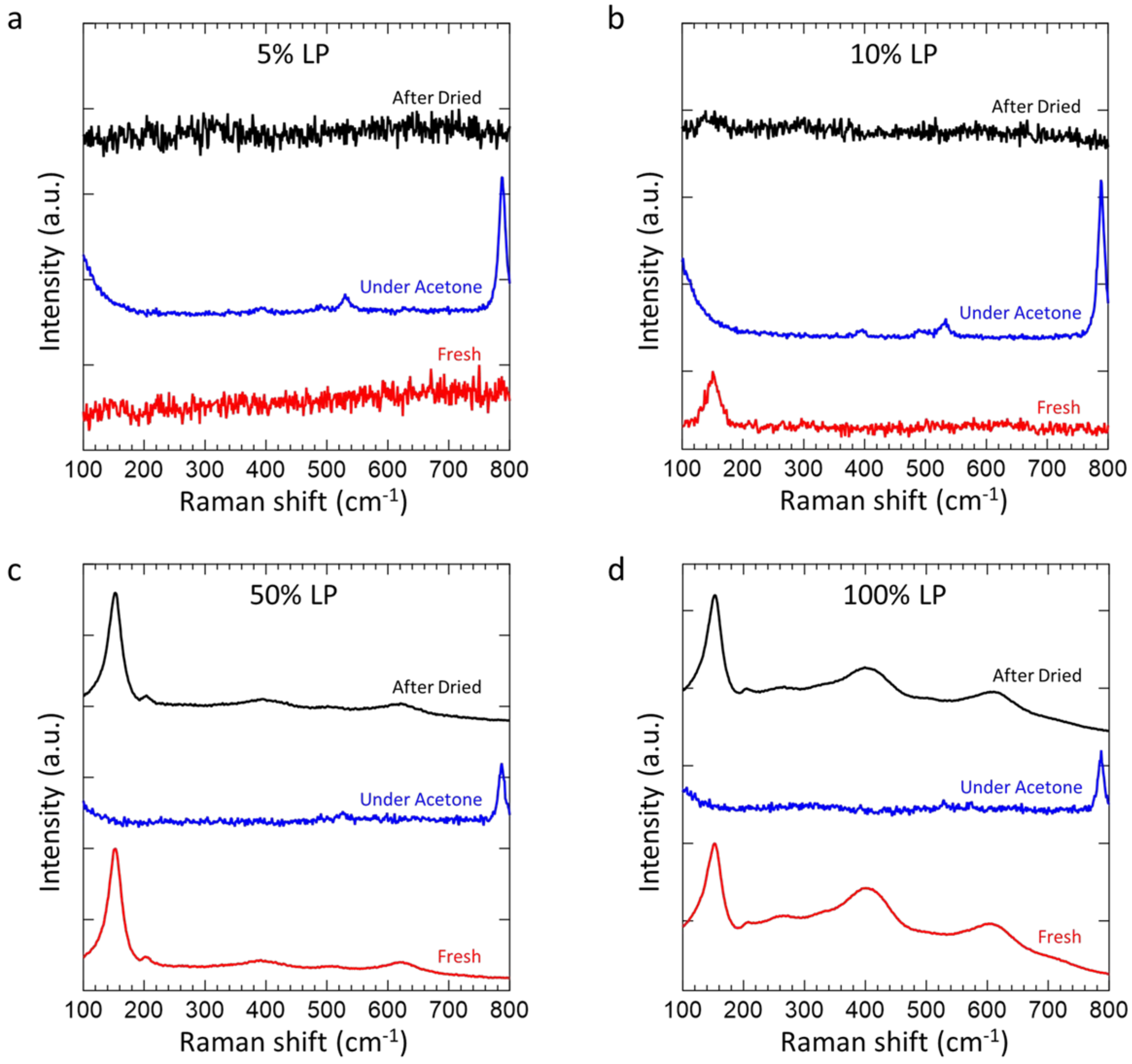

Figure S14. Raman spectra of Ti_2NT_x MXene that is fresh (red), under acetone (blue), and after dried (black) obtained with 633 nm laser at **a.** 5% LP, **b.** 10% LP, **c.** 50% LP, and **d.** 100% LP. All spectra were collected using the 633 nm laser, 1800 lines/mm grating, and 50x objective lens.

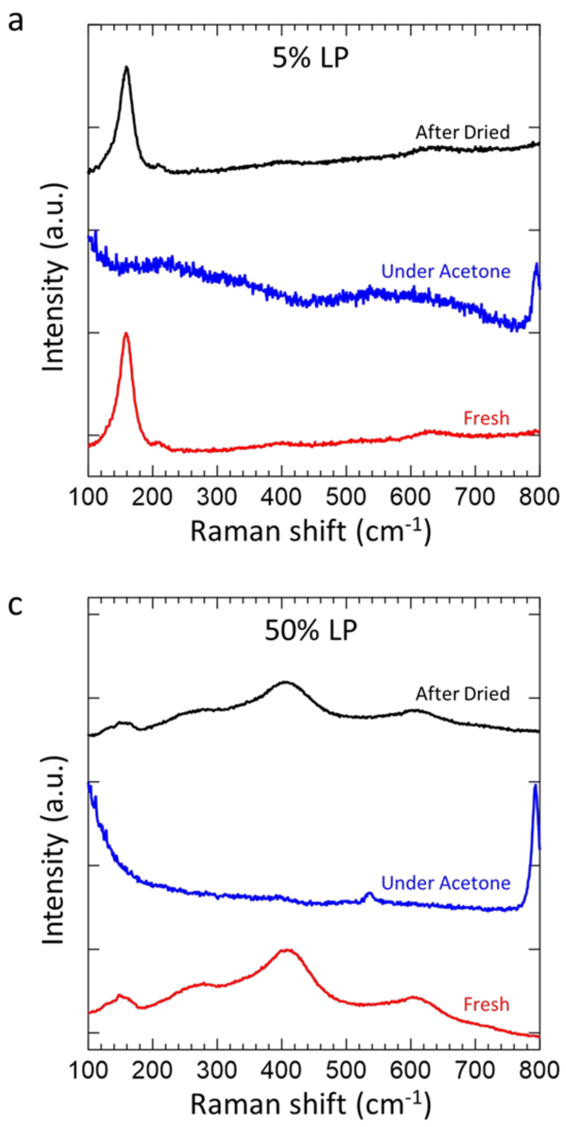

Figure S15. Raman spectra of Ti_2NT_x MXene that is fresh (red), under acetone (blue), and after dried (black) obtained with 785 nm laser at **a.** 5% LP, **b.** 10% LP, **c.** 50% LP, and **d.** 100% LP. All spectra were collected using the 785 nm laser, 1800 lines/mm grating, and 50x objective lens.

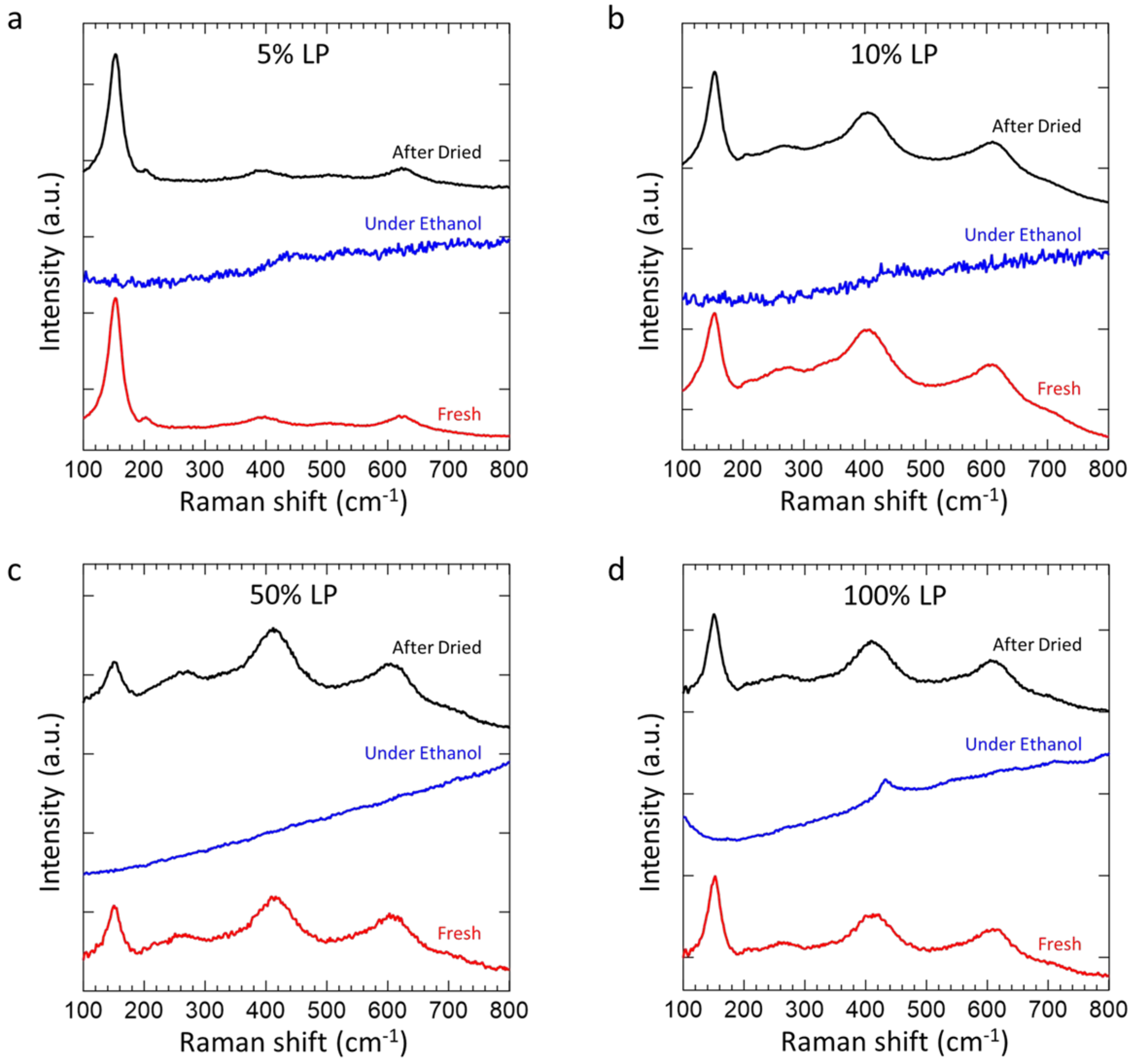

Figure S16. Raman spectra of Ti_2NT_x MXene that is fresh (red), under ethanol (blue), and after dried (black) obtained with 532 nm laser at **a.** 5% LP, **b.** 10% LP, **c.** 50% LP, and **d.** 100% LP. All spectra were collected using the 532 nm laser, 1800 lines/mm grating, and 50x objective lens.

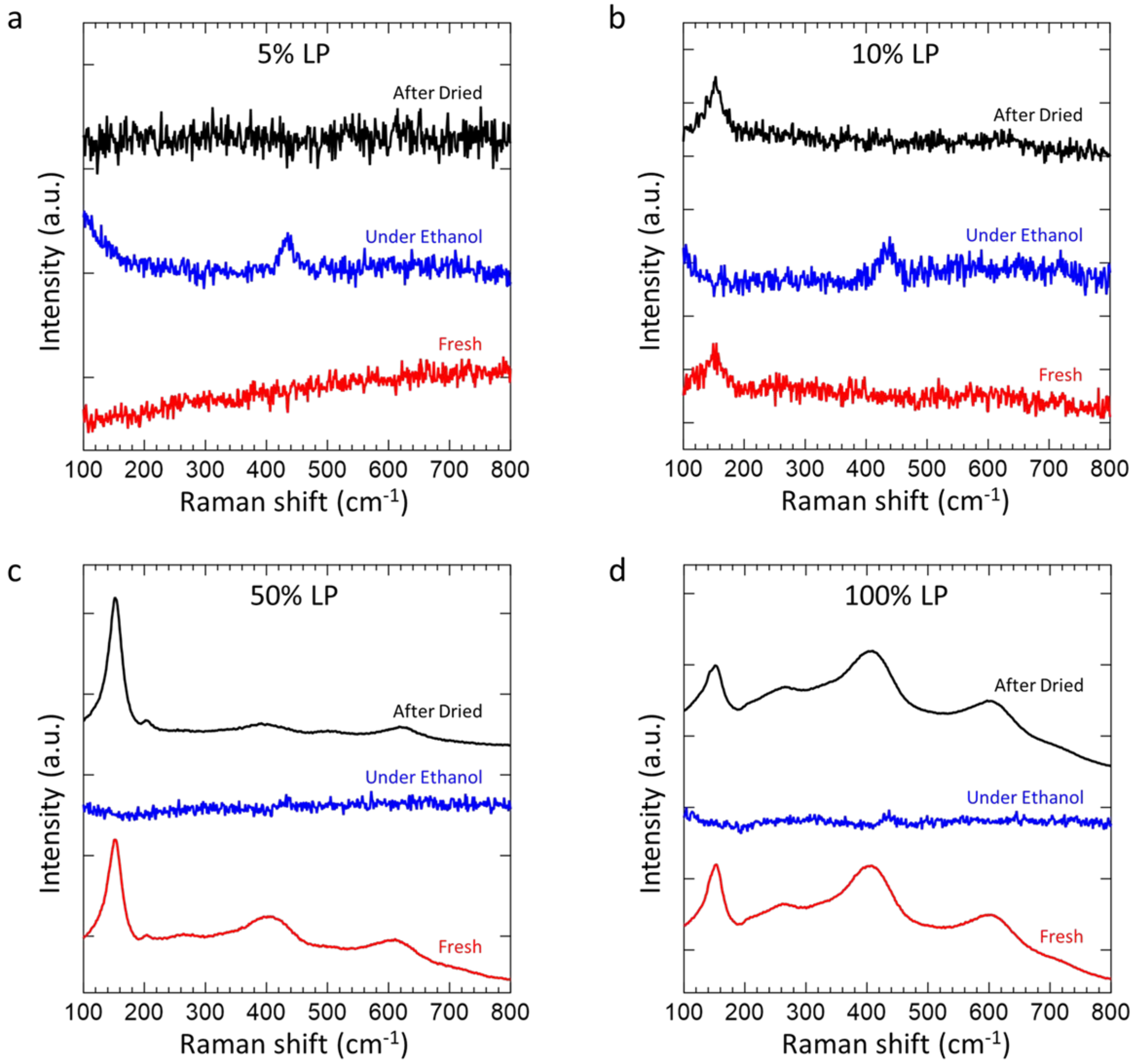

Figure S17. Raman spectra of Ti_2NT_x MXene that is fresh (red), under ethanol (blue), and after dried (black) obtained with 633 nm laser at **a.** 5% LP, **b.** 10% LP, **c.** 50% LP, and **d.** 100% LP. All spectra were collected using the 633 nm laser, 1800 lines/mm grating, and 50x objective lens.

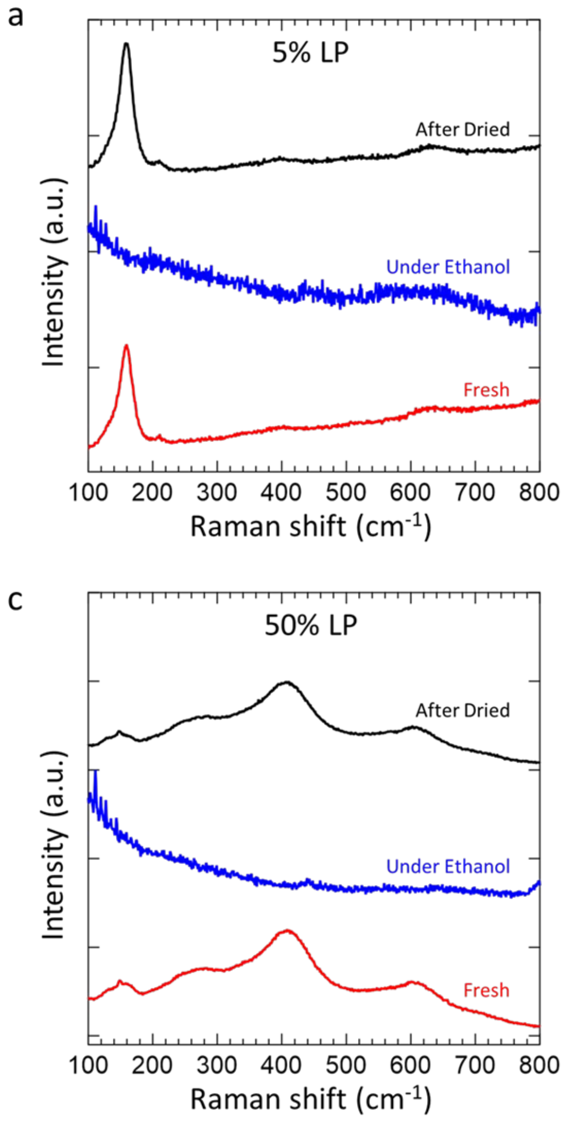

Figure S18. Raman spectra of Ti_2NT_x MXene that is fresh (red), under ethanol (blue), and after dried (black) obtained with 785 nm laser at **a.** 5% LP, **b.** 10% LP, **c.** 50% LP, and **d.** 100% LP. All spectra were collected using the 785 nm laser, 1800 lines/mm grating, and 50x objective lens.

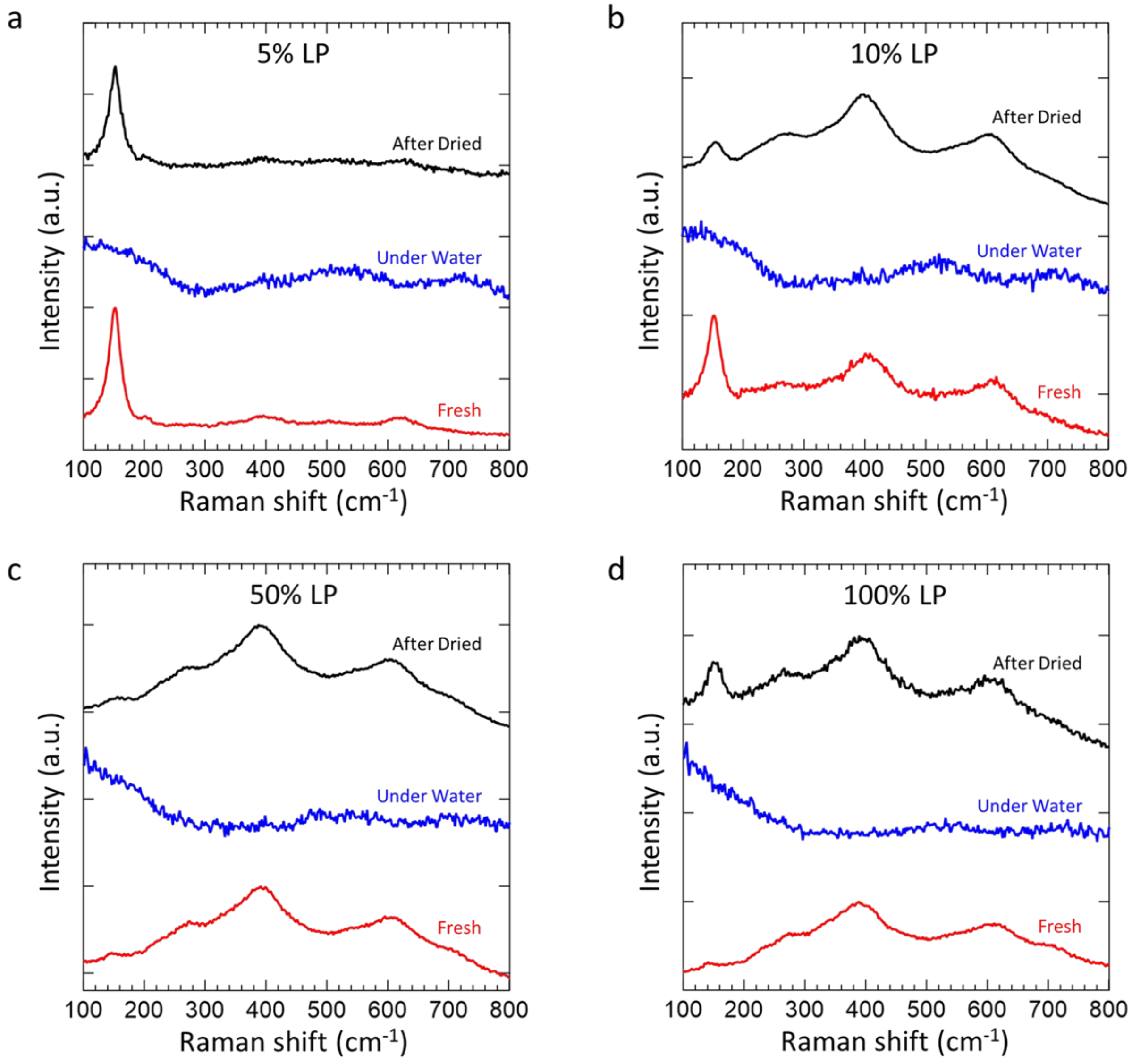

Figure S19. Raman spectra of $Ti_4N_3T_x$ MXene that is fresh (red), under water (blue), and after dried (black) obtained with 532 nm laser at **a.** 5% LP, **b.** 10% LP, **c.** 50% LP, and **d.** 100% LP. All spectra were collected using the 532 nm laser, 1800 lines/mm grating, and 50x objective lens.

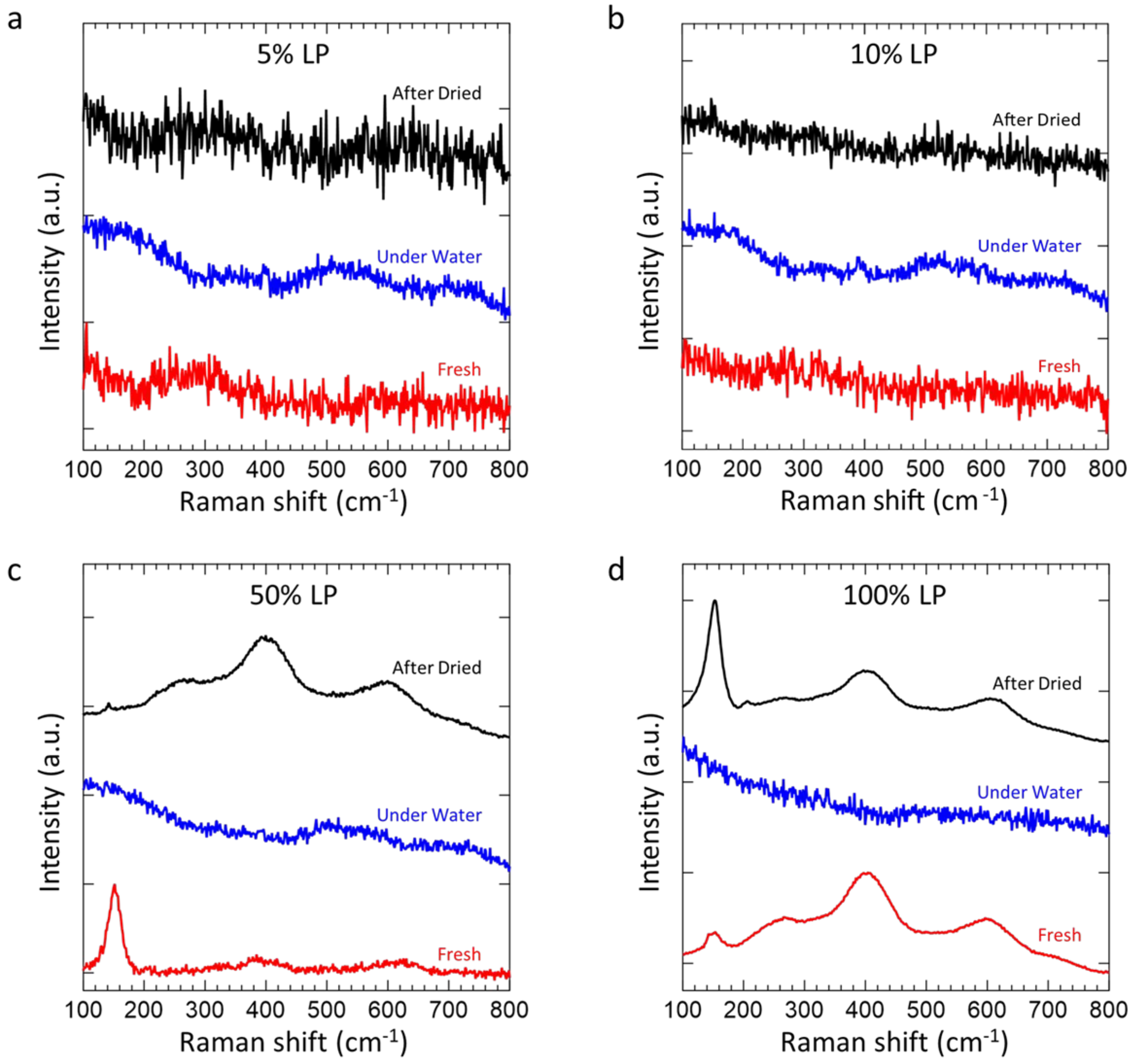

Figure S20. Raman spectra of $Ti_4N_3T_x$ MXene that is fresh (red), under water (blue), and after dried (black) obtained with 633 nm laser at **a.** 5% LP, **b.** 10% LP, **c.** 50% LP, and **d.** 100% LP. All spectra were collected using the 633 nm laser, 1800 lines/mm grating, and 50x objective lens.

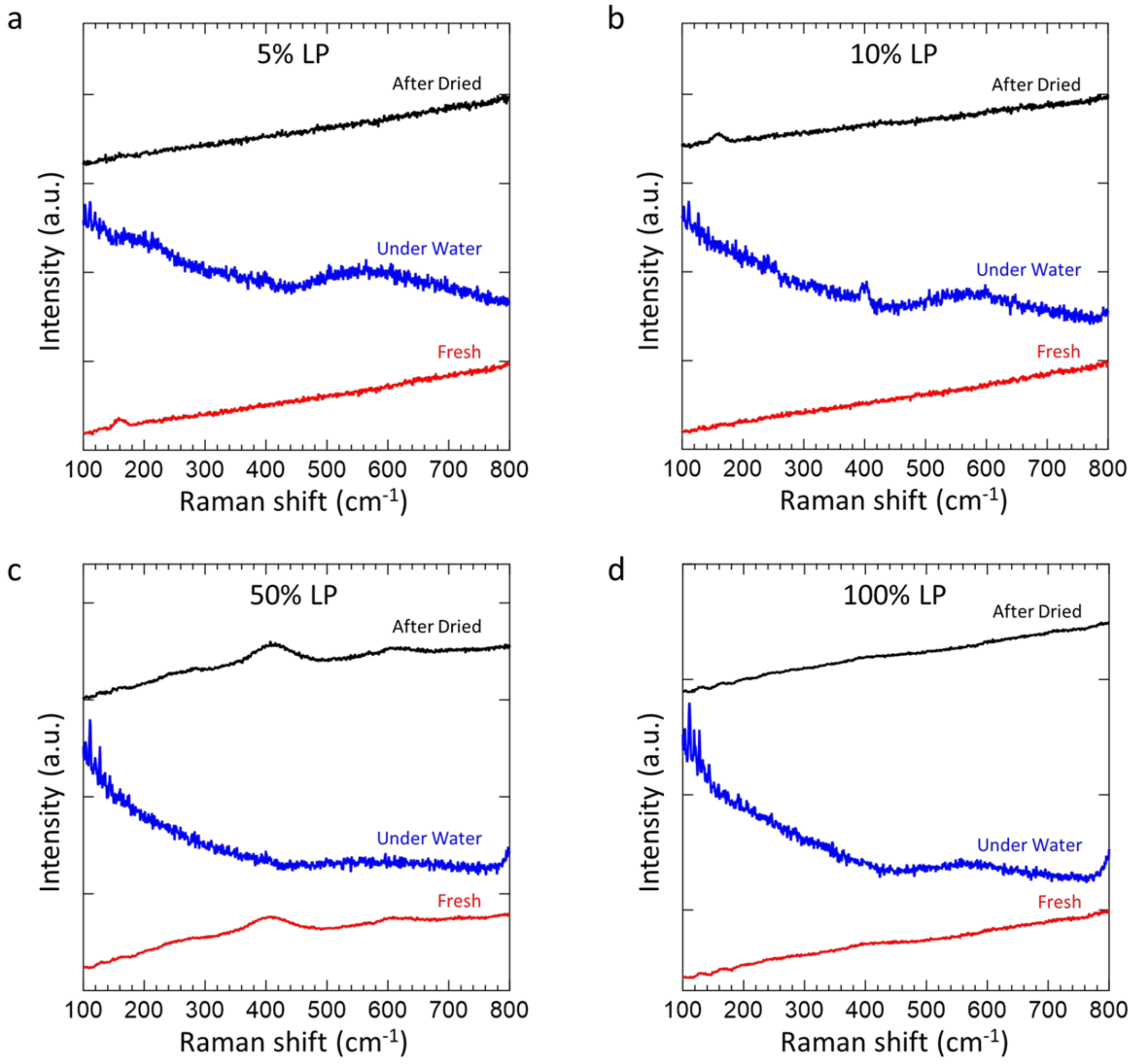

Figure S21. Raman spectra of $Ti_4N_3T_x$ MXene that is fresh (red), under water (blue), and after dried (black) obtained with 785 nm laser at **a.** 5% LP, **b.** 10% LP, **c.** 50% LP, and **d.** 100% LP. All spectra were collected using the 785 nm laser, 1800 lines/mm grating, and 50x objective lens.

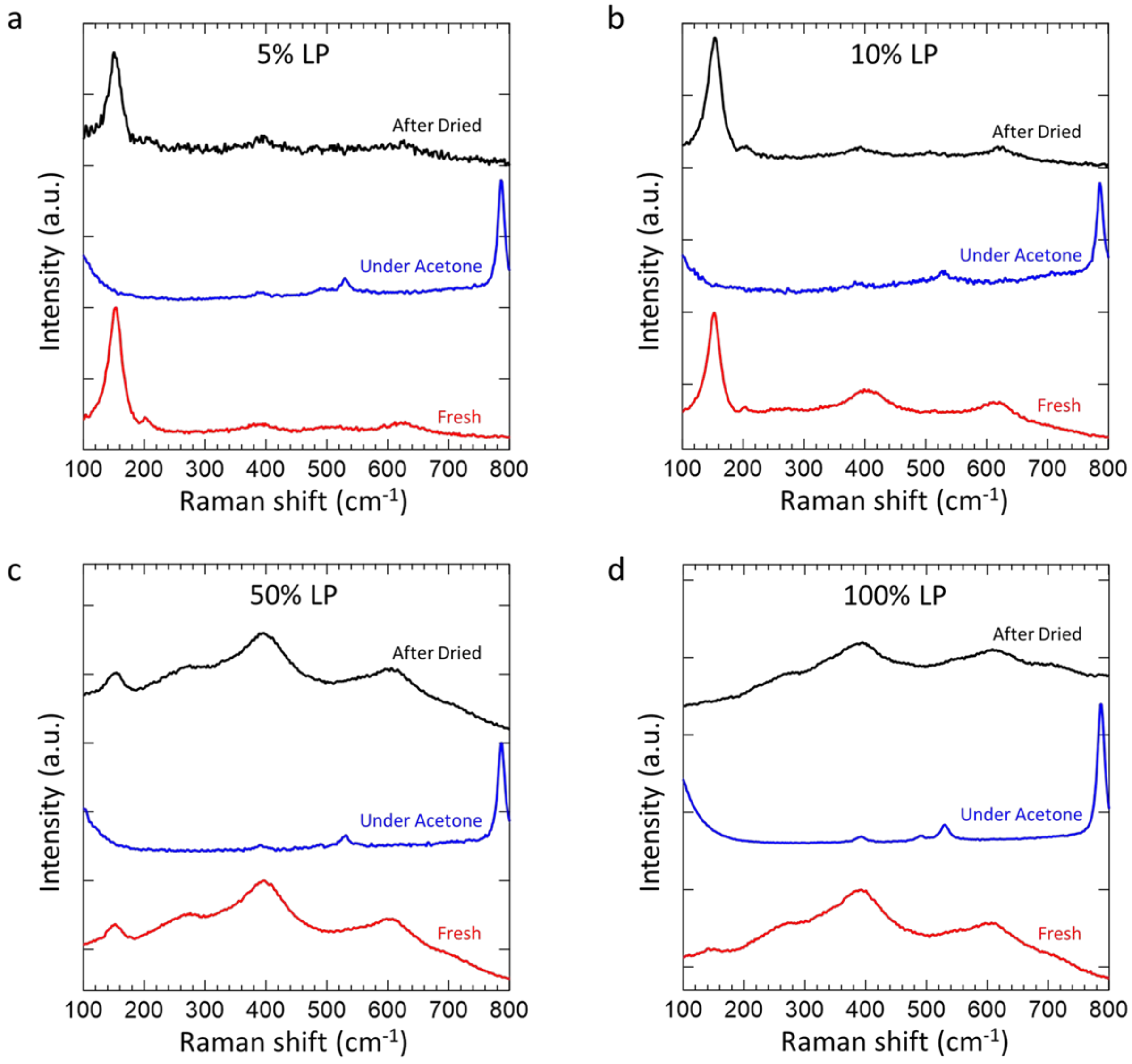

Figure S22. Raman spectra of $Ti_4N_3T_x$ MXene that is fresh (red), under acetone (blue), and after dried (black) obtained with 532 nm laser at **a.** 5% LP, **b.** 10% LP, **c.** 50% LP, and **d.** 100% LP. All spectra were collected using the 532 nm laser, 1800 lines/mm grating, and 50x objective lens.

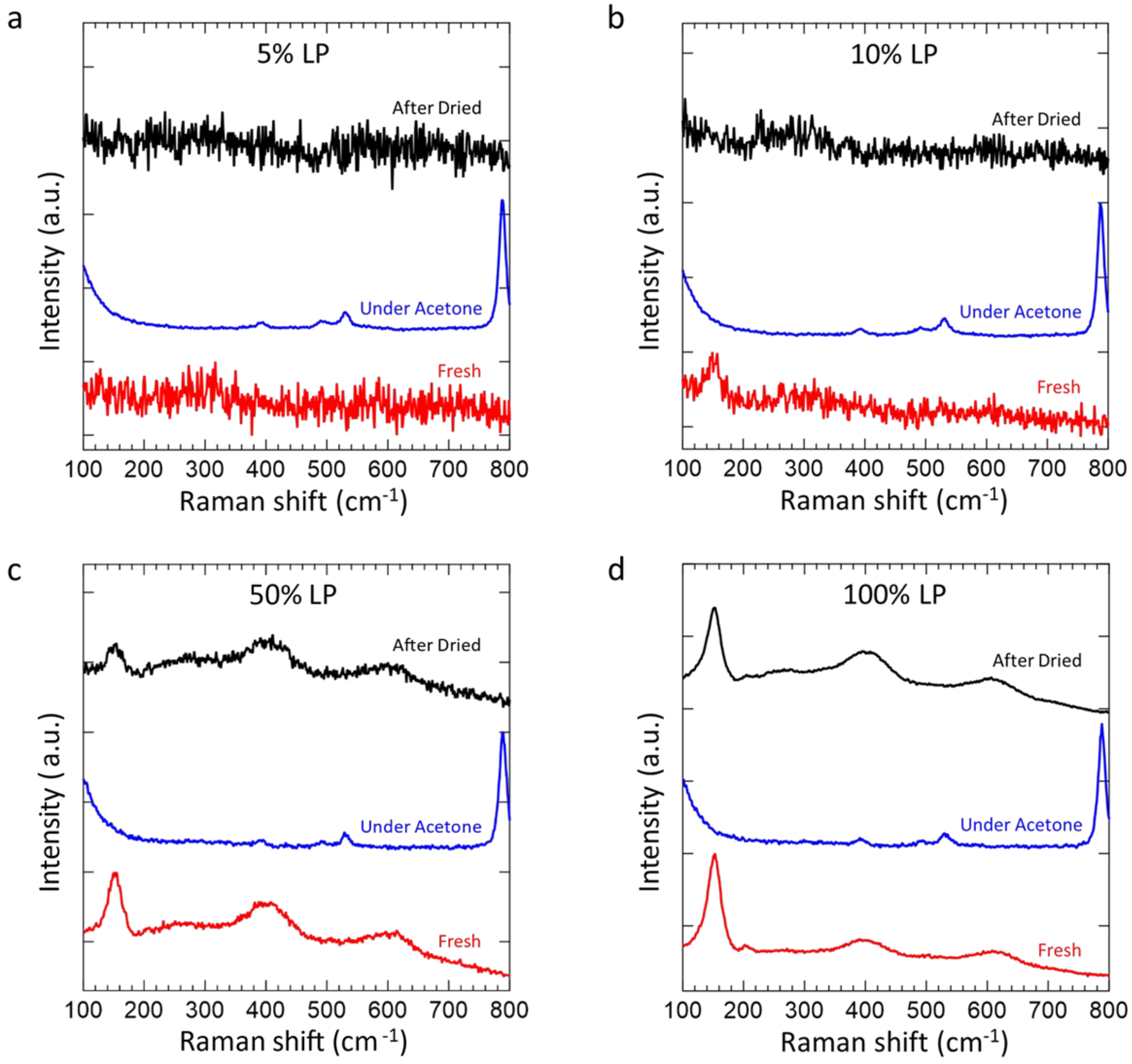

Figure S23. Raman spectra of $Ti_4N_3T_x$ MXene that is fresh (red), under acetone (blue), and after dried (black) obtained with 633 nm laser at **a.** 5% LP, **b.** 10% LP, **c.** 50% LP, and **d.** 100% LP. All spectra were collected using the 633 nm laser, 1800 lines/mm grating, and 50x objective lens.

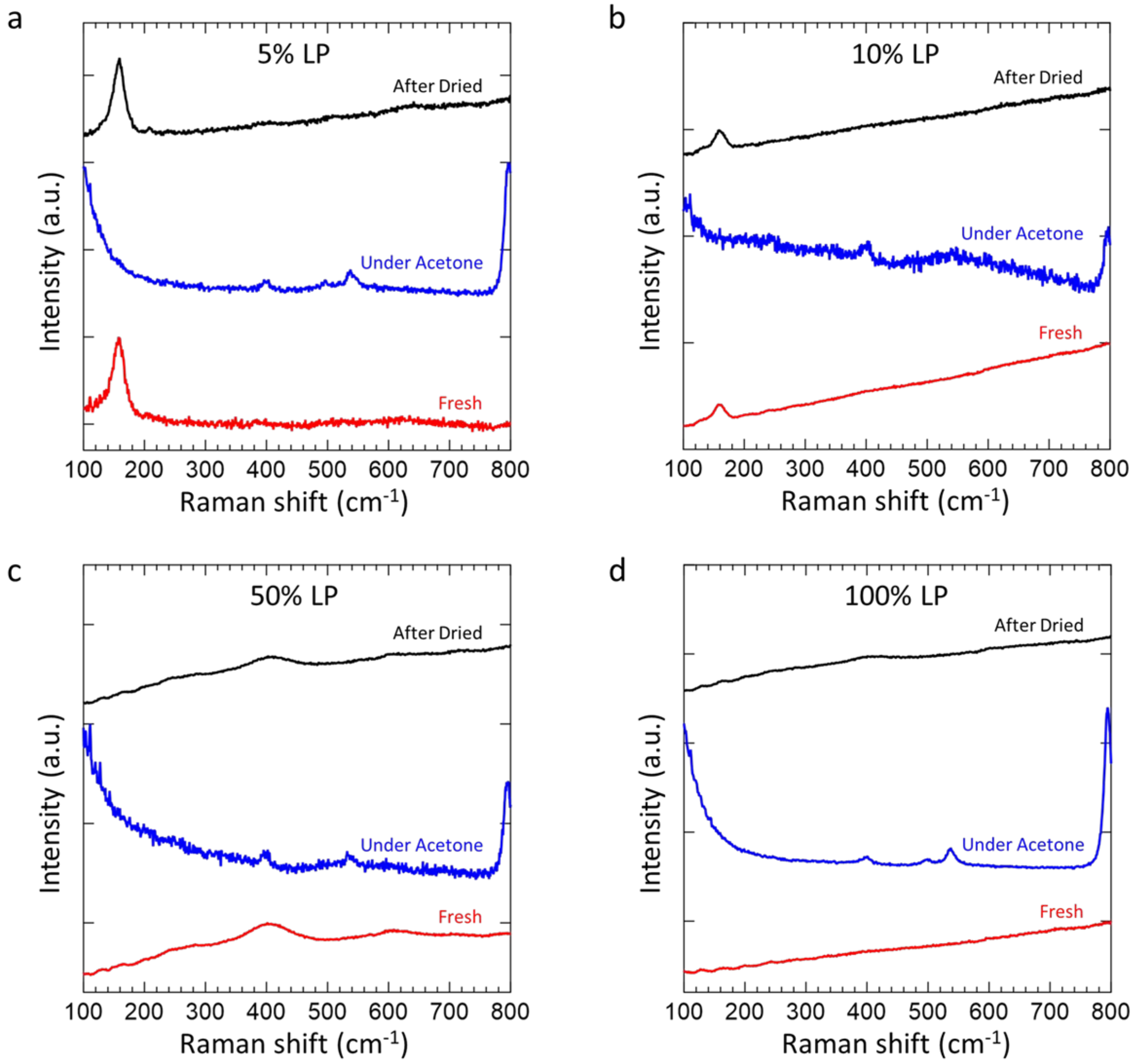

Figure S24. Raman spectra of $Ti_4N_3T_x$ MXene that is fresh (red), under acetone (blue), and after dried (black) obtained with 785 nm laser at **a.** 5% LP, **b.** 10% LP, **c.** 50% LP, and **d.** 100% LP. All spectra were collected using the 785 nm laser, 1800 lines/mm grating, and 50x objective lens.

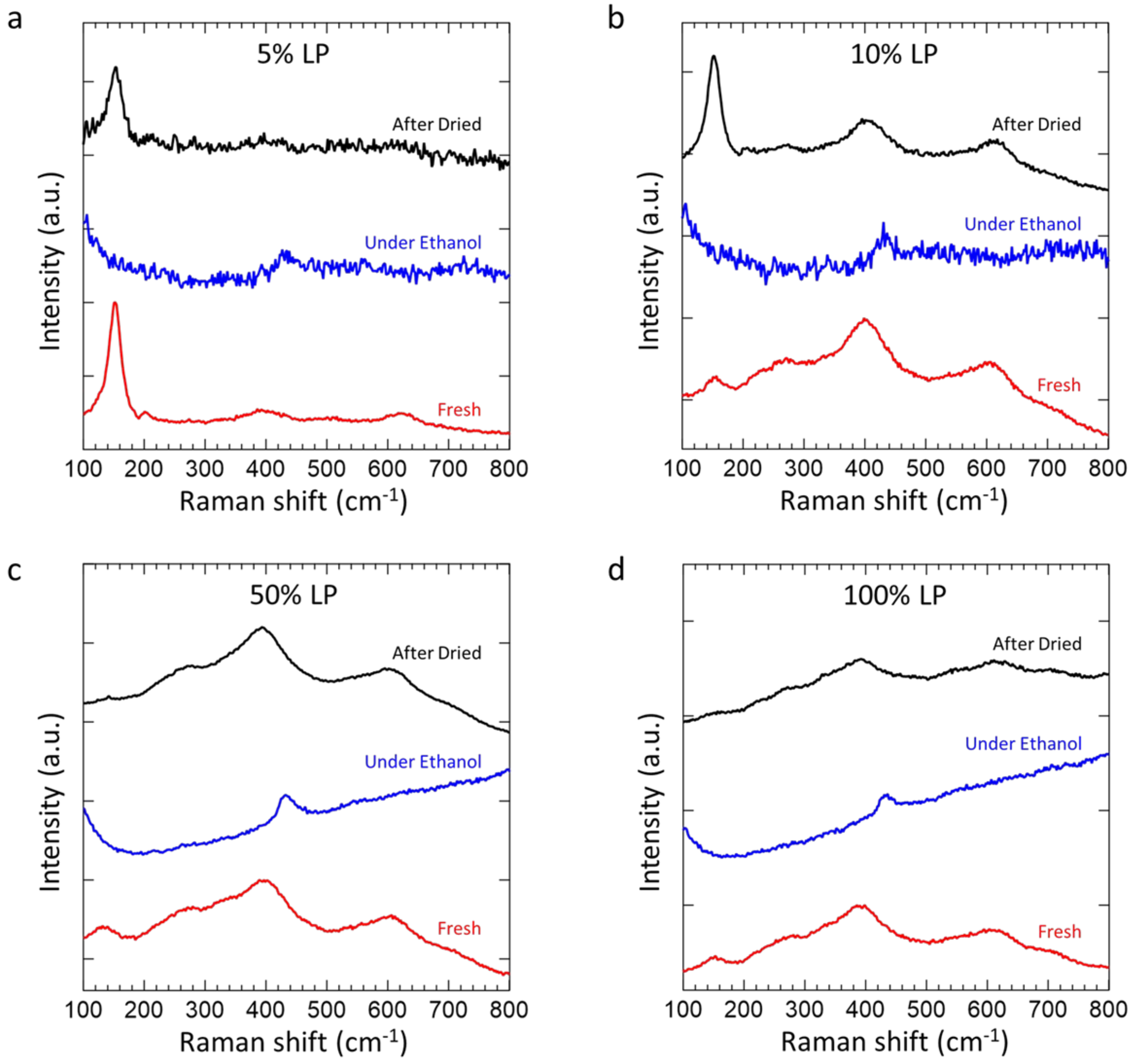

Figure S25. Raman spectra of $Ti_4N_3T_x$ MXene that is fresh (red), under ethanol (blue), and after dried (black) obtained with 532 nm laser at **a.** 5% LP, **b.** 10% LP, **c.** 50% LP, and **d.** 100% LP. All spectra were collected using the 532 nm laser, 1800 lines/mm grating, and 50x objective lens.

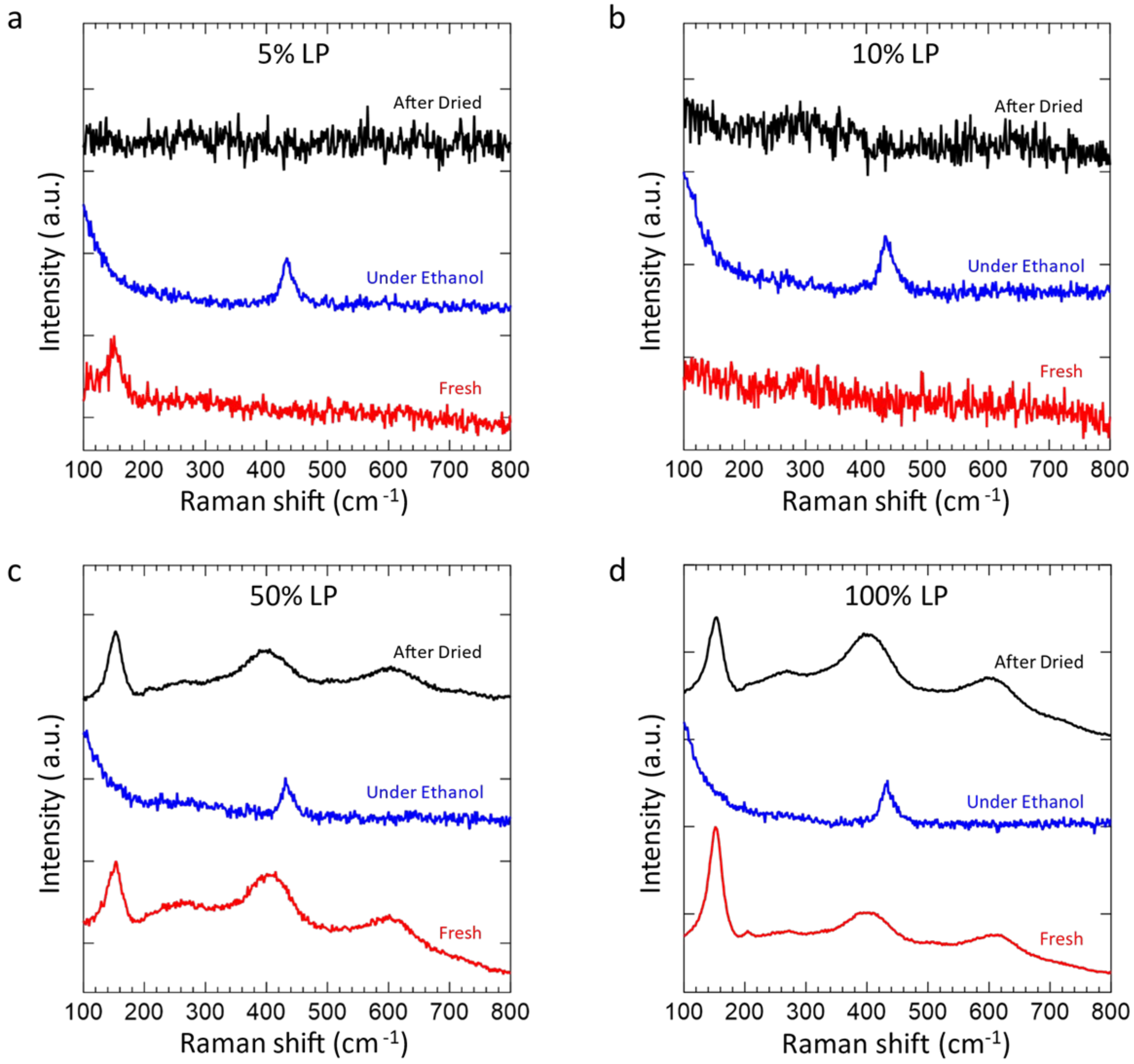

Figure S26. Raman spectra of $Ti_4N_3T_x$ MXene that is fresh (red), under ethanol (blue), and after dried (black) obtained with 633 nm laser at **a.** 5% LP, **b.** 10% LP, **c.** 50% LP, and **d.** 100% LP. All spectra were collected using the 633 nm laser, 1800 lines/mm grating, and 50x objective lens.

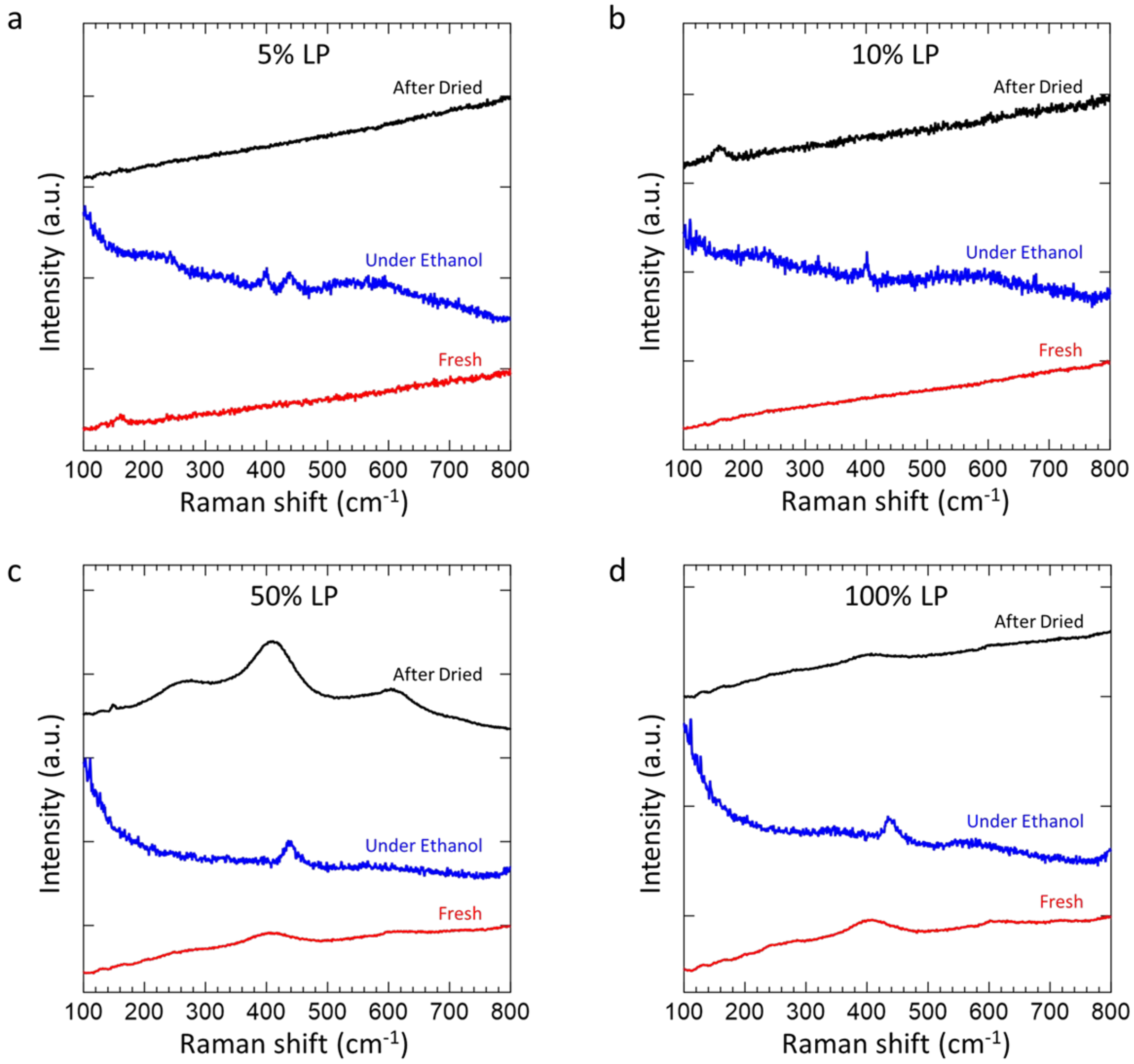

Figure S27. Raman spectra of $Ti_4N_3T_x$ MXene that is fresh (red), under ethanol (blue), and after dried (black) obtained with 785 nm laser at **a.** 5% LP, **b.** 10% LP, **c.** 50% LP, and **d.** 100% LP. All spectra were collected using the 785 nm laser, 1800 lines/mm grating, and 50x objective lens.

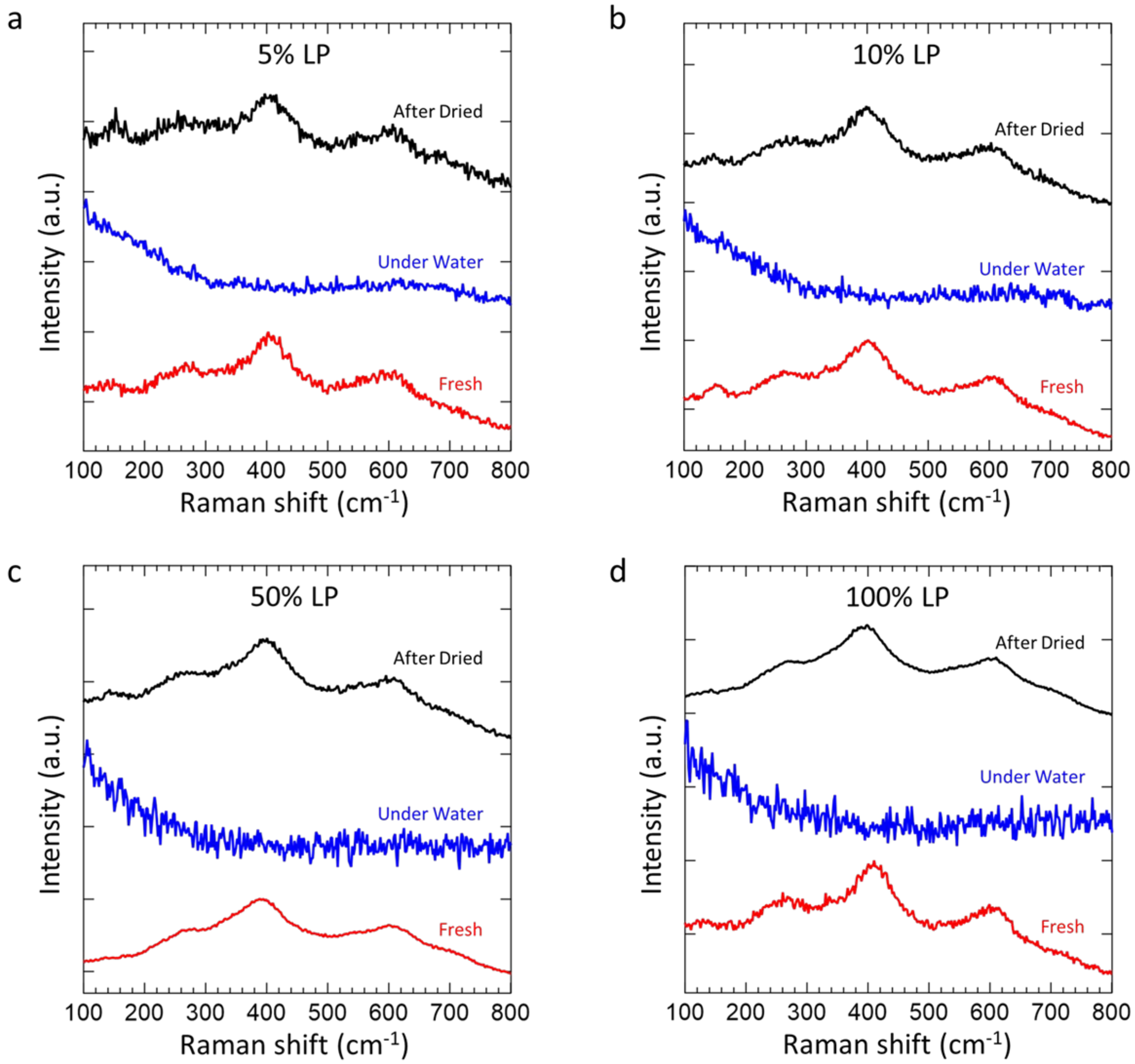

Figure S28. Raman spectra of Ti_3CNT_x MXene that is fresh (red), under water (blue), and after dried (black) obtained with 532 nm laser at **a.** 5% LP, **b.** 10% LP, **c.** 50% LP, and **d.** 100% LP. All spectra were collected using the 532 nm laser, 1800 lines/mm grating, and 50x objective lens.

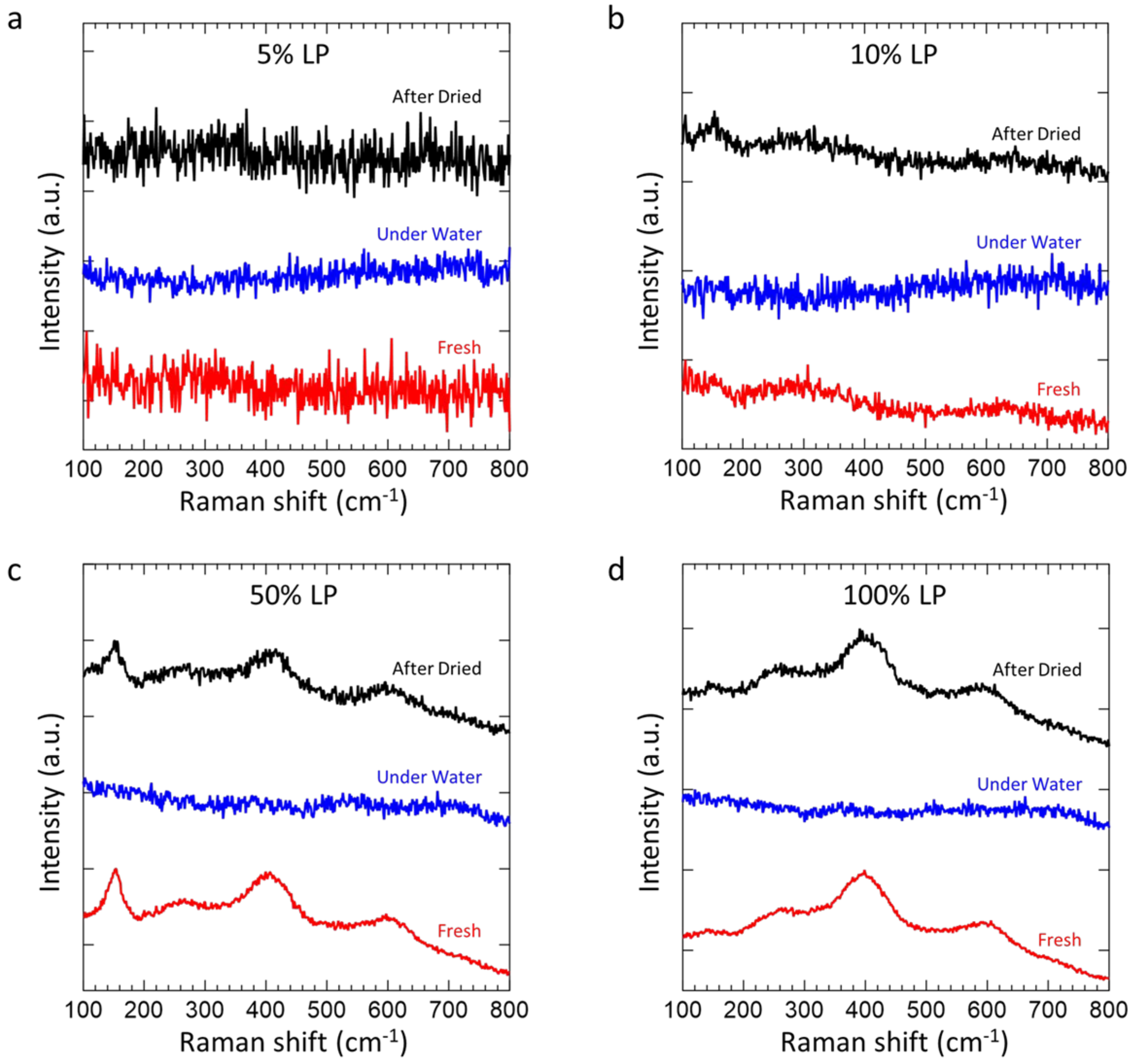

Figure S29. Raman spectra of Ti_3CNT_x MXene that is fresh (red), under water (blue), and after dried (black) obtained with 633 nm laser at **a.** 5% LP, **b.** 10% LP, **c.** 50% LP, and **d.** 100% LP. All spectra were collected using the 633 nm laser, 1800 lines/mm grating, and 50x objective lens.

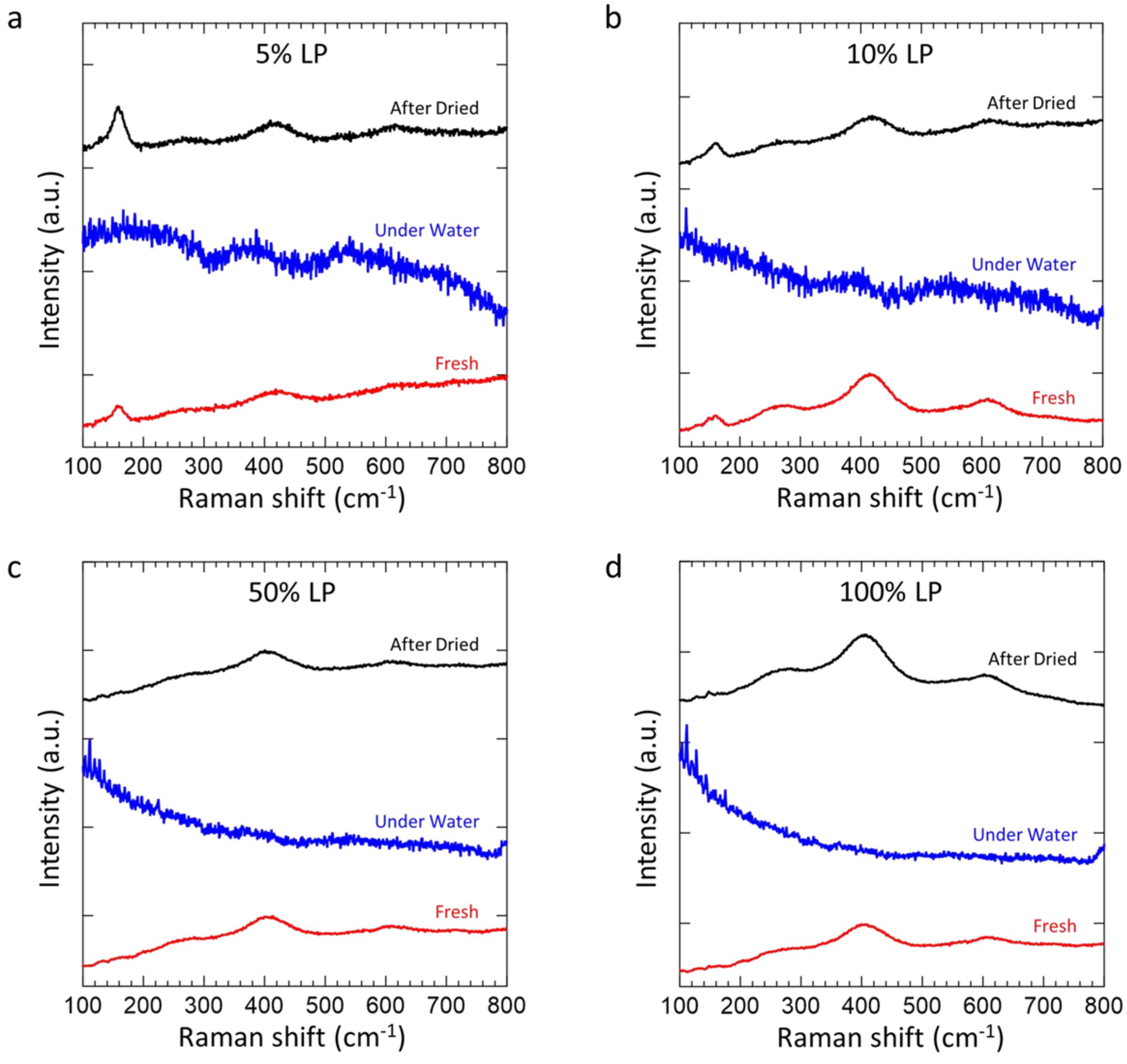

Figure S30. Raman spectra of Ti_3CNT_x MXene that is fresh (red), under water (blue), and after dried (black) obtained with 785 nm laser at **a.** 5% LP, **b.** 10% LP, **c.** 50% LP, and **d.** 100% LP. All spectra were collected using the 785 nm laser, 1800 lines/mm grating, and 50x objective lens.

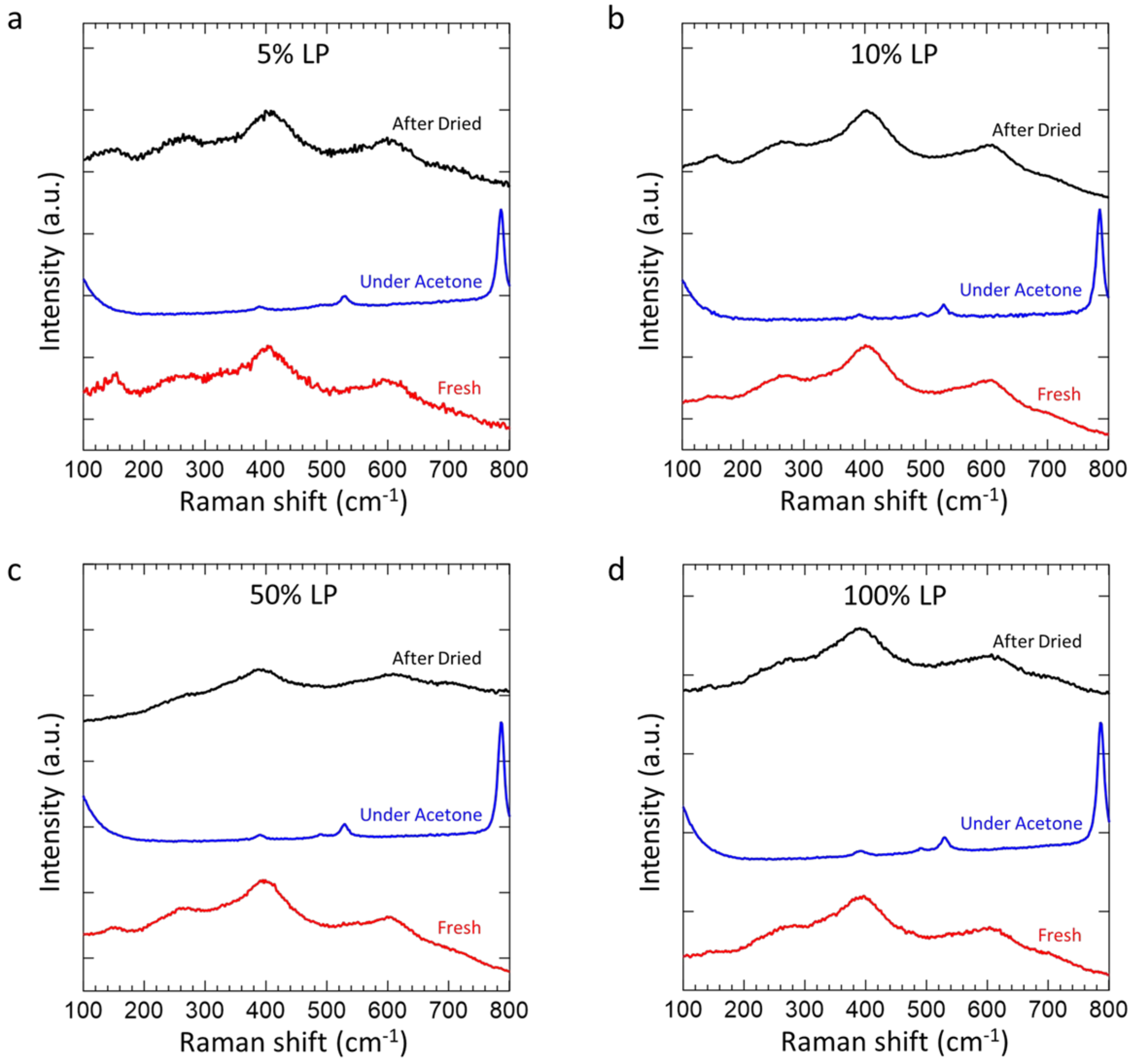

Figure S31. Raman spectra of Ti_3CNT_x MXene that is fresh (red), under acetone (blue), and after dried (black) obtained with 532 nm laser at **a.** 5% LP, **b.** 10% LP, **c.** 50% LP, and **d.** 100% LP. All spectra were collected using the 532 nm laser, 1800 lines/mm grating, and 50x objective lens.

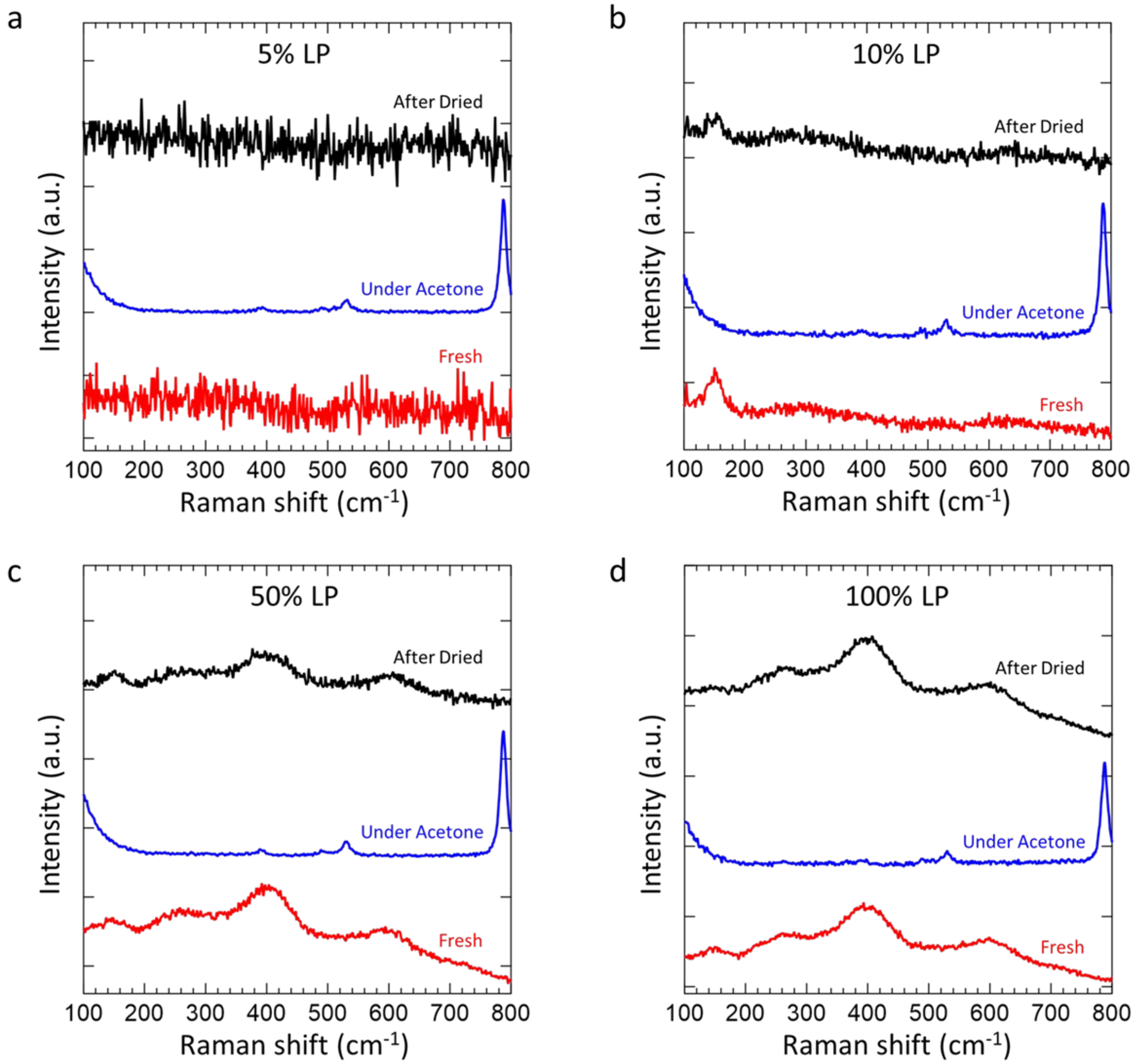

Figure S32. Raman spectra of Ti_3CNT_x MXene that is fresh (red), under acetone (blue), and after dried (black) obtained with 633 nm laser at **a.** 5% LP, **b.** 10% LP, **c.** 50% LP, and **d.** 100% LP. All spectra were collected using the 633 nm laser, 1800 lines/mm grating, and 50x objective lens.

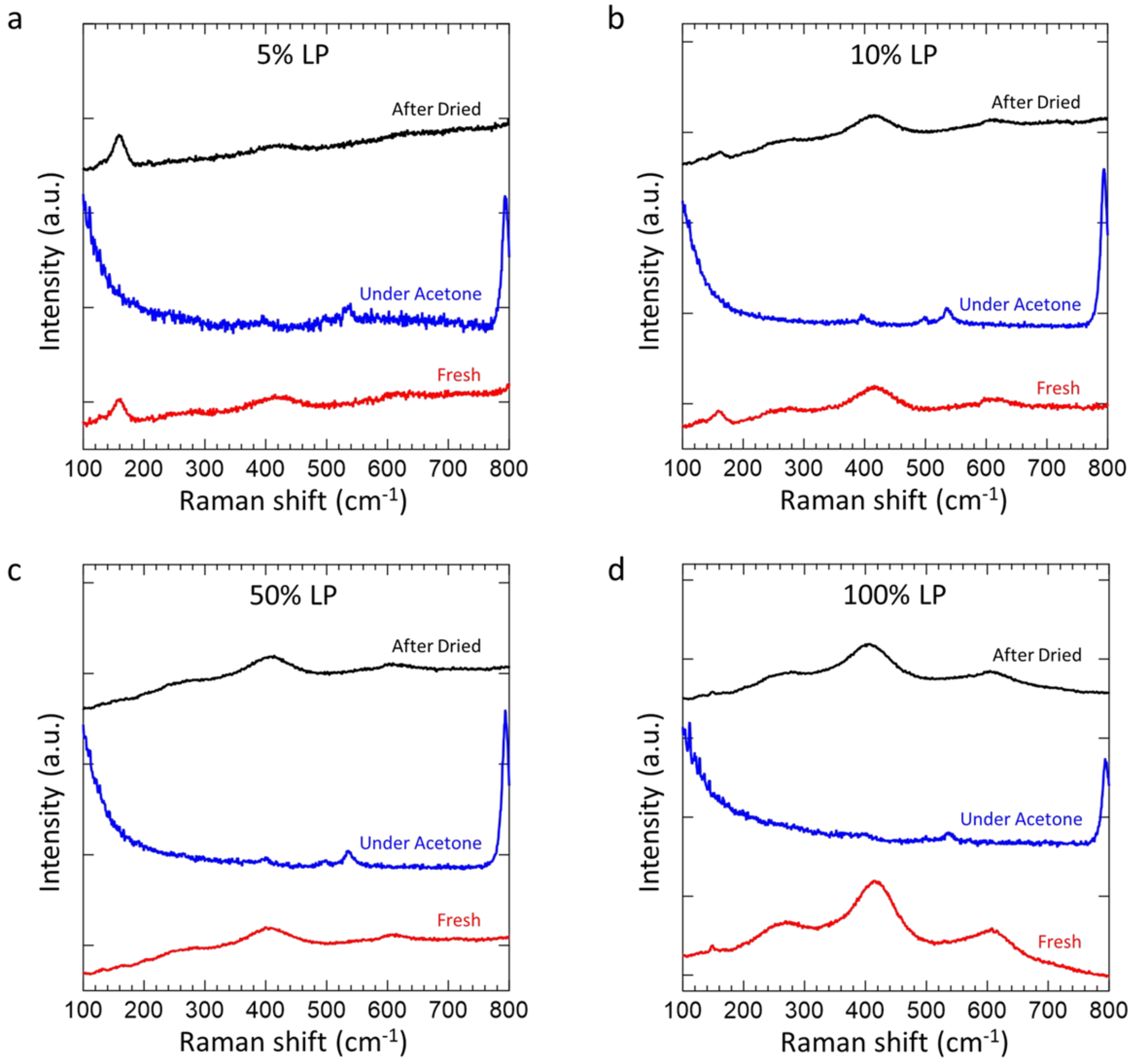

Figure S33. Raman spectra of Ti_3CNT_x MXene that is fresh (red), under acetone (blue), and after dried (black) obtained with 785 nm laser at **a.** 5% LP, **b.** 10% LP, **c.** 50% LP, and **d.** 100% LP. All spectra were collected using the 785 nm laser, 1800 lines/mm grating, and 50x objective lens.

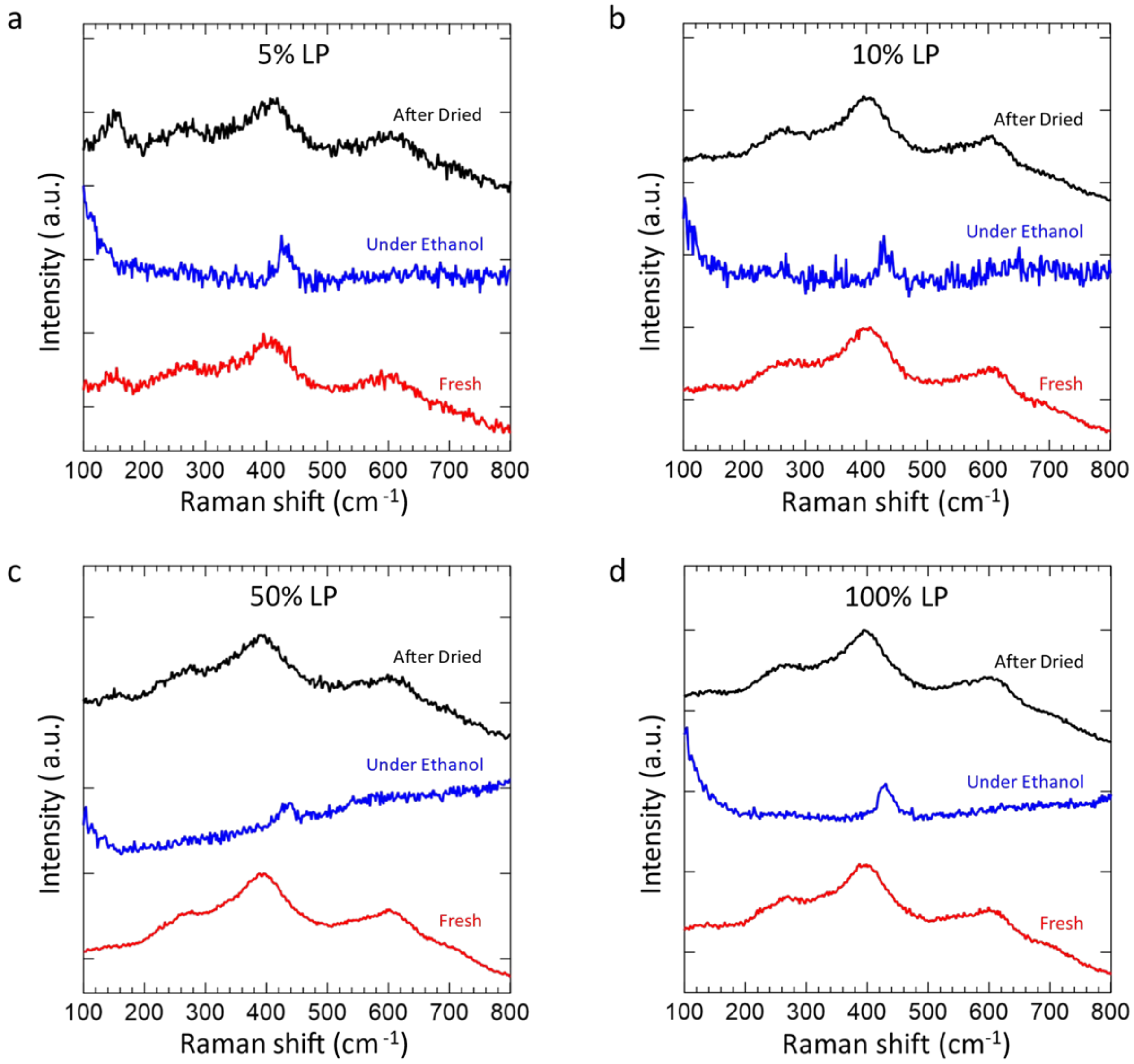

Figure S34. Raman spectra of Ti_3CNT_x MXene that is fresh (red), under ethanol (blue), and after dried (black) obtained with 532 nm laser at **a.** 5% LP, **b.** 10% LP, **c.** 50% LP, and **d.** 100% LP. All spectra were collected using the 532 nm laser, 1800 lines/mm grating, and 50x objective lens.

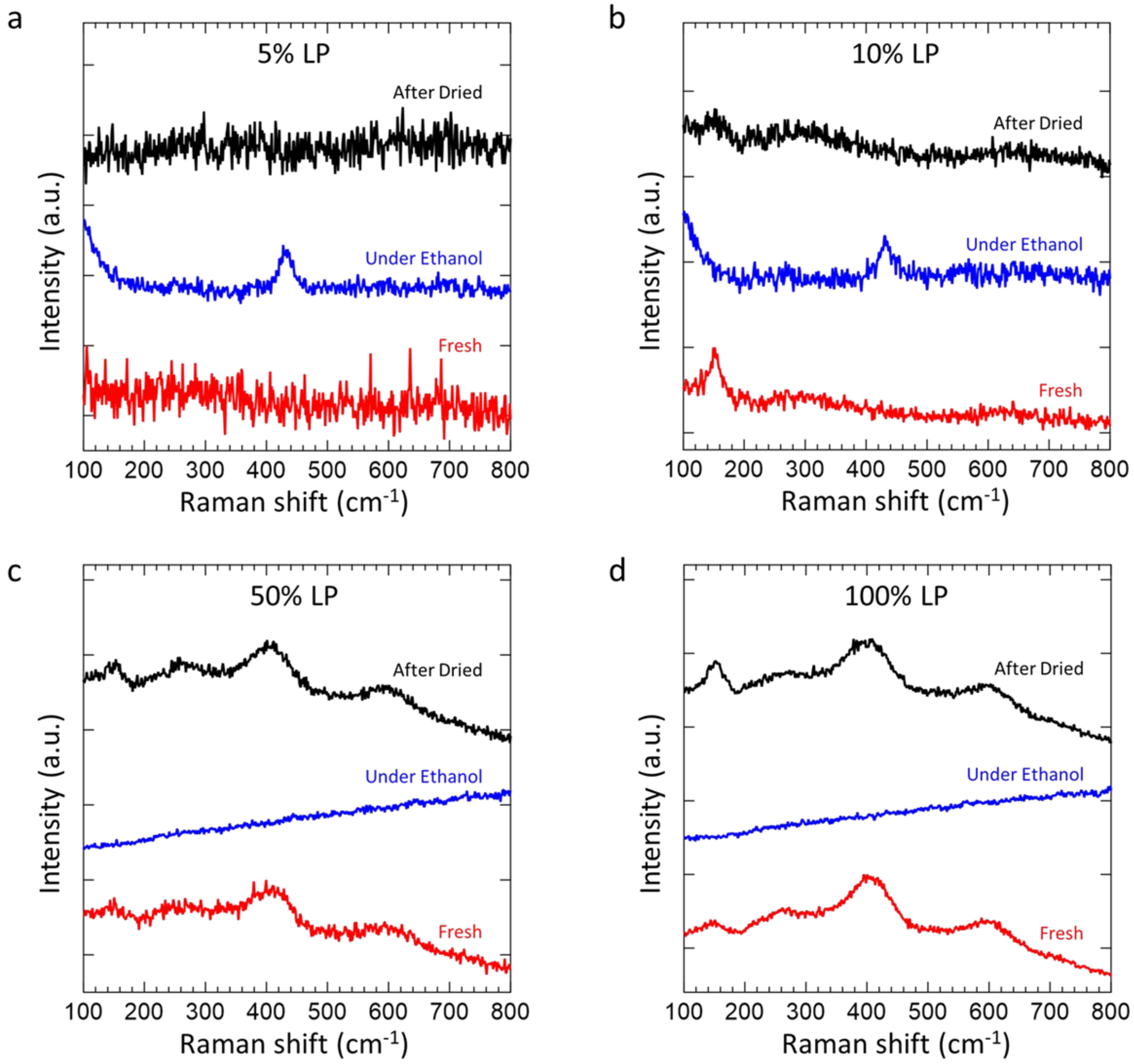

Figure S35. Raman spectra of Ti_3CNT_x MXene that is fresh (red), under ethanol (blue), and after dried (black) obtained with 633 nm laser at **a.** 5% LP, **b.** 10% LP, **c.** 50% LP, and **d.** 100% LP. All spectra were collected using the 633 nm laser, 1800 lines/mm grating, and 50x objective lens.

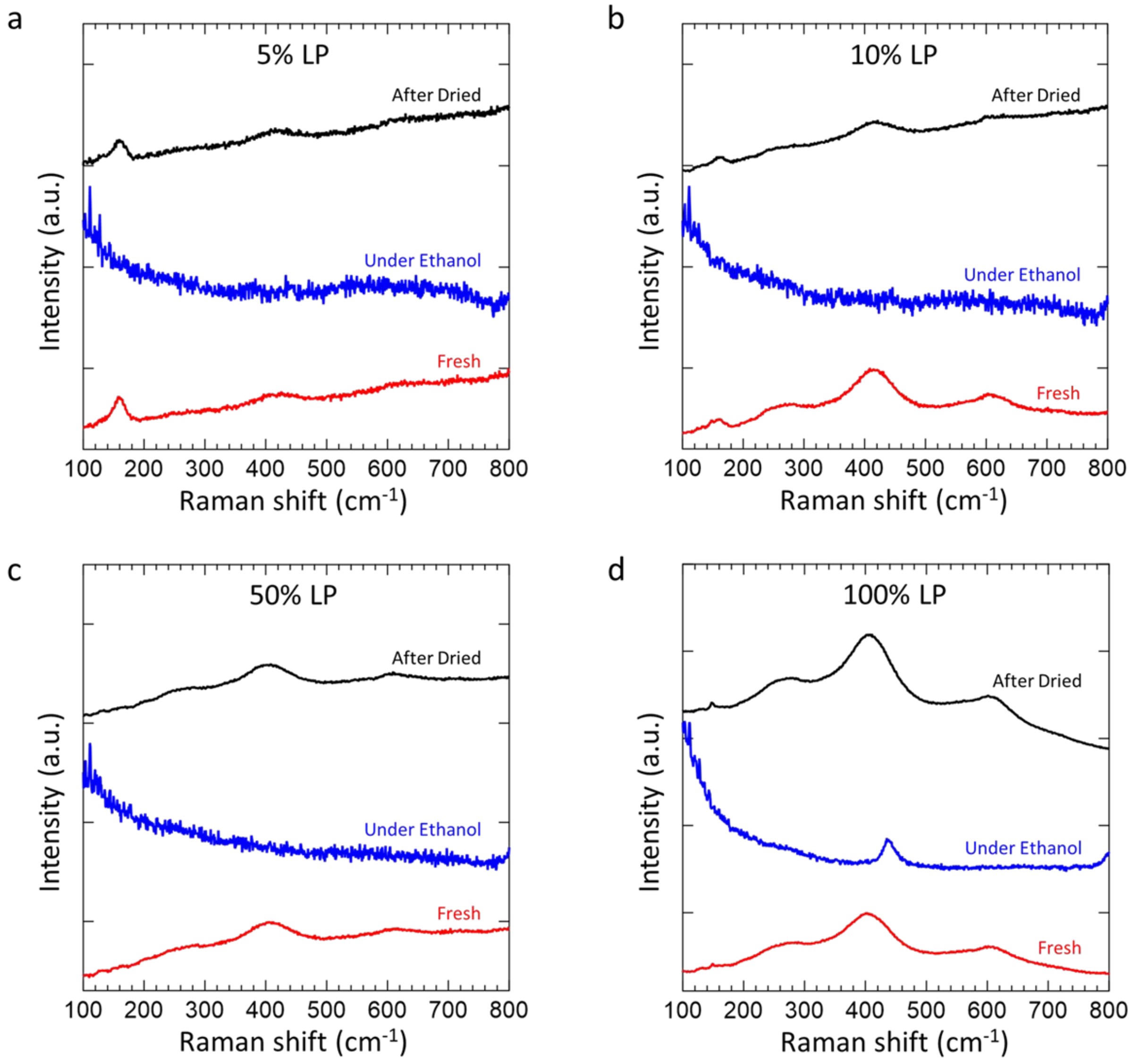

Figure S36. Raman spectra of Ti_3CNT_x MXene that is fresh (red), under ethanol (blue), and after dried (black) obtained with 785 nm laser at **a.** 5% LP, **b.** 10% LP, **c.** 50% LP, and **d.** 100% LP. All spectra were collected using the 785 nm laser, 1800 lines/mm grating, and 50x objective lens.

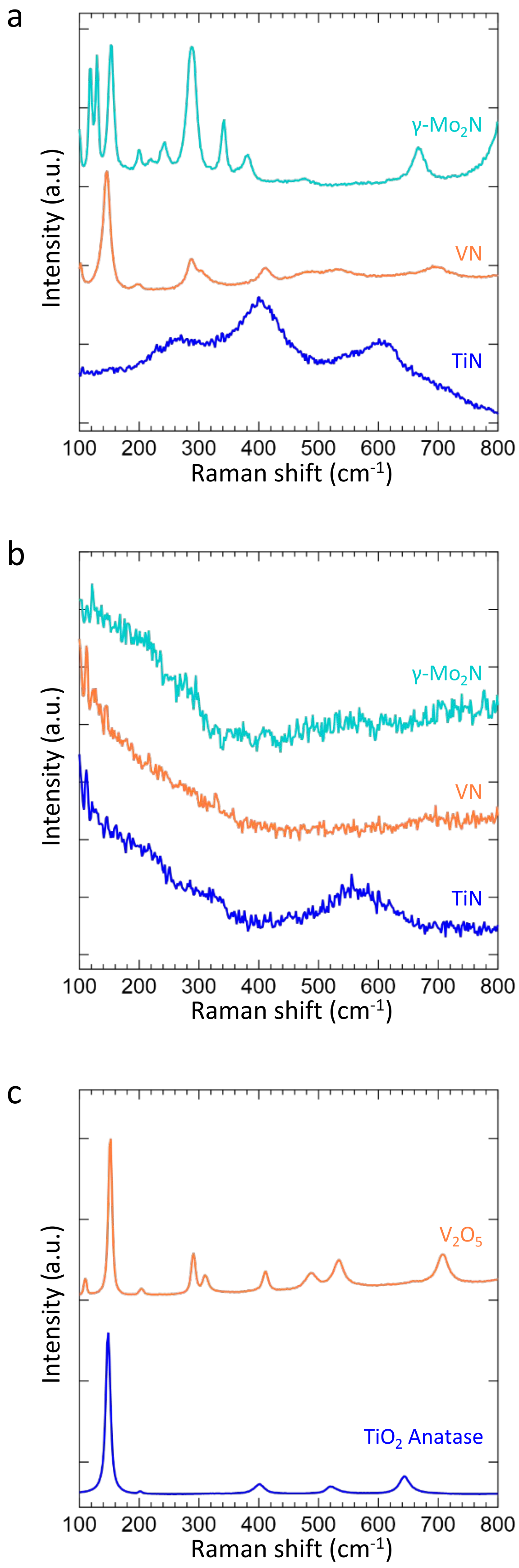
a
b
c
γ-Mo$_2$N
VN
TiN
V$_2$O$_5$
TiO$_2$ Anatase
Intensity (a.u.)
Raman shift (cm^{-1})
100 200 300 400 500 600 700 800

Figure S37. Raman spectra of **a.** bulk TMNs: TiN (blue), VN (orange), and γ-Mo_2N (turquoise) and **b.** TMNs and **c.** corresponding oxides TiO_2 anatase (blue) and V_2O_5 (orange) under water. Spectra for VN and γ-Mo_2N show the passivated oxide surface. Raman spectra were collected using a 532 nm laser, 1800 lines/mm grating, and 50x objective lens. Spectra for TMNs and corresponding oxides were collected at 10% laser power for comparison. All of the data corroborate that TMNs are observed to exhibit modified Raman activity and vibrational behavior under water, and that nitrogen in the material structure is the root cause.

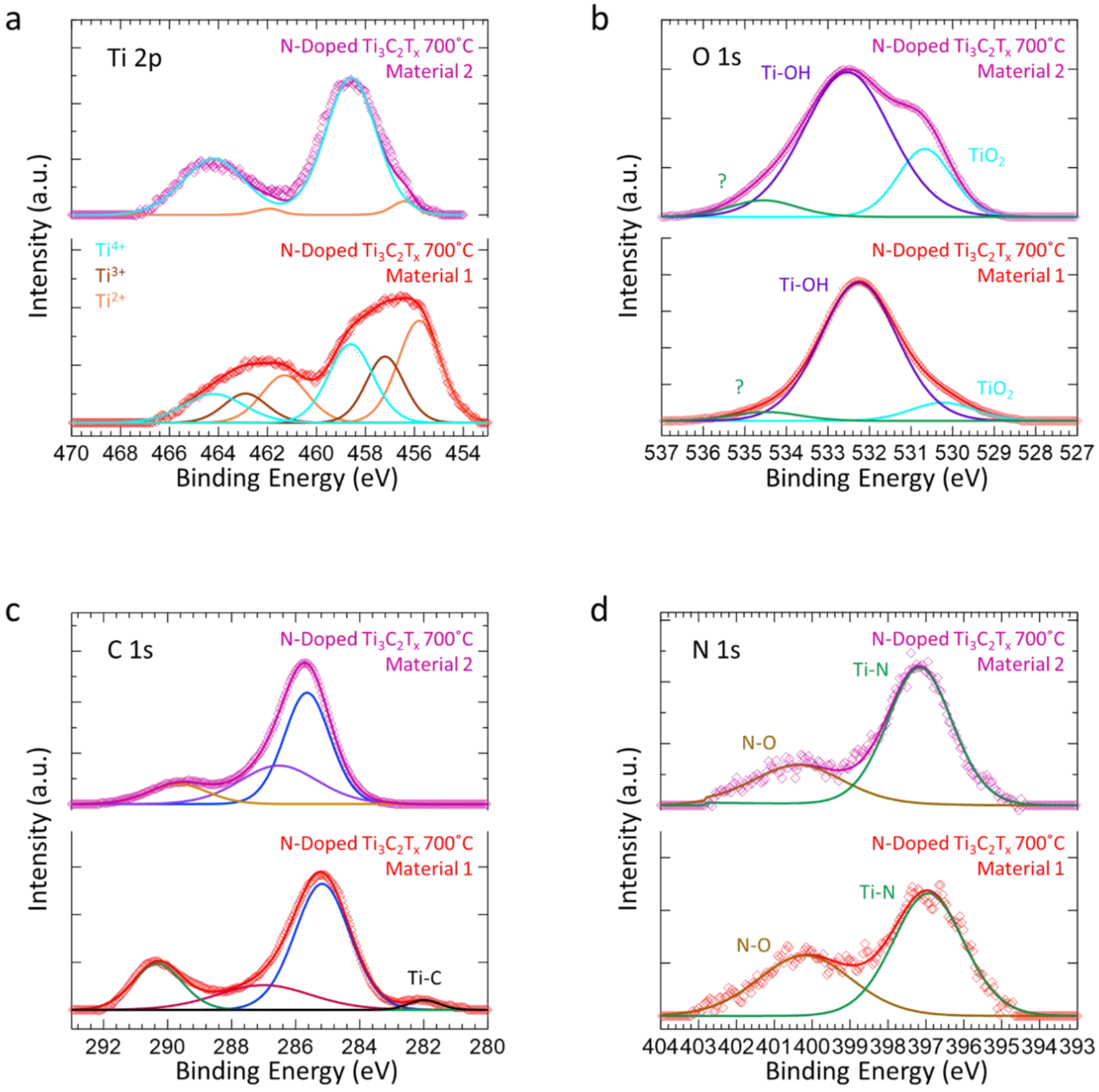

Figure S38. a. Ti 2p, **b.** O 1s, **c.** C 1s, and **d.** N 1s XPS spectra of two N-doped $Ti_3C_2T_x$ MXene synthesized from ammonization at 700˚C, indicating the presence of a Ti-N bond in the MXene after ammonization. The structural inhomogeneity of the N-doped $Ti_3C_2T_x$ MXene from ammonization at 700˚C is illustrated. All of the XPS data indicate that the nitrogen doping into the $Ti_3C_2T_x$ MXene is successful.

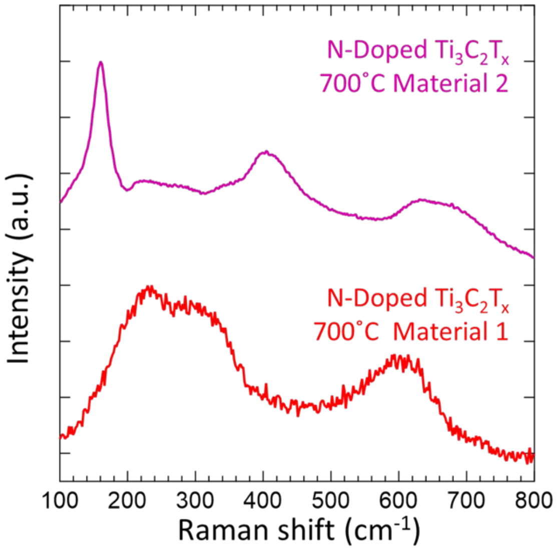

Figure S39. Raman spectra of two N-doped $Ti_3C_2T_x$ MXene materials synthesized from ammonization at 700˚C, indicating a structural change from the starting $Ti_3C_2T_x$ MXene. Spectra were collected at 10% laser power for comparison. All spectra were collected using a 532 nm laser, 1800 lines/mm grating, and 50x objective lens.

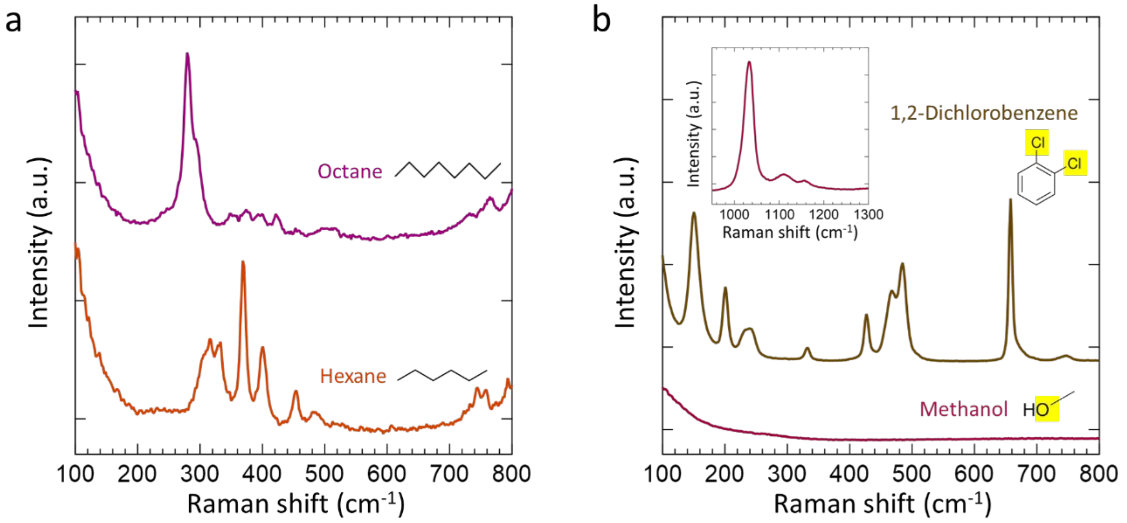

Figure S40. Raman spectra of the following solvents used in the study: **a.** nonpolar hydrocarbon solvents (hexane (orange) and octane (magenta)) and **b.** methanol (magenta) and 1,2-dichlorobenzene (brown). An inset is provided to show the Raman active features of the methanol spectrum. All spectra were obtained at 100% laser power. Spectra were collected using a 532 nm laser, 1800 lines/mm grating, and 50x objective lens.

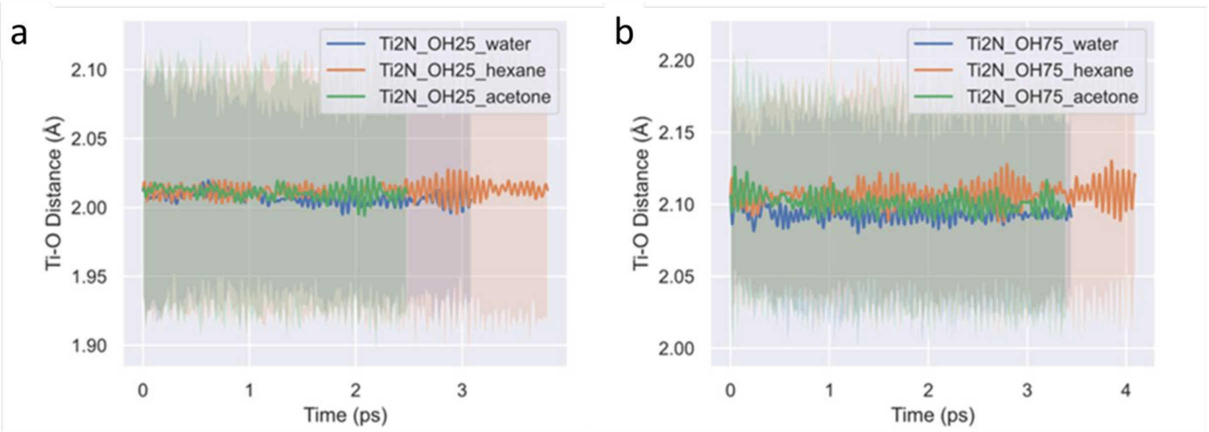

Figure S41. Evolution of Ti-N and Ti-O bond distances for Ti_2NT_x surfaces in various solvents (cutoff radius: 2.6 Å). Ti-O and bond distance evolution of **a.** Ti_2NT_x basal surface (25% –OH coverage) and **b.** Ti_2NT_x basal surface (75% –OH coverage). Blue, orange, and green lines represent average bond lengths in water, hexane, and acetone, respectively. Shaded areas in corresponding colors indicate the standard deviation of bond distances. Noted that bottom fixed surfaces and their terminations were excluded from the analysis. The Ti-O bond lengths vary slightly following the trend: hexane > acetone > water.
